# Breastfeeding and the risk of childhood cancer: a systematic review and dose-response meta-analysis

**DOI:** 10.1186/s12916-021-01950-5

**Published:** 2021-04-13

**Authors:** Qing Su, Xiaohui Sun, Liwen Zhu, Qin Yan, Peiwen Zheng, Yingying Mao, Ding Ye

**Affiliations:** 1 Department of Epidemiology and Biostatistics, School of Public Health, Zhejiang Chinese Medical University, 548 Binwen Road, Hangzhou, 310053 China; 2 Department of Hematology and Oncology, Children’s hospital of Nanjing Medical University, Nanjing, 210008 China; 3 Department of Medical Adiministration, Women’s Hospital, Zhejiang University School of Medicine, Hangzhou, 310006 China

**Keywords:** Breastfeeding, Childhood cancer, Meta-analysis, Dose-response

## Abstract

**Background:**

The aim of this study was to quantitatively summarize the available evidence on the association of breastfeeding with the risk of childhood cancer.

**Methods:**

A literature search of PubMed and Embase databases was performed to identify eligible observational studies published from inception to July 17, 2020. The categorical and dose-response meta-analysis was conducted by pooling relative risk (RR) or odds ratio (OR) estimates with 95% confidence intervals (CIs). Potential sources of heterogeneity were detected by meta-regression and stratification analysis. Sensitivity analysis and publication bias test were also carried out.

**Results:**

Forty-five articles involving 475,579 individuals were included in the meta-analysis. Among the thirty-three studies on the association between breastfeeding and risk of childhood leukemia, the pooled risk estimates were 0.77 (95% CI, 0.65–0.91) and 0.77 (95% CI 0.63–0.94) for ever versus non/occasional breastfeeding and longest versus shortest breastfeeding duration group, respectively. There was clear indication for non-linear dose-response relationship between breastfeeding duration and the risk of childhood leukemia (*P* non-linear < 0.001). The most protective effect (OR, 0.66, 95% CI 0.62–0.70) was observed at a breastfeeding duration of 9.6 months. Four studies examined, the association between breastfeeding and risk of childhood neuroblastoma, and significant inverse associations were consistently observed in both the comparisons of ever breastfeeding versus non/occasional breastfeeding (OR = 0.59, 95% CI 0.44–0.81) and longest versus shortest breastfeeding (OR = 0.61, 95% CI 0.44–0.83). However, no associations of breastfeeding with risk of other cancers were found.

**Conclusions:**

Our study supports a protective role of breastfeeding on the risk of childhood leukemia, also suggesting a non-linear dose-response relationship. Further studies are warranted to confirm the association between breastfeeding and risk of childhood neuroblastoma.

**Supplementary Information:**

The online version contains supplementary material available at 10.1186/s12916-021-01950-5.

## Background

Childhood cancer is emerging as a major cause of death in children worldwide, which is bleaker for children with cancer in lower-middle-income countries [1]. Although childhood cancer only accounted for 1% of the total cancer [2], while once it occurs, a range of medical, psychological, ethical, and societal concerns are raised. Moreover, the global age-standardized incidence rates of registered cancers in children aged 0–14 years have increased from 124.0 to 140.6 per million person-years since the 1980s [3]. To date, little is known about the etiology of childhood cancer, but maternal reproductive health are potential explanations for a fraction of the incidence [4–6].

It is widely accepted that breastfeeding may protect mothers against breast cancer [7] and ovarian cancer [8], but also bring multiple health benefits for the infants [9, 10]. However, the relationships of breastfeeding with the risk of childhood cancer are inconsistent across studies and the associations may differ by cancer types. Several studies have shown that breastfeeding had a protective effect on childhood cancer and the protection increased with the duration of the breastfeeding [11, 12]. However, some previous studies showed no evidence of protection from breastfeeding for childhood cancer, and the analyses by duration of breastfeeding also failed to support the protective hypothesis [13, 14]. When specific types of childhood cancer were examined, Amitay and Keinan-Boker [15] showed that breastfeeding was inversely associated with the risk of childhood leukemia. However, Wang et al. [16] provided limited evidence for a protective role of breastfeeding in childhood Hodgkin's lymphoma. Other publications have even reported that prolonged breastfeeding was positively associated with the risk of childhood malignant germ cell tumors [17] and leukemia [18].

A previous meta-analysis of the association between breastfeeding and the risk of childhood cancer was based on 26 original studies published up to June 2004 [19]. The pooled effect estimates suggested that breastfeeding was associated with 9% (95% confidence interval (CI), 2–16%) lower risk of acute lymphoblastic leukemia (ALL), 24% (95% CI 3–40%) lower risk of Hodgkin’s disease, and 41% (95% CI 22–56%) lower risk of neuroblastoma, but no associations of breastfeeding with acute nonlymphoblastic leukemia, non-Hodgkin’s lymphoma, central nervous system cancers, malignant germ cell tumors, juvenile bone tumors, or other solid cancers. Since then, evidences regarding this association have accumulated rapidly and provided more answers to this question. For the dose-response relationship, a previous pooled analysis showed that the protective effect of breastfeeding on the risk of childhood ALL was lowest at the breastfeeding duration of 8–10 months [20]. However, the exact dose-response relationship has not yet been evaluated for other cancer types. Therefore, we conducted this updated systematic review and dose-response meta-analysis of epidemiological studies to quantify precisely the impact of breastfeeding on the incidence of childhood cancer.

## Methods

The study was registered in the international prospective register of systematic reviews (PROSPERO: CRD42020199446). The Preferred Reporting Items for Systematic reviews and Meta-Analysis (PRISMA) checklist for reporting the meta-analysis was shown in Additional file 1: Table S1.

### Search strategy

Original articles from PubMed and EMBASE databases were systematically searched from the inception to 17 July 2020 to identify potentially eligible studies on the association between breastfeeding and the risk of childhood cancer. The search strategy was as follows: (“child” OR “pediatric” OR “childhood” OR “children”) AND (“cancer” OR “tumor” OR “neoplasm” OR “carcinoma” OR “malignancy” OR “leukemia” OR “lymphoma” OR “neuroblastoma” OR “retinoblastoma” OR “melanoma”) AND (“breastfeeding” OR “infant feeding” OR “infant nutrition”). In addition, we conducted manual retrieval of the relevant references.

### Inclusion and exclusion criteria

The eligible studies were included as follows: (1) study design of cohort or case-control, (2) clearly defining the outcome of interest as cancer of specific anatomical site [21–23], (3) reporting the relative risk (RR) or odds ratio (OR) and corresponding 95% CI to calculate the association between breastfeeding and cancer risk among children, or providing sufficient data to calculate them, (4) if study populations overlapped, we selected the one with larger sample size. The exclusion criteria were as follows: (1) systematic review or meta-analysis; (2) letter, meeting, or comment; (3) duplicate studies retrieved from various databases. Two reviewers (QS and XS) independently performed study review and inclusion, and discrepancies were solved by a third reviewer (DY).

### Data extraction and quality assessment

We extracted crucial information from the final studies retained, including first author, year of publication, data collection years, country or region, sample size, age in years, source of participants, cancer site, method of assessing breastfeeding, breastfeeding category, variables adjusted or matched, and corresponding risk estimates with 95% CIs.

Two researchers (QY and PZ) independently rated the quality of the included studies using the Newcastle-Ottawa Scale with scores ranging from 0 to 9 points [24]. This scale evaluates studies on the following aspects: (I) selection of cases and controls (4 scores); (II) comparability of cases and controls (2 scores); (III) ascertainment of exposure and non-response rate (3 scores). Studies with a quality score more than 7 points were considered as high quality. Two researchers (QS and QY) independently assessed the potential risk of bias using the risk of bias in nonrandomized studies of interventions (ROBINS-I) tool [25]. This tool encompasses seven domains: the presence of any confounding variables, selection bias, deviations from the exposure, misclassification of the exposure, missing data, measurement of outcomes, and selection of the reported results. In this approach, a study was categorized as “low risk,” “moderate risk,” “serious risk,” or “critical risk” of bias.

### Statistical analysis

All statistical analyses were performed using STATA version 14.0. The multivariate-adjusted risk estimates were selected if they were reported in the original article; otherwise, the unadjusted risk estimates were calculated using the original data. For the studies reported risk estimates relative to a reference category other than shortest breastfeeding duration, the risk estimates were recalculated using the shortest breastfeeding duration as reference by using the Orsini method [26]. When pooling the risk estimates, we regraded the shortest breastfeeding duration as the reference group (non/occasional breastfeeding), i.e., some defined never and some defined less than 1 month, 2 months, or 6 months.

In the analysis of breastfeeding versus non/occasional breastfeeding, if the corresponding estimate had not been presented in a study, estimates associated with different breastfeeding categories were synthesized into a single estimate. We also combined the risk estimates comparing the longest with the shortest breastfeeding duration among the studies with equal or greater than three different breastfeeding categories. The forest plot of the association between breastfeeding and the risk of childhood cancer was generated for breastfeeding and non/occasional breastfeeding and longest versus shortest breastfeeding duration, respectively.

A two-stage dose-response meta-analysis [27] was conducted to investigate the potential non-linear dose-response relationship between breastfeeding and the risk of childhood cancer. Briefly, a restricted cubic splines model with four knots at fixed percentiles, 5%, 35%, 65%, and 95%, of exposure level was used, which had negligible influence on the estimates. We assigned a null value to the lower bound of the reference group. The midpoint of the range was adopted to represent the category. When the category was open-ended, we assigned the midpoint of the upper open-ended category assuming that they had the same interval as the adjacent category.

The heterogeneity was evaluated by Q-statistic test and *I*-squared (*I*^2^) [28, 29]. The random-effects model was used to pool the effect estimates, as the approach can be used whether or not there is heterogeneity [30]. Subgroup analyses were performed by year of publication, geographic location, quality score, sample size, study design, and definition of reference category. Heterogeneity between strata by the above stratified factors was assessed by meta-regression analysis. Sensitivity analysis was performed to evaluate the effect of a particular study on the overall results by deleting one study at a time and combining the effect values of the remaining studies.

Funnel plot was generated, and the symmetry means no potential publication bias. Publication bias was assessed using Egger’s test [31] and Begg’s test [32].

## Results

### Systematic search

The flowchart of study selection is presented in Additional file 1: Figure S1. The primary search strategy for PubMed and Embase yielded 2905 and 2771 articles, and manual search from the reference lists of original studies or relevant reviews and meta-analyses on this topic yielded 67 additional articles. After removal of duplicates, 5545 articles were retrieved for assessment based on title and abstract, of which 116 articles were included for full text evaluation. After exclusion of 8 articles with insufficient data, 4 articles only reporting data on all cancers, 47 articles as meta, review, comment or meeting abstract, 2 articles conducted not among children, and 10 articles with overlapping data sets (Additional file 1: Table S2), a total of 45 articles with 475,579 participants were included for this meta-analysis [13, 14, 17, 18, 33–73].

### Characteristics of the included studies

The characteristics of selected studies are shown in Table 1. The total number of participants (from 140 to 410,147) varied widely across the included studies. All articles represented a range of geographical areas in Europe (*n* = 20), Asia (*n* = 8), and North America or Oceania (*n* = 17). There were 32 articles with population-based case-control design, 12 articles with hospital-based case-control design, and one article with cohort design. The median quality score of all included articles was 7, which resulted in 33 articles with a score of 7 or more and 12 articles with a score less than 7. Based on the ROBINS-I tool, 37 studies were considered at moderate risk of bias, and 8 studies were rated at serious risk of bias. Of the 45 articles, 33 studies provided the effect estimates for the association of breastfeeding on leukemia, 11 studies on lymphoma, 7 studies on brain tumors, 4 studies on neuroblastoma, 4 studies on soft-tissue sarcoma, 3 studies on nephroblastoma, 2 studies on retinoblastoma, and 2 studies on germ cell tumors. The details of included studies for the subsequent subgroup analysis are shown in Additional file 1: Table S3. Moreover, after excluding studies with the breastfeeding only as dichotomous variable, and no sufficient data of the number of cases and controls in each breastfeeding category, 23 studies were included for leukemia [13, 14, 18, 34–36, 38–43, 46, 47, 54, 56, 58, 61, 63, 64, 70–72], 6 studies for lymphoma [14, 33, 36, 40, 43, 46], and 6 studies for brain tumors [14, 40, 44, 51, 64, 67] in dose-response meta-analysis.

**Table 1:** Characteristics of studies included in the meta-analysis

First author, year, country	Data collection years	Design	No. of cases/controls^1^	Age (years)	Source of cases	Source of controls	Cancer type	Breastfeeding categories (months)	Method of assessing breastfeeding	Variables matched or adjusted	NOS	Risk of bias
Davis, 1988, USA [33]	1976–1983	PC-CS	201/181	1.5–15	Colorado Central Cancer Registry	Population	Leukemia (*N* = 63); brain tumors (*N* = 38); lymphoma (*N* = 26); STS (*N* = 15); other cancers (*N* = 59)	Never/≤ 6/> 6 (lymphoma); ≤ 6/> 6 (leukemia, brain tumors, STS)	Interview of parents	Matched: age (±3 years), sex, telephone exchange. Adjusted: none.	7	Moderate
Magnani, 1988, Italy [34]	1981–1984	HC-CS	182/307	NA	The main pediatric hospital of Turin	Hospital patients	Leukemia (*N* = 163); NHL (*N* = 19)	Never/1–6/> 7	Interview of parents	Matched: none. Adjusted: none.	5	Serious
Van Duijn, 1988, Netherlands [35]	1973–1979	PC-CS	492/480	1–15	The national registry of the Dutch Childhood Leukemia Study Group	Population	ALL (*N* = 492)	Never/≤ 6/> 6	Questionnaires mailed to parents	Matched: age (±3 years), sex. Adjusted: age, sex, birth order, social class, maternal education, and maternal factors (age, smoking, alcohol use in pregnancy).	7	Moderate
Shu, 1995, China [36]	1981–1991	PC-CS	241/241	1–15	Shanghai Cancer Registry	Population	Lymphoma (*N* = 82); leukemia (*N* = 159)	Never/1–6/> 6	Interview of parents	Matched: sex, year of birth. Adjusted: maternal age at birth, birthweight, maternal working status and occupational exposure to chemicals during infancy.	8	Moderate
Shu, 1995, USA and Canada [17]	1982–1989	PC-CS	105/639	0–15	The Children’s Cancer Group (CCG), 1982–1989	Population	Malignant germ cell tumors (*N* = 105)	Never/1–6/7–12/> 12	Self-administered questionnaire to mother	Matched: none. Adjusted: age, gender, gestational age, number of livebirths, maternal education, and smoking during pregnancy.	7	Moderate
Petridou, 1997, Greece [37]	1993–1994	HC-CS	153/300	0–14	A nationwide network of childhood hematologists/oncologists	Hospital patients	Leukemia (*N* = 153)	Ever vs. never	Interview of guardians	Matched: place of residence, gender and age. Adjusted: sex, age, place of residence, sociodemographic, lifestyle, environmental and biomedical variables.	7	Serious
Schüz, 1999, Germany [38]	1992–1997	PC-CS	1001/1001	0–14	German Children’s Cancer Registry (GCCR)	Population	Leukemia (*N* = 1001)	≤ 1/2–6/> 6	Questionnaire and telephone interview	Matched: gender, date of birth and district. Adjusted: socioeconomic status (SES).	7	Moderate
Shu, 1999, USA and Canada [39]	1989–1993	PC-CS	2200/2418	1–17	The Children’s Cancer Group (CCG), 1989–1993	Population	Leukemia (*N* = 2200)	Never/1–3/4–6/7–9/10–12/> 12; Ever vs. never	Interview of mothers	Matched: age, geographic location and ethnicity. Adjusted: maternal race, maternal education, and family annual income.	8	Moderate
Smulevich, 1999, Russia [40]	1986–1988	PC-CS	593/1181	0–14	Russian Cancer Research Center RAMS	Population	Lymphoma (*N* = 117); leukemia (*N* = 199); brain tumors (*N* = 57); neuroblastoma(*N* = 42); nephroblastoma (*N* = 48); STS (*N* = 53); other cancers (*N* = 77)	< 1/1–2/3–4/5–6/7–12/> 12 (leukemia, STS, neuroblastoma, nephroblastoma); ≤ 2/3–4/5–6/7–12/> 12 (lymphoma, brain tumors)	Interview of mothers	Matched: age, gender and residence. Adjusted: none.	7	Moderate
Dockerty, 1999, New Zealand [41]	1991–1995	PC-CS	97/303	0–14	National databases including the New Zealand Cancer Registry	Population	ALL (*N* = 97)	Never/≤6/> 6–12/> 12; Ever vs. never	Interview of mothers	Matched: age and sex.Adjusted: age, sex, child’s social class, mother’s education, household crowding, delay from reference date to interview.	8	Moderate
Infante-Rivard, 2000, Canada [42]	1980–1993	PC-CS	491/491	0–9	Tertiary care centers	Population	ALL (*N* = 491)	Never/≤ 3/> 3	Interview of mothers	Matched: age, sex, and region of residence at the time of diagnosis.Adjusted: none.	8	Moderate
UKCCS Investigators, 2001, UK [43]	1991–1996	PC-CS	3500/6964	1–14	UKCCS	Population	Lymphoma (*N* = 342); leukemia (*N* = 1637); other cancers (*N* = 1521)	Never/< 1/1–6/≥ 7; Ever vs. never	Interview of mothers	Matched: month and year of birth, sex and region of residence at diagnosis.Adjusted: age at diagnosis, sex, region, birth order and deprivation index.	7	Moderate
Hardell, 2001, Sweden [14]	1993–1996	PC-CS	835/860	0–14	Swedish Cancer Register	Population	Leukemia (*N* = 235); lymphoma (*N* = 99); brain tumors (*N* = 264); neuroblastoma (*N* = 34); retinoblastoma (*N* = 22); germ cell tumors (*N* = 21); STS(*N* = 37); other cancers (*N* = 123)	0– < 1/1– < 6/≥ 6;≥ 1 vs. 0- < 1	Routine child health records	Matched: age and sex.Adjusted: none.	8	Moderate
Schüz, 2001, Germany [44]	1988–1994	PC-CS	465/2442	0–14	German Children’s Cancer Registry (GCCR)	Population	Brain tumors (*N* = 281); other cancers (*N* = 184)	≤ 1/2–6/> 6	Questionnaire and telephone interview	Matched: gender and date of birth within 1 year.Adjusted: degree of urbanization and socioeconomic status.	7	Moderate
Daniels, 2002, USA and Canada [45]	1992–1994	PC-CS	393/376	0.5–19	The Children’s Oncology Group in the United States and Canada, 1992–1994	Population	Neuroblastoma (*N* = 393)	Never/0–3/4–6/7–9/9–12/≥ 13; Ever vs. never	Interview of mothers	Matched: date of birth.Adjusted: income and age.	6	Moderate
Murray, 2002, UK [46]	1975–1986	Historical cohort	178/409969	0–16	The Northern Ireland Cancer Registry	Population	ALL (*N* = 178)	Ever vs. never	Child health records	Matched: none.Adjusted: none.	6	Serious
Perrillat, 2002, France [47]	1995–1999	HC-CS	247/237	2–15	The hospitals of Lille, Lyon, Nancy and Paris	Hospital patients	Leukemia (*N* = 247)	Never/< 3/3–5/6–11/≥ 12; Ever vs. never	Interview of mothers	Matched: age, gender, hospital, hospital catchment area and ethnic origin.Adjusted: age, gender, hospital, ethnic origin, maternal educational level and parental socio professional category, birth weight, length of pregnancy and number of previous pregnancies	8	Moderate
Lancashire, 2003, UK [13]	1972–1981	PC-CS	3376/3376	1–14	The Oxford Survey of Childhood Cancers (OSCC)	Population	Leukemia (*N* = 1342); other cancers (*N* = 2034)	Never/< 1/1–6/≥ 7; Ever vs. never	Interview of parents	Matched: sex and date of birth.Adjusted: sex, age at death, father’s occupational social class, sibship position, maternal age at child’s birth.	7	Moderate
Jourdan-Da, 2004, France [48]	1995–1998	PC-CS	452/530	1–15	The National Registry of Childhood Leukemia and Lymphoma	Population	Leukemia (*N* = 452)	Never/< 3/3–6/> 6; Ever vs. never	Self-administered questionnaire to mother	Matched: age, sex and region.Adjusted: gender, age at diagnosis, region of residence at diagnosis.	7	Moderate
Altinkaynak, 2006, Turkey [49]	1990–2000	PC-CS	137/146	1–16	The Ataturk University Medicine Faculty	Population	Leukemia (*N* = 87); lymphoma(*N* = 50)	0–6 vs. > 6	Interview of mothers	Matched: sex and age.Adjusted: none.	7	Moderate
Saddlemire, 2006, USA and Canada [50]	1999–2002	PC-CS	461/443	0.5–16	The Children’s Oncology Group in the United States and Canada, 1999–2002	Population	Wilms tumor (*N* = 461)	Never/0–3/4–6/7–9/10–12/≥ 13; Ever vs. never	Telephone interviews of mothers	Matched: age strata and geographic region of residence.Adjusted: child’s age at reference date, geographic region of residence, household income and mother’s education.	7	Moderate
Shaw, 2006, Canada [51]	1980–1993;1995–1999	PC-CS	272/272	0–14	Tertiary care centers in the province of Quebec	Population	Brain tumors (*N* = 272)	Never/≤ 8 weeks/9-24 weeks/> 24 weeks; Ever vs. never	Interview of parents	Matched: sex and age at diagnosis.Adjusted: mother’s education level.	6	Moderate
Petridou, 2006, Greece [52]	1996–2002	HC-CS	71/71	0–14	A nationwide network of childhood hematologists/oncologists	Hospital patients	HD (*N* = 71)	Ever vs. never	Interview of guardians	Matched: gender and age (±6 months).Adjusted: demographic, socioeconomic, anthropometric and perinatal variables.	7	Serious
Bener, 2008, United Arab Emirates [53]	1983–2004	HC-CS	169/169	0–15	Tawam Hospital, Al Ain	Hospital (healthy subjects)	ALL (*N* = 103); HD (*N* = 32); NHL (*N* = 34)	0–6 vs. > 6	Telephone interviews with mothers	Matched: age and sex.Adjusted: none.	6	Moderate
MacArthur, 2008, Canada [54]	1990–1994	PC-CS	399/399	0–14	Pediatric oncology treatment centers and cancer registries	Population	Leukemia (*N* = 399)	Never/0–3/4–6/7–12/> 12	Interview of parents or guardians	Matched: age, gender, and area.Adjusted: maternal age at birth, maternal education, annual household income, ethnicity, and number of residences since birth.	6	Moderate
Flores-Lujano, 2008, Mexico [55]	1998–2006	HC-CS	57/218	0–19	The institutions that treat children with cancer in Mexico	Children with Down syndrome	Leukemia (*N* = 57)	Never/≤ 6/≥ 7; Ever vs. never	Interview of parents	Matched: none.Adjusted: sex, weight at birth < 2500 g, age of child, firstborn child, low standard of living and cardiovascular diseases.	6	Moderate
Rudant, 2010, France [56]	2003–2004	PC-CS	720/1494	1–14	ESCALE, 2003–2004	Population	ALL (*N* = 634); AML (*N* = 86)	Never/≤ 2/3–5/6–11/≥ 12; Ever vs. never	Telephone interview of mothers	Matched: age and gender.Adjusted: age, gender, parental professional category, degree of urbanization, and maternal age at child’s birth.	8	Moderate
Rudant, 2011, France [57]	2003–2004	PC-CS	291/2149	2–14	ESCALE, 2003–2004	Population	HD (*N* = 127); NHL (*N* = 164)	Never/< 6/≥ 6	Telephone interview of mothers	Matched: age and gender.Adjusted: age, gender, parental professional category, degree of urbanization, maternal age at child birth and housing, for contact with animals.	8	Moderate
Waly, 2011, Sultanate of Oman [58]	2008–2010	HC-CS	70/70	NA^2^	The Sultan Qaboos University Hospital	Relatives of the cases	ALL (*N* = 70)	< 6/6–12/12–24/ > 24	Interview of mothers	Matched: none.Adjusted: none.	6	Serious
Urayama, 2012, USA [59]	1995–2008	PC-CS	507/762	1–14	The Northern California Childhood Leukemia Study	Population	ALL (*N* = 507)	Ever vs. never	Questionnaire to parents or guardians	Matched: date of birth, sex, Hispanic status, and maternal race.Adjusted: age, sex, maternal age, maternal education, annual household income and race/ethnicity.	8	Moderate
Lupo, 2014, USA [60]	1982–1988	PC-CS	319/317	0–20	Intergroup Rhabdomyosarcoma Study Group (IRSG)	Population	Rhabdomyosarcoma (*N* = 319)	Never/< 6/6–12/≥ 12; Ever vs. never	Telephone interview to mothers	Matched: race, sex, and age.Adjusted: age, race, sex, household income, maternal, and paternal education.	7	Moderate
Mattioli, 2014, Italy [61]	1998–2003	PC-CS	82/1040	0–10	Childhood acute leukemia in 14 Italian Regions	Population	AnLL (*N* = 82)	Never/1–3/4–6/> 6	Interview of parents	Matched: date of birth, sex and residence area.Adjusted: none.	6	Serious
Schraw, 2014, USA [62]	1997–2011	PC-CS	142/284	0–14	The Texas Children’s Cancer Center	Healthy subjects	ALL (*N* = 142)	Ever vs. never	Questionnaire to mothers	Matched: age, sex, race, and ethnicity.Adjusted: none.	7	Moderate
Ajrouche, 2015, France [63]	2010–2011	PC-CS	617/1225	1–14	ESTELLE, 2010–2011	Population	ALL (*N* = 617)	Never/< 6/≥ 6; Ever vs. never	Telephone interview of mothers	Matched: age and gender.Adjusted: age, gender, parental professional category, maternal age, and degree of urbanization.	8	Moderate
Greenop, 2015, Australia [64]	2003–2006;2005–2010	PC-CS	592/1389	0–14	Aus-ALL and Aus-CBT	Population	ALL (*N* = 314); brain tumors (*N* = 278)	Never/< 3/3- < 6/≥ 6; Ever vs. never	Questionnaire to mothers	Matched: age, sex, and state of residence.Adjusted: Matched variables, maternal education, maternal age at birth, birth order, proportion of optimum birth weight.	7	Moderate
Heck, 2015, USA and Canada [65]	2006–2011	HC-CS	243/134	0–15	Wills Eye Hospital in Philadelphia, or the Children’s Oncology Group	Friends or unrelated children of case family	Retinoblastoma (*N* = 243)	< 1/1–6/7–11/≥ 12; Ever vs. never	Telephone interview of parents	Matched: age.Adjusted: child’s age at interview, mother’s race/ethnicity, mother’s educational attainment, and household income.	7	Moderate
Amitay, 2016, Israeli [66]	2005–2013	PC-CS	178/357	1–18	The hematologic departments of five large Israeli hospitals	Population	Leukemia (*N* = 117); lymphoma (*N* = 61)	≤ 6 vs. > 6	Interview of mothers	Matched: age, gender, and spoken language.Adjusted: none.	7	Moderate
Rios, 2016, France [67]	2003–2004;2010–2011	PC-CS	269/1589	0.5–6	ESCALE, 2003–2004; ESTELLE, 2010–2011	Population	Neuroblastoma (*N* = 269)	Never/< 3/3–5/≥ 6; Ever vs. never	Telephone interview of mothers	Matched: age and gender.Adjusted: age and sex, birth order, maternal age, urban status of the area of residence and study.	8	Moderate
Bailey, 2017, France [68]	2003–2004;2010–2011	PC-CS	469/2719	1–14	ESCALE, 2003–2004; ESTELLE, 2010–2011	Population	Brain tumors (*N* = 469)	Never/< 3/3- < 6/≥ 6; Ever vs. never	Telephone interview of mothers	Matched: age and gender.Adjusted: age and gender.	8	Moderate
Schraw, 2017, USA [69]	1997–2012	PC-CS	171/342	0–14	Texas Children’s Cancer Center in Houston	Population	ALL (*N* = 171)	Ever vs. never	Questionnaire to parents	Matched: sex, ethnicity, and age.Adjusted: none.	7	Moderate
Gao, 2018, China [70]	2008–2017	HC-CS	958/785	0–14	The Children’s Hospital of Zhejiang University	Hospital patients	Leukemia (*N* = 958)	Never/1–3/4–6/7–9/10–12/≥ 13	Medical records	Matched: none.Adjusted: family history of cancer, the family history of neoplasm of the lymphatic/hematopoietic systems, history of bedroom decoration, smoking during pregnancy, the history of using birth control pills before pregnancy, abortion history, Down’s syndrome and parents use of hair dye.	8	Moderate
Jiménez-Hernández, 2018, Mexico [18]	2010–2015	HC-CS	1455/1455	0–17.6	Private and public hospitals in Mexico City	Hospital patients	Leukemia (*N* = 1455)	Never/0–3.9/4–6.9/7–12.9/> 13	Interview of mothers	Matched: none.Adjusted: none.	5	Serious
Lingappa, 2018, India [71]	2015	HC-CS	120/120	1–15	Kidwai Institute of Oncology	the elder sibling of the cases	Leukemia (*N* = 120)	Never/< 4/4–6/6–9/ 9–12/13–18/ 19–24/> 24;Ever vs. never	Interview of mothers	Matched: parental education, socioeconomic status, and the region of residence.Adjusted: none.	5	Moderate
Rafieemehr, 2019, Iran [72]	2015–2018	HC-CS	125/130	1–12	Be’sat Hospital of Hamadan University of Medical Sciences	Hospital (healthy subjects)	ALL (*N* = 125)	Ever vs. never; 1–3/4–6/7–9/ 10–12/≥ 24	Interview of parents	Matched: age, sex, gender, and residence location.Adjusted: none.	6	Serious
Bauer, 2020, France [73]	2015–2018	PC-CS	112/996	0.5–11	ESTELLE, 2010–2011	Population	Wilms tumor (*N* = 112)	Never/ < 3/3–5/≥ 6; Ever vs. never	Telephone interview of mothers	Matched: age and gender. Adjusted: age, gender, birth order, and maternal age.	7	Moderate

Abbreviations: *PC-CS* population-based case-control study, *HC-CS* hospital-based case-control study, *CNS* central nervous system, *ALL* acute lymphoblastic leukemia, *AML* acute myeloid leukemia, *AnLL* acute nonlymphoblastic leukemia, *HD* Hodgkin’s disease, *NHL* non-Hodgkin lymphoma, *NA* not applicable, *UKCCS* United Kingdom Childhood Cancer Study, *Aus-ALL* The Australian Study of the Causes of Acute Lymphoblastic Leukemia, *Aus-CBT* Australian Study of Childhood Brain Tumors, *STS* Soft-tissue sarcoma, *ESCALE and ESTELLE* The ESCALE and ESTELLE studies were two nationwide population-based studies in France, designed to investigate the role of environmental, infectious, and genetic factors of childhood cancers

^1^No. of cases/controls in breastfeeding analysis; ^2^Mean ± SD of age in cases and controls were 12.1 ± 2.72 and 13.5 ± 2.85, respectively, while age range had not been reported

#### Leukemia

##### Summary of main findings

**Fig. 1:**
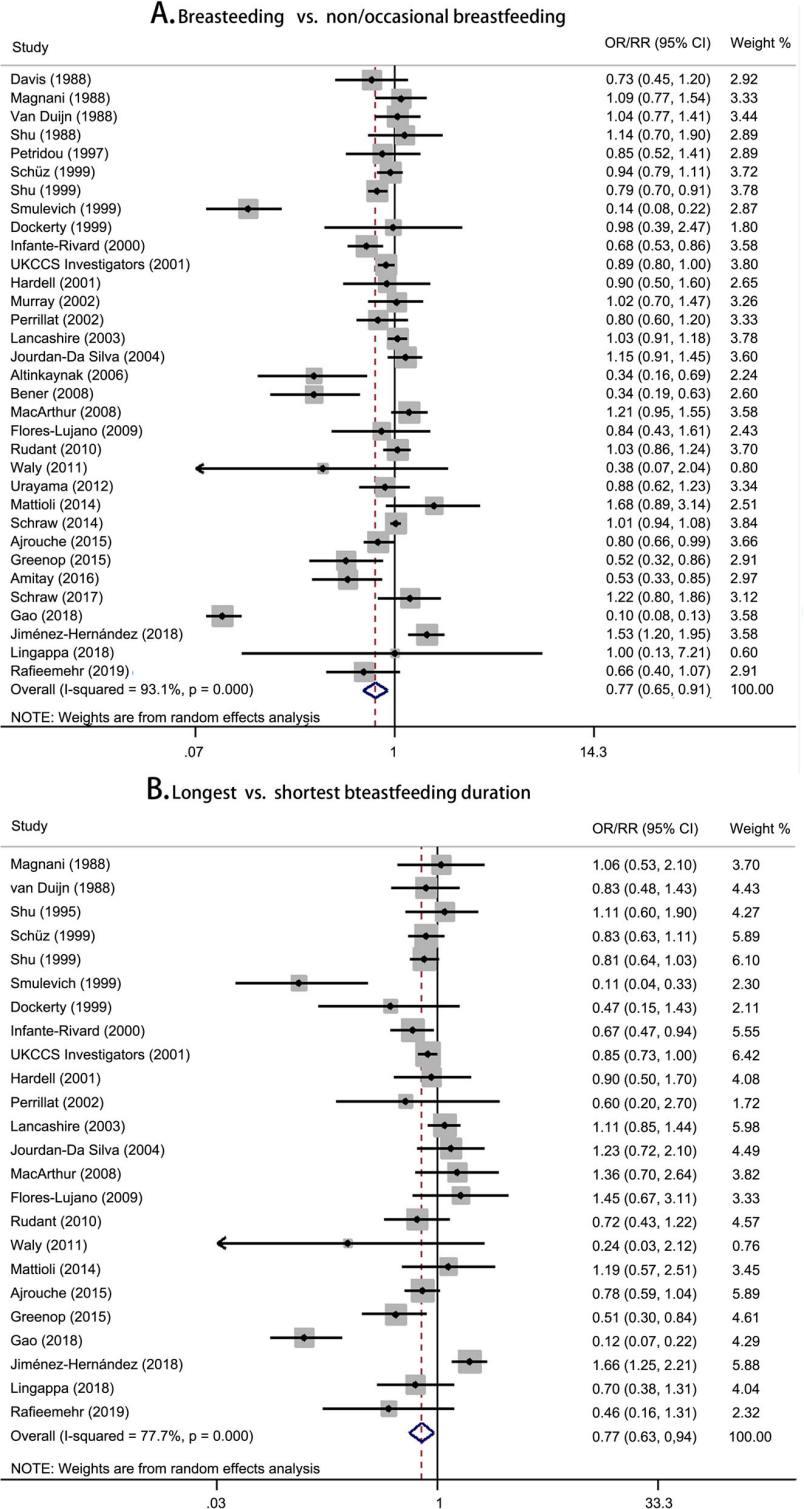
Forest plots for pooled risk estimates and the corresponding 95% confidence intervals (CIs) of childhood leukemia risk for **a** breastfeeding vs. non/occasional breastfeeding and **b** longest vs. shortest breastfeeding duration

**Fig. 2:**
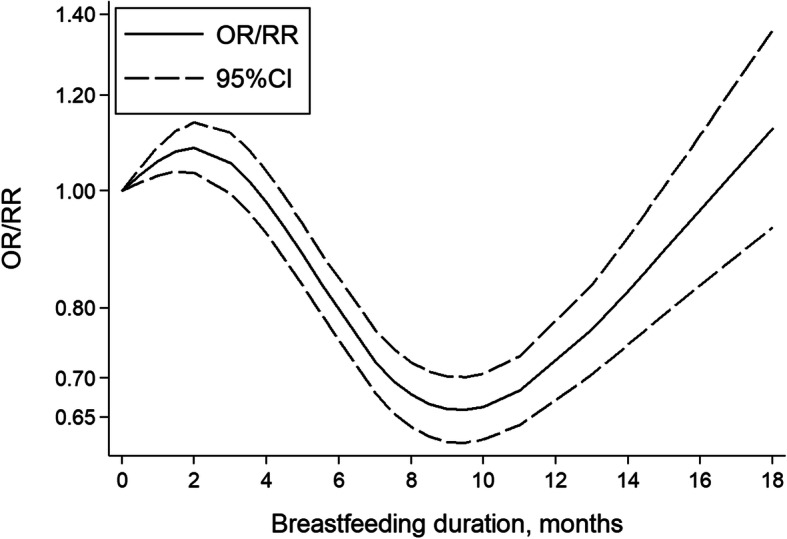
Risk estimates (solid line) and the corresponding 95% CIs (dash lines) for the dose-response relationship between breastfeeding and the risk of childhood leukemia

The summary risk estimates of the risk of childhood leukemia were 0.77 (95% CI, 0.65–0.91) for breastfeeding versus non/occasional breastfeeding and 0.77 (95% CI, 0.63–0.94) for longest versus shortest breastfeeding duration (Fig. 1). A significant non-linear dose-response relationship between breastfeeding and the risk of childhood leukemia was found (*P* < 0.001 for non-linearity; Fig. 2). The overall dose-response relationship approximated to a U-shaped curve. Compared with never breastfeeding, the risk of leukemia was statistically significant at a duration of 4.4–15.0 months. The most protective effect (OR = 0.66, 95% CI 0.62–0.70) was observed at a duration of 9.6 months. Specifically, the average breastfeeding duration of 6 months and 12 months reduced 20% (95% CI 15%–25%) and 27% (95% CI 22%–33%) of the risk of childhood leukemia, respectively. Begg’s funnel plot was presented in Additional file 1: Figure S2. The shape of the funnel plot revealed no asymmetric distribution with a *P* value of 0.075 by Begg’s test and 0.173 by Egger’s test.

##### Subgroup analysis and meta-regression analysis

In the subgroup analysis for the comparison of breastfeeding versus non/occasional breastfeeding, there were significant differences in the heterogeneity between strata by geographic location (*P* in meta-regression = 0.016) and definition of reference category (*P* in meta-regression = 0.025) (Table 2). Protective effect of breastfeeding on the risk of childhood leukemia cancer was found in population from Asia (OR, 0.43; 95% CI, 0.19–0.98), but not Europe and North American or Oceania. The inverse association was more pronounced among the studies with defining occasional breastfeeding as reference group (OR, 0.47; 95% CI, 0.28–0.80), especially for the studies using breastfeeding less than 6 months as reference group (OR = 0.49, 95% CI 0.35–0.67). Subgroup analysis of publication year showed similar results with main analysis, and studies with small and larger sample size showed slightly different results. Significant associations were found in studies with high-quality score (OR, 0.72; 95% CI, 0.59–0.87) and population-based case-control studies (OR, 0.86; 95% CI, 0.76–0.96).

**Table 2:** Subgroup analysis of association between breastfeeding and childhood leukemia risk for the comparison of breastfeeding versus non/occasional breastfeeding

	No. of studies	No. of cases	OR (95%CI)	*P* value	*I*^2^ (%)	*P* value for heterogeneity	*P* in meta-regression
Cancer type							0.629
AML (A.1)	11	1241	0.96 (0.79 ~ 1.18)	0.723	38.9	< 0.001	
ALL (A.2)	27	11,554	0.92 (0.84 ~ 1.00)	0.057	66.4	< 0.001	0.500
B cell ALL (A.3)	6	3974	0.91 (0.81 ~ 1.01)	0.070	47.3	0.350	
T cell ALL (A.4)	4	472	1.00 (0.79 ~ 1.26)	0.996	8.6	0.091	
Year of publication							0.921
In and before 2000 (A.5)	10	5018	0.76 (0.59 ~ 0.97)	0.030	84.7	< 0.001	
After 2000 (A.6)	23	10,135	0.77 (0.62 ~ 0.97)	0.023	94.5	< 0.001	
Geographic location							0.016
Europe (A.7)	14	7518	0.89 (0.77 ~ 1.04)	0.147	81.2	< 0.001	
Asia (A.8)	8	1739	0.43 (0.19 ~ 0.98)	0.044	94.1	< 0.001	
North America or Oceania (A.9)	11	5896	0.93 (0.79 ~ 1.09)	0.386	77.6	< 0.001	0.259
North America only (A.10)	8	3285	1.01 (0.84 ~ 1.21)	0.960	74.0	< 0.001	
Oceania only (A.11)	2	411	0.63 (0.36 ~ 1.12)	0.113	29.0	0.235	
Both^1^ (A.12)	1	2200	0.79 (0.69 ~ 0.90)	< 0.001	NA	NA	
Study quality score							0.280
< 7 (A.13)	10	2752	0.97 (0.74 ~ 1.28)	0.827	70.5	< 0.001	
≥ 7 (A.14)	23	12,401	0.72 (0.59 ~ 0.87)	0.001	94.7	< 0.001	
Sample size							0.787
≤ 500 (A.15)	15	1938	0.77 (0.64 ~ 0.93)	0.007	59.3	0.002	
> 500 (A.16)	18	13,215	0.78 (0.61 ~ 1.01)	0.061	95.9	< 0.001	
Study design							0.490
PC-CS (A.17)	22	11,524	0.86 (0.76 ~ 0.96)	0.009	81.0	< 0.001	
HC-CS (A.18)	10	3451	0.61 (0.29 ~ 1.32)	0.214	96.8	< 0.001	
Cohort study (A.19)	1	178	1.02 (0.70 ~ 1.48)	0.917	NA	NA	
Definition of reference category							0.024
Never breastfeeding (A.20)	25	13,278	0.87 (0.72 ~ 1.04)	0.132	93.8	< 0.001	
Occasional breastfeeding (A.21)	8	1875	0.47 (0.28 ~ 0.80)	0.005	88.8	< 0.001	0.872
≤ 6 months (A.22)	5	440	0.49 (0.35 ~ 0.67)	< 0.001	21.7	0.276	
≤ 1 month (A.23)	3	1435	0.50 (0.15 ~ 1.60)	0.240	95.9	< 0.001	

Abbreviations: *PC-CS* population-based case-control study; *HC-CS* hospital-based case-control study; *NA* not applicable

^1^One study was conducted in the United States, Canada and Australia

We also conducted subgroup analysis stratified by histologic type and found that breastfeeding was associated with a decreased risk for ALL at borderline statistical significance (OR = 0.92, 95% CI 0.84–1.00), but not associated with acute myeloid leukemia (AML) risk (Table 2). Moreover, all subgroup analysis of ALL were consistent with those of childhood leukemia (Additional file 1: Table S4). Further stratified by immunophenotype in ALL, the results showed a weak evidence of borderline statistical significance that breastfeeding was associated with a small reduction in B cell ALL risk, but no evidence of the association between breastfeeding and risk of T cell ALL (Table 2). The forest plots of subgroup analysis are shown in Additional file 1: Figure S3.

##### Sensitivity analysis

The one-study-removed analysis showed that exclusion of each study did not significantly change the results (Additional file 1: Figure S4). Considering that the incidence of childhood leukemia varies with age, which may have an influence on the risk estimate, we performed the repeated analyses by only including the group of studies among children aged 0–14 years old. Consequently, we found that there was no significant effect on the pooled risk estimates (Additional file 1: Figure S5).

#### Lymphoma

##### Summary of main findings

**Fig. 3:**
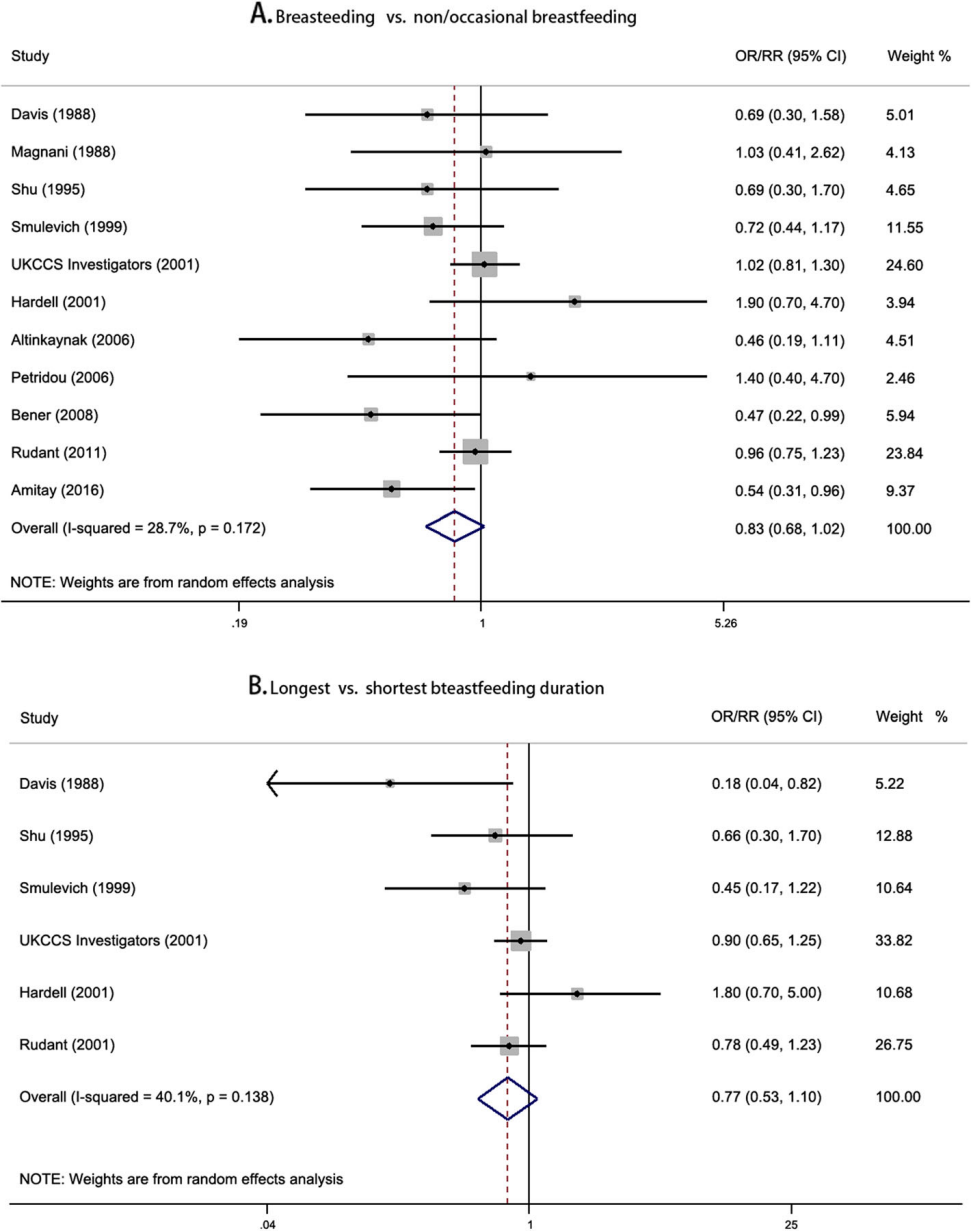
Forest plots for pooled risk estimates and the corresponding 95% confidence intervals (CIs) of childhood lymphoma risk for **a** breastfeeding vs. non/occasional breastfeeding and **b** longest vs. shortest breastfeeding duration

There was no suggestive evidence of the association between breastfeeding and risk of childhood lymphoma, with the pooled risk estimates of 0.83 (95% CI, 0.68–1.02) and 0.77 (95% CI, 0.53–1.10) for the comparison of breastfeeding versus non/occasional breastfeeding and longest versus shortest breastfeeding duration, respectively (Fig. 3). The non-linear dose-response relationship curve showed that the association between breastfeeding and the risk of childhood lymphoma was significant in a narrow range of breastfeeding duration (*P* = 0.046 for non-linearity; Additional file 1: Figure S6). The funnel plot showed symmetry distribution with a *P* value of 0.533 by Begg’s test and 0.267 by Egger’s test (Additional file 1: Figure S7).

##### Subgroup analysis and meta-regression analysis

Heterogeneity between strata by geographic location (*P* = 0.041) and definition of reference group (*P* = 0.044) was identified by meta-regression analysis (Table 3). Only the studies conducted in Asia indicated a significant association between breastfeeding and risk of childhood lymphoma (OR = 0.53, 95% CI 0.37–0.76). In the subgroup of studies with defining occasional breastfeeding as reference category, the association between breastfeeding and risk of childhood lymphoma was significant (OR = 0.66, 95% CI 0.44–0.99). Comparing with the breastfeeding duration of less than 6 months, the pooled risk estimate of prolong breastfeeding for the risk of lymphoma was 0.50 (95% CI 0.34–0.75). Moreover, there were no significant associations of breastfeeding with both Hodgkin’s lymphoma and non-Hodgkin’s lymphoma (Table 3). The forest plots of stratified analysis are shown in Additional file 1: Figure S8.

**Table 3:** Subgroup analysis of association between breastfeeding and childhood lymphoma risk for the comparison of breastfeeding versus non/occasional breastfeeding

	No. of studies	No. of cases	OR (95%CI)	*P* value	*I*^2^ (%)	*P* value for heterogeneity	*P* in meta- regression
Cancer type							0.875
Hodgkin’s disease (B.1)	9	458	0.86 (0.63 ~ 1.18)	0.341	25.8	0.215	
Non-Hodgkin’s lymphoma (B.2)	9	686	0.88 (0.67 ~ 1.15)	0.343	26.9	0.205	
Year of publication							0.583
In and before 2000 (B.3)	4	244	0.75 (0.53 ~ 1.06)	0.103	0.0	0.908	
After 2000 (B.4)	7	980	0.84 (0.63 ~ 1.12)	0.231	51.5	0.054	
Geographic location							0.041
Europe (B.5)	6	939	0.98 (0.84 ~ 1.15)	0.826	0.0	0.571	
Asia (B.6)	4	259	0.53 (0.37 ~ 0.76)	0.001	0.0	0.906	
North America (B.7)	1	26	0.69 (0.30 ~ 1.58)	0.381	NA	NA	
Study quality score							0.436
< 7 (B.8)	2	85	0.66 (0.31 ~ 1.42)	0.290	39.7	0.198	
≥ 7 (B.9)	9	1139	0.86 (0.70 ~ 1.06)	0.152	28.0	0.196	
Sample size							0.578
≤ 200 (B.10)	5	368	0.77 (0.44 ~ 1.35)	0.366	45.5	0.119	
> 200 (B.11)	6	856	0.89 (0.75 ~ 1.06)	0.203	12.7	0.334	
Study design							0.743
PC-CS (B.12)	8	1068	0.85 (0.68 ~ 1.05)	0.125	34.1	0.156	
HC-CS (B.13)	3	156	0.78 (0.41 ~ 1.51)	0.461	31.4	0.233	
Definition of reference category							0.044
Never breastfeeding (B.14)	6	831	0.97 (0.83 ~ 1.14)	0.732	0.0	0.879	
Occasional breastfeeding (B.15)	5	393	0.66 (0.44 ~ 0.99)	0.042	40.8	0.149	0.170
≤ 2 months (B.16)	2	216	1.07 (0.42 ~ 2.72)	0.888	68.3	0.076	
≤ 6 months (B.17)	3	177	0.50 (0.34 ~ 0.75)	0.001	0.0	0.937	

Abbreviations: *PC-CS* population-based case-control study; *HC-CS* hospital-based case-control study; *NA* not applicable

##### Sensitivity analysis

Omitting the studies of UKCCS Investigators et al. [43] and Hardell et al. [14] modified the pooled risk estimates in sensitivity analysis, suggesting the results may be unstable (Additional file 1: Figure S9). Sensitivity analysis by only including the group of studies among children aged 0–14 years old showed the pooled risk estimate of 0.98 (95% CI 0.84–1.15; Additional file 1: Figure S10).

#### Brain tumors

**Fig. 4:**
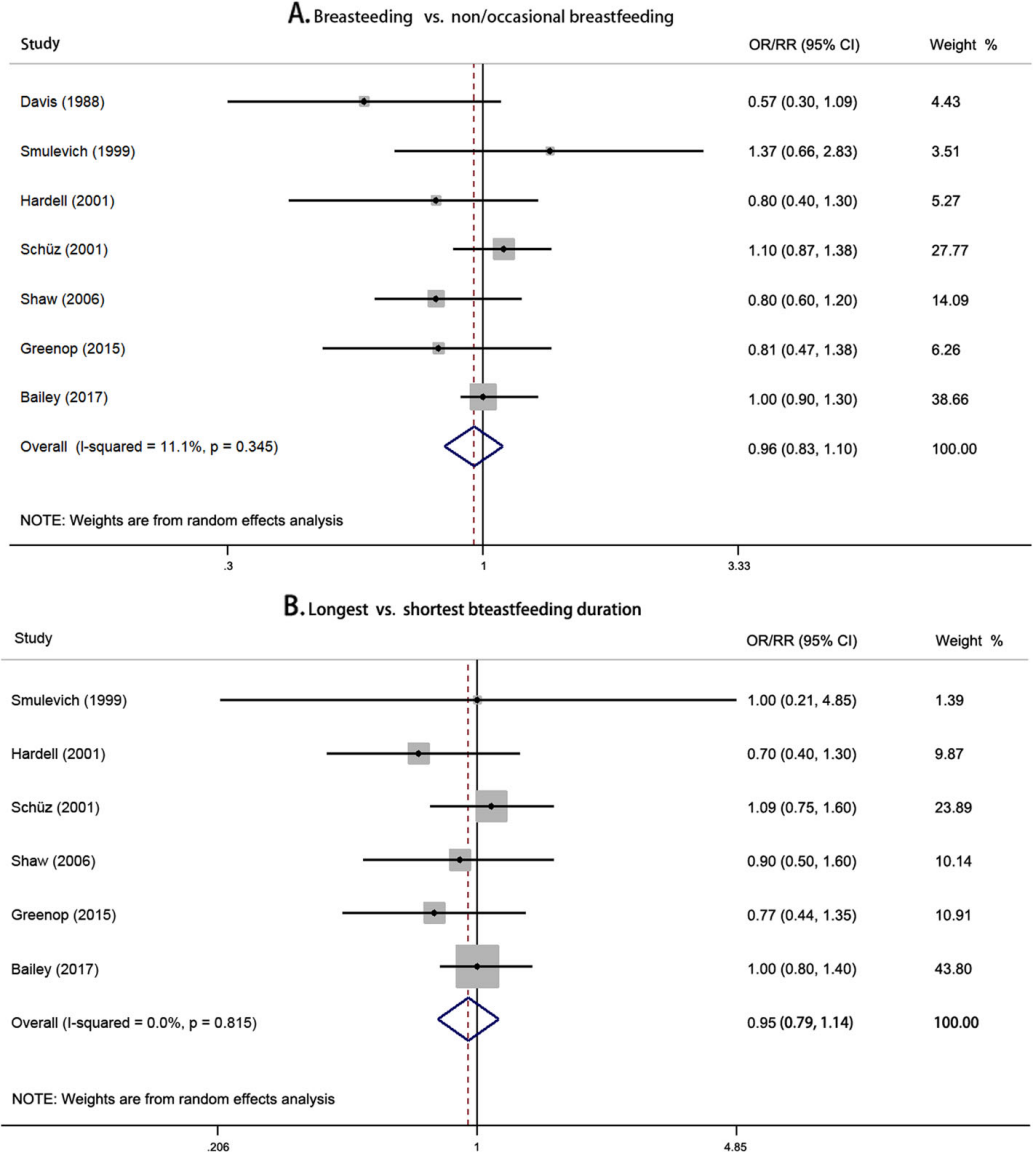
Forest plots for pooled risk estimates and the corresponding 95% confidence intervals (CIs) of the risk of childhood brain tumors for **a** breastfeeding vs. non/occasional breastfeeding and **b** longest vs. shortest breastfeeding duration

No significant association between breastfeeding and risk of childhood brain tumors was found (Fig. 4). Non-linear dose-response relationship was also not observed (*P* = 0.776 for non-linearity). There was no sign of asymmetry with a *P* value of 0.764 by Begg’s test and 0.261 by Egger’s test (Additional file 1: Figure S11). All subgroups showed no significant association between breastfeeding and risk of childhood brain tumors (Table 4). The corresponding forest plots of subgroup analysis are presented in Additional file 1: Figure S12. Sensitivity analysis excluding each study did not substantially change the results (Additional file 1: Figure S13). Sensitivity analysis by excluding the studies considering upper age limits equal and higher than 15 years yielded similar result with the pooled risk estimate of 0.99 (95% CI 0.87–1.12; Additional file 1: Figure S14).

**Table 4:** Subgroup analysis of association between breastfeeding and risk of childhood brain tumors for the comparison of breastfeeding versus non/occasional breastfeeding

	No. of studies	No. of cases	OR (95%CI)	*P* value	*I*^2^ (%)	*P* value for heterogeneity	*P* in meta-regression
Cancer type							0.396
Ependymoma (C.1)	4	408	1.02 (0.65 ~ 1.60)	0.931	22.3	0.277	
Astrocytoma (C.2)	4	475	0.91 (0.71 ~ 1.15)	0.411	0.0	0.589	
Medulloblastoma (C.3)	2	148	0.89 (0.42 ~ 1.92)	0.770	53.4	0.143	
Others (C.4)	3	446	1.13 (0.83 ~ 1.55)	0.446	22.1	0.274	
Year of publication							0.665
In and before 2005 (C.5)	4	640	0.95 (0.69 ~ 1.30)	0.751	37.8	0.186	
After 2005 (C.6)	3	1019	0.94 (0.80 ~ 1.10)	0.431	0.0	0.458	
Geographic location							0.296
North America (C.7)	2	310	0.74 (0.55 ~ 1.01)	0.055	0.0	0.364	
Europe (C.8)	4	1071	1.03 (0.90 ~ 1.19)	0.639	0.0	0.636	
Oceania (C.9)	1	278	0.81 (0.47 ~ 1.39)	0.443	NA	NA	
Study quality score							0.847
≤ 7 (C.10)	5	926	0.92 (0.72 ~ 1.16)	0.472	35.6	0.184	
> 7 (C.11)	2	733	0.98 (0.82 ~ 1.17)	0.825	0.0	0.479	
Sample size							0.179
≤ 1000 (C.12)	4	631	0.81 (0.62 ~ 1.06)	0.121	4.7	0.369	
> 1000 (C.13)	3	1028	1.02 (0.89 ~ 1.17)	0.772	0.0	0.560	
Definition of reference category							0.665
Never breastfeeding (C.14)	3	1019	0.94 (0.80 ~ 1.10)	0.431	0.0	0.458	
Occasional breastfeeding (C.15)	4	640	0.95 (0.69 ~ 1.30)	0.751	37.8	0.186	0.207
≤ 2 months (C.16)	3	602	1.08 (0.88 ~ 1.32)	0.482	0.0	0.489	
≤ 6 months (C.17)	1	38	0.57 (0.30 ~ 1.09)	0.088	NA	NA	

Abbreviations: *PC-CS* population-based case-control study; *HC-CS* hospital-based case-control study; *NA* not applicable

#### Neuroblastoma, soft-tissue sarcoma, nephroblastoma, retinoblastoma, and germ cell tumors

The associations of breastfeeding with risk of childhood neuroblastoma, soft-tissue sarcoma, nephroblastoma, retinoblastoma, and germ cell tumors are presented in Table 5. Significant association was consistently found in neuroblastoma for the two comparisons of breastfeeding versus non/occasional breastfeeding (OR = 0.59, 95% CI 0.44–0.81) and longest versus shortest breastfeeding (OR = 0.61, 95% CI 0.44–0.83).

**Table 5:** Numbers of retrieved studies and pooled odds ratio for the association of breastfeeding with the risk of five individual cancer types

	Ever versus non/occasional breastfeeding					Longest breastfeeding versus shortest breastfeeding				
Cancer type	No. of studies	OR (95%CI)	*P* value	*I*^2^ (%)	*P* value for heterogeneity	No. of studies	OR (95%CI)	*P* value	*I*^2^ (%)	*P* value for heterogeneity
Neuroblastoma	4	0.59 (0.44 ~ 0.81)	0.001	29.2	0.237	4	0.61 (0.44 ~ 0.83)	0.002	0.0	0.482
Soft-tissue sarcoma	4	0.79 (0.44 ~ 1.44)	0.448	37.1	0.189	3	0.69 (0.14 ~ 3.44)	0.649	63.9	0.063
Nephroblastoma	3	0.63 (0.41 ~ 0.98)	0.041	57.2	0.097	3	0.76 (0.44 ~ 1.32)	0.324	49.1	0.140
Retinoblastoma	2	1.32 (0.17 ~ 10.24)	0.792	72.6	0.056	2	1.08 (0.57 ~ 2.07)	0.807	0.0	0.411
Germ cell tumors	2	1.08 (0.73 ~ 1.60)	0.708	0.0	0.639	2	2.64 (1.05 ~ 6.60)	0.038	0.0	0.319

## Discussion

### Study quality and study design

Of the 45 studies included, 33 were sufficient to provide at least fair quality evidence regarding the association between breastfeeding and the risk of childhood cancer. The included studies are at risk for selection bias for cases and controls and the potential misclassification introduced by the lack of specificity in exposure definition. Confounding is also an important consideration on account of the nature of observational studies. Almost all studies matched cases with controls by sex (33/45) and age (37/45), and most studies also matched participants using geographic location (15/45), and a few additionally used race or ethnicity (6/45). These matching variables are crucial for the comparability of cases and controls. Moreover, parental socioeconomic status (SES) was commonly used as adjustment, because higher SES among controls may overestimate the protective effect of breastfeeding on the risk of childhood cancer [40]. Smoking during pregnancy was also considered necessary, since it is associated with breastfeeding [74], and it may be related with the risk of childhood cancer [75, 76], even though the association may vary by cancer types [77–80]. Therefore, the imbalance in this factor between cases and controls may be contributory to confounding bias. Some other potential confounders were also taken into consideration, such as infectious exposures, day care, birth weight, and birth order. However, we cannot exclude the possibility of residual confounding, although most of the included studies had matching and adjustment variables. In the stratified analysis of study quality, we found that the results from studies with higher quality showed consistent association with overall risk estimates, which provides additional confidence in the findings of our meta-analysis.

Case-control study is the main study design that all included studies of the meta-analysis except one were case-control studies. Since most studies have collected exposure information through parental interview, case-control studies are susceptible to recall bias and selection bias. Cohort studies are considered to provide more robust estimates than case-control studies; therefore, further cohort studies are needed to provide more evidence on the association and risk of childhood cancer. An optimal study might be conducted within the framework of a large population-based registry or cohort with full access to baseline information regarding demographic characteristics, detailed data of breastfeeding including breastfeeding duration, the use of infant formulas, the main types of milk given, the age of introduction of a range of foods and so on, and collection of medical records to accurately identify all diagnosed cases [81]. However, cohort studies would require follow-up periods of several years consuming manpower, material, financial and time largely, and very large sample sizes to provide sufficient statistical power. To be noted, the findings are to be expected from International Childhood Cancer Consortium (I4C), which is the first cohort consortium to have published findings on childhood cancer to elaborate the association [82]. But for now, case-control studies are mainly reported, and results from population-based case-control studies are more reliable compared with those from hospital-based case-control studies. We also found similar association in population-based case-control studies with overall risk estimates.

### Leukemia

There is sufficient evidence to show that breastfeeding was inversely associated with the risk by pooling a number of original articles, with 23% lower risk of childhood leukemia (95% CI 9–35%) for breastfeeding versus non/occasional breastfeeding and 23% lower risk (95% CI 6–37%) for longest versus shortest breastfeeding duration, respectively. Moreover, we found a protective effect of breastfeeding on the risk of childhood ALL among the studies with higher quality and population-based case-control studies.

We found high heterogeneity in the meta-analysis on the association between breastfeeding and the risk of childhood leukemia, and there exists significant heterogeneity in different regional groups by meta-regression analysis. In the subgroup analysis of geographic location, we found that breastfeeding was more strongly associated with the risk of childhood leukemia in Asia. In terms of risk of bias, Robins-I tool rated 4 European studies and 2 Asian studies at serious risk, and 10 European studies and 6 Asian studies were considered at moderate risk. At the original study level, one aspect that deserves particular attention is the difference in the number of cases and the source of population, which may at least in part explain the heterogeneity observed across the geographic region. For example, the Asian subgroup analyses included 8 studies with a total of 1739 cases, with only 1 study enrolled more than 200 cases. Moreover, few studies (3/8) used population-based case-control study design. However, European articles included 14 studies with 7518 cases and 71% studies used population controls. This is a similar situation to North America or Oceania, in that a majority (6/11) of articles included in the meta-analysis had more than 200 case numbers, and most (9/11) used population controls. Therefore, the differences in risk estimates could be related to study quality issues. On the other hand, there were several other potential explanations could be proposed. A great variation in breastfeeding duration across the countries may result in the heterogeneity. It should be noted that breastfeeding duration is shorter in high-income countries than in those that are resource-poor. It was estimated that 25% of infants in the USA and Europe are exclusively breastfed through 6 months [83, 84], as compared with 43% in the South-East Asia region [85]. In particular, only three countries (France, Spain, and the USA) had rates below 80% for ever breastfeeding across all country groups [10]. It could be that varying cultural influences contribute to this region disparity, with for example protective Islamic beliefs, South Asian cultural teachings, and more extensive support networks. A study in UK provided the evidence that Pakistan-origin mothers had higher breastfeeding initiation rates and longer average breastfeeding durations than White British mothers [86]. Another cross-sectional study in the USA demonstrated that Asian women had the highest breastfeeding initiation rates relative to all other racial/ethnic groups [87]. On the other hand, the heterogeneity may also due to the breastfeeding pattern. The volume of breastfeeding differed considerably between the women who breastfed only and those who performed mixed feeding, even if these women had the same breastfeeding duration. Globally, the prevalence of exclusive breastfeeding varied widely, countries from Asia and the Pacific region had moderate to high rate of exclusive breastfeeding, while the rate of exclusive breastfeeding was lower in Europe and America [10].

Additionally, we performed the dose-response meta-analysis, which showed a specific non-linear dose-response relationship between breastfeeding and the risk of childhood leukemia. World Health Organization (WHO) and United Nations International Children’s Emergency Fund (UNICEF) developed the global strategy for infant and young child feeding that infants should be exclusively breastfed for the first 6 months of life to achieve optimal growth, development and health [88]. In the current study, we found that breastfeeding duration of 6 months could reduce 20% (95% CI 15–25%) risk of childhood leukemia. The U-shaped curve showed that breastfeeding duration of approximately 9.6 months might show the most significant protect effect on the risk of childhood leukemia. The decreased risk of childhood leukemia was statistically significant at a duration of 4.4–15.0 months. Interestingly, we found that there was more pronounced association between breastfeeding and risk of childhood leukemia when defining breastfeeding for less than 6 months as reference group, rather than never breastfeeding, which may due to that breastfeeding appeared a protective effect on the risk of leukemia after a certain period. The nonsignificant decreased risk at prolonged breastfeeding duration might derive from relatively small sample size but not real effect. Large-sample and well-designed studies should be developed in future to demonstrate this turning point.

There were several potential explanations why breastfeeding may decrease the risk of childhood leukemia. Breast milk contains high levels of immunologically active components and multifactorial anti-inflammatory defense mechanisms that influence the development of the immune system of the breastfed infant [89, 90]. For example, soluble tumor necrosis factor (TNF)-related apoptosis-inducing ligand (TRAIL) in breast milk can control apoptosis and cell proliferation in various organs and tissues. Breastfeeding also provides the infants with human alpha-lactalbumin made lethal to tumor cells (HAMLET), which is a substance with anticancer activity in breast milk [91]. Besides, breast milk imparts the mother’s stem cells to the infant, where they potentially function to actively stimulate or modulate the immune system and promote its development early in life [92]. In particular, Greaves’ hypothesis proposed immunological model that breast milk could modify the immune response in the prevention of childhood ALL [93]. Moreover, accumulating evidence have demonstrated that breast milk has the potential of shaping the neonate’s gut microbiome, such as microbiota richness, diversity, and composition [94, 95]. Recently, a large, multi-center study suggested that breastfeeding status was the most significant factor associated with microbiome structure in early life [96]. It is hypothesized that breastfeeding could decrease the risk of childhood leukemia by the recent discovery of the breast milk microbiome and its connections with immune factors [97].

### Lymphoma

The meta-analysis provided no convincing evidence of the association between breastfeeding and risk of lymphoma. The findings of the present study are consistent with previous observational studies although some early studies indicated negative association of breastfeeding with lymphoma risk, especially in Hodgkin’s lymphoma [19, 43]. In addition, results from sensitivity analysis were unstable, which deserves more studies to clarify the association.

Our pooled analysis presented an inverse association between breastfeeding and risk of childhood lymphoma in Asia, but not Europe or North America, suggesting a potential region-specific effect. What is more, significant discrepancies across subgroups stratified by definition of reference category were found, and only the pooled estimate for the studies using occasional breastfeeding as reference group showed a significantly decreased risk of childhood lymphoma. It may be explained by the significant non-linear dose-response relationship. For example, Davis et al. [33] found that breastfeeding for more than 6 months was associated with a decreased risk of childhood lymphoma, but that breastfeeding for shorter durations was not associated with a reduced risk. The study using breastfeeding for less than 6 months as reference group suggested that a longer breastfeeding duration had a protective effect against Hodgkin’s lymphoma [53]. Another point we should make is that the incidence of lymphoma is highest among 10–14 years old (and even higher among 15–19 years old), while the low age of the cases in the included studies with a short tumor induction period might be too short to find a decreased risk for lymphoma.

### Other cancers

We found significant association between breastfeeding and risk of childhood neuroblastoma, while no significant associations of breastfeeding with risk of soft-tissue sarcoma, brain tumors, nephroblastoma, retinoblastoma, and germ cell tumors. This is updated from the previous meta-analysis and the results were consistent [19]. However, the associations of breastfeeding and risk of these cancers may be underpowered because of the small number of studies in the meta-analysis.

### Strength and limitation

The primary strength of our study is that the traditional categorical meta-analysis and dose-response analysis were applied simultaneously, which can provide more meaningful information. Another strength is the large sample size and number of included studies, which make the findings stable and reliable and enable us to conduct multiple subgroup analyses by geographic location, study quality score, study design, etc. In addition, we also performed stratified analyses on the association of breastfeeding and risk of the subtypes of individual cancers.

However, the present study has several limitations. First, there was high evidence of heterogeneity across studies in the categorical meta-analysis. However, meta-regression analyses suggested that geographic region and definition of reference category are the potential sources of the observed heterogeneity. Second, our meta-analysis included very limited studies from Oceania and no study from Africa. Third, the results of dose-response meta-analysis were prone to be influenced by possible exposure misclassification as the exposure dose was estimated with median for interval exposure, and the lower bound added to the half of the adjacent previous category for the highest open-ended exposure group. Fourth, the number of studies evaluating the associations of breastfeeding with risk of neuroblastoma, nephroblastoma, retinoblastoma, and germ cell tumors is small. Fifth, we were unable to assess differences by age or sex, because sufficiently age- or sex-specific studies are not available. Finally, limited by the lack information of breastfeeding pattern in most of included studies, such as exclusive breastfeeding and partial breastfeeding, we cannot evaluate the association between breastfeeding pattern and the risk of childhood cancer.

## Conclusion

This meta-analysis demonstrates that breastfeeding was associated with the reduced the risk of childhood leukemia. The present study also provides suggestive evidence of the inverse association between breastfeeding and risk of neuroblastoma. In addition, given that the role of breastfeeding for the risk of childhood leukemia and lymphoma may be region-specific, further analyses are warranted to provide insights into the strategy of breastfeeding advocacy.

## Supplementary Information

**Additional file 1: Table S1.** The PRISMA 2009 checklist for this meta-analysis. **Figure S1.** Flowchart of study selection. **Table S2.** List of excluded studies along with reason. **Table S3.** Details of included studies for subgroup analysis. **Figure S2.** Begg’s funnel plots identifying the publication bias for the association between breastfeeding and risk of childhood leukemia. **Figure S3.** Forest plots of subgroup analysis of association between breastfeeding and childhood leukemia risk in the order listed in Table 2. **Table S4.** Subgroup analyses of the association between breastfeeding and acute lymphoblastic leukemia risk. **Figure S4.** One-study-removed analysis on the association of breastfeeding with risk of (A) childhood leukemia and (B) acute lymphoblastic leukemia. **Figure S5.** Pooled analysis of studies including only children aged 0-14 years old for the for the association of breastfeeding with risk of (A) childhood leukemia and (B) acute lymphoblastic leukemia. **Figure S6.** Risk estimates (solid line) and the corresponding 95% CIs (dash lines) for the dose-response relationship between breastfeeding and the risk of childhood lymphoma. **Figure S7.** Begg’s funnel plots identifying the publication bias for the association between breastfeeding and risk of childhood lymphoma. **Figure S8.** Forest plots of subgroup analysis of association between breastfeeding and childhood lymphoma risk in the order listed in Table 3. **Figure S9.** One-study-removed analysis on the association of breastfeeding with risk of childhood lymphoma. **Figure S10.** Pooled analysis of studies including only children aged 0-14 years old for the for the association of breastfeeding with risk of childhood lymphoma. **Figure S11.** Begg’s funnel plots identifying the publication bias for the association between breastfeeding and risk of childhood brain tumors. **Figure S12.** Forest plots of subgroup analysis of association between breastfeeding and risk of childhood brain tumors in the order listed in Table 4. **Figure S13.** One-study-removed analysis on the association of breastfeeding with risk of childhood brain tumors. **Figure S14.** Pooled analysis of studies including only children aged 0–14 years old for the for the association of breastfeeding with risk of childhood brain tumors.

## Data Availability

All data generated or analyzed during this study are included in this article and its additional files.

## Abbreviations

CI: Confidence interval
HAMLET: Human alpha-lactalbumin made lethal to tumor cells
OR: Odds ratio
PRISMA: Preferred Reporting Items for Systematic reviews and Meta-Analysis
RR: Relative risk
TNF: Tumor necrosis factor
TRAIL: TNF-related apoptosis-inducing ligand
UNICEF: United Nations International Children’s Emergency Fund
WHO: World Health Organization
