# Supplementary material for: Breastfeeding and the risk of childhood cancer: a systematic review and dose-response meta-analysis
Source: BMC Med. 2021 Apr 13;19:90. doi: 10.1186/s12916-021-01950-5 (PMC8042913; doi:10.1186/s12916-021-01950-5)
Supplement: Supplementary file 1 — Additional file 1: Table S1. The PRISMA 2009 checklist for this meta-analysis. Figure S1. Flowchart of study selection. Table S2. List of excluded studies along with reason. Table S3. Details of included studies for subgroup analysis. Figure S2. Begg’s funnel plots identifying the publication bias for the association between breastfeeding and risk of childhood leukemia. Figure S3. Forest plots of subgroup analysis of association between breastfeeding and childhood leukemia risk in the order listed in Table 2. Table S4. Subgroup analyses of the association between breastfeeding and acute lymphoblastic leukemia risk. Figure S4. One-study-removed analysis on the association of breastfeeding with risk of (A) childhood leukemia and (B) acute lymphoblastic leukemia. Figure S5. Pooled analysis of studies including only children aged 0-14 years old for the for the association of breastfeeding with risk of (A) childhood leukemia and (B) acute lymphoblastic leukemia. Figure S6. Risk estimates (solid line) and the corresponding 95% CIs (dash lines) for the dose-response relationship between breastfeeding and the risk of childhood lymphoma. Figure S7. Begg’s funnel plots identifying the publication bias for the association between breastfeeding and risk of childhood lymphoma. Figure S8. Forest plots of subgroup analysis of association between breastfeeding and childhood lymphoma risk in the order listed in Table 3. Figure S9. One-study-removed analysis on the association of breastfeeding with risk of childhood lymphoma. Figure S10. Pooled analysis of studies including only children aged 0-14 years old for the for the association of breastfeeding with risk of childhood lymphoma. Figure S11. Begg’s funnel plots identifying the publication bias for the association between breastfeeding and risk of childhood brain tumors. Figure S12. Forest plots of subgroup analysis of association between breastfeeding and risk of childhood brain tumors in the order listed in Table 4. Figure S13. [file 12916_2021_1950_MOESM1_ESM.docx]

**Additional file 1**

**Table S1** The PRISMA 2009 checklist for this meta-analysis.

**Fig. S1** Flowchart of study selection.

**Table S2** List of excluded studies along with reason.

**Table** **S3** Details of included studies for subgroup analysis.

**Fig. S2** Begg’s funnel plots identifying the publication bias for the association between breastfeeding and risk of childhood leukemia.

**Fig. S3** Forest plots of subgroup analysis of association between breastfeeding and childhood leukemia risk in the order listed in Table 2.

**Table S4** Subgroup analyses of the association between breastfeeding and acute lymphoblastic leukemia risk.

**Fig. S4** One-study-removed analysis on the association of breastfeeding with risk of (A) childhood leukemia and (B) acute lymphoblastic leukemia.

**Fig. S5** Pooled analysis of studies including only children aged 0-14 years old for the for the association of breastfeeding with risk of (A) childhood leukemia and (B) acute lymphoblastic leukemia.

**Fig. S6** Risk estimates (solid line) and the corresponding 95% CIs (dash lines) for the dose-response relationship between breastfeeding and the risk of childhood lymphoma.

**Fig. S7** Begg’s funnel plots identifying the publication bias for the association between breastfeeding and risk of childhood lymphoma.

**Fig. S8** Forest plots of subgroup analysis of association between breastfeeding and childhood lymphoma risk in the order listed in Table 3.

**Fig. S9** One-study-removed analysis on the association of breastfeeding with risk of childhood

lymphoma.

**Fig. S10** Pooled analysis of studies including only children aged 0-14 years old for the for the association of breastfeeding with risk of childhood lymphoma.

**Fig. S11** Begg’s funnel plots identifying the publication bias for the association between breastfeeding and risk of childhood brain tumors.

**Fig. S12** Forest plots of subgroup analysis of association between breastfeeding and risk of childhood brain tumors in the order listed in Table 4.

**Fig. S13** One-study-removed analysis on the association of breastfeeding with risk of childhood brain tumors.

**Fig. S14** Pooled analysis of studies including only children aged 0-14 years old for the for the association of breastfeeding with risk of childhood brain tumors.

| **Table S1. The PRISMA 2009 checklist for this meta-analysis ^a^** | | | |
| --- | --- | --- | --- |
| Section/topic | # | Checklist item | Reported on page # |
| TITLE | | |  |
| Title | 1 | Identify the report as a systematic review, meta-analysis, or both. | 1 |
| ABSTRACT | | |  |
| Structured summary | 2 | Provide a structured summary including, as applicable: background; objectives; data sources; study eligibility criteria, participants, and interventions; study appraisal and synthesis methods; results; limitations; conclusions and implications of key findings; systematic review registration number. | 2 |
| INTRODUCTION | | |  |
| Rationale | 3 | Describe the rationale for the review in the context of what is already known. | 3-4 |
| Objectives | 4 | Provide an explicit statement of questions being addressed with reference to participants, interventions, comparisons, outcomes, and study design (PICOS). | 4 |
| METHODS | | |  |
| Protocol and registration | 5 | Indicate if a review protocol exists, if and where it can be accessed (e.g., Web address), and, if available, provide registration information including registration number. | 4 |
| Eligibility criteria | 6 | Specify study characteristics (e.g., PICOS, length of follow-up) and report characteristics (e.g., years considered, language, publication status) used as criteria for eligibility, giving rationale. | 4-5 |
| Information sources | 7 | Describe all information sources (e.g., databases with dates of coverage, contact with study authors to identify additional studies) in the search and date last searched. | 4 |
| Search | 8 | Present full electronic search strategy for at least one database, including any limits used, such that it could be repeated. | 4 |
| Study selection | 9 | State the process for selecting studies (i.e., screening, eligibility, included in systematic review, and, if applicable, included in the meta-analysis). | 5 |
| Data collection process | 10 | Describe method of data extraction from reports (e.g., piloted forms, independently, in duplicate) and any processes for obtaining and confirming data from investigators. | 5 |
| Data items | 11 | List and define all variables for which data were sought (e.g., PICOS, funding sources) and any assumptions and simplifications made. | 5 |
| Risk of bias in individual studies | 12 | Describe methods used for assessing risk of bias of individual studies (including specification of whether this was done at the study or outcome level), and how this information is to be used in any data synthesis. | 5 |
| Summary measures | 13 | State the principal summary measures (e.g., risk ratio, difference in means). | 6 |
| Synthesis of results | 14 | Describe the methods of handling data and combining results of studies, if done, including measures of consistency (e.g., I^2^) for each meta-analysis. | 6 |
| Risk of bias across studies | 15 | Specify any assessment of risk of bias that may affect the cumulative evidence (e.g., publication bias, selective reporting within studies). | 7 |
| Additional analyses | 16 | Describe methods of additional analyses (e.g., sensitivity or subgroup analyses, meta-regression), if done, indicating which were pre-specified. | 6-7 |
| RESULTS | | |  |
| Study selection | 17 | Give numbers of studies screened, assessed for eligibility, and included in the review, with reasons for exclusions at each stage, ideally with a flow diagram. | 7 |
| Study characteristics | 18 | For each study, present characteristics for which data were extracted (e.g., study size, PICOS, follow-up period) and provide the citations. | 7-8 |
| Risk of bias within studies | 19 | Present data on risk of bias of each study and, if available, any outcome level assessment (see item 12). | 7-8 |
| Results of individual studies | 20 | For all outcomes considered (benefits or harms), present, for each study: (a) simple summary data for each intervention group (b) effect estimates and confidence intervals, ideally with a forest plot. | 8-11 |
| Synthesis of results | 21 | Present results of each meta-analysis done, including confidence intervals and measures of consistency. | 8-11 |
| Risk of bias across studies | 22 | Present results of any assessment of risk of bias across studies (see Item 15). | 8-11 |
| Additional analysis | 23 | Give results of additional analyses, if done (e.g., sensitivity or subgroup analyses, meta-regression [see Item 16]). | 8-11 |
| DISCUSSION | | |  |
| Summary of evidence | 24 | Summarize the main findings including the strength of evidence for each main outcome; consider their relevance to key groups (e.g., healthcare providers, users, and policy makers). | 11-17 |
| Limitations | 25 | Discuss limitations at study and outcome level (e.g., risk of bias), and at review-level (e.g., incomplete retrieval of identified research, reporting bias). | 17 |
| Conclusions | 26 | Provide a general interpretation of the results in the context of other evidence, and implications for future research. | 18 |
| FUNDING | | |  |
| Funding | 27 | Describe sources of funding for the systematic review and other support (e.g., supply of data); role of funders for the systematic review. | 18 |
| ^a^ Moher D, Liberati A, Tetzlaff J, Altman DG, The PRISMA Group (2009). Preferred Reporting Items for Systematic Reviews and Meta-Analyses: The PRISMA Statement. PLoS Med 6(6): e1000097. doi:10.1371/journal.pmed1000097; For more information, visit: www.prisma-statement.org. | | | |

**
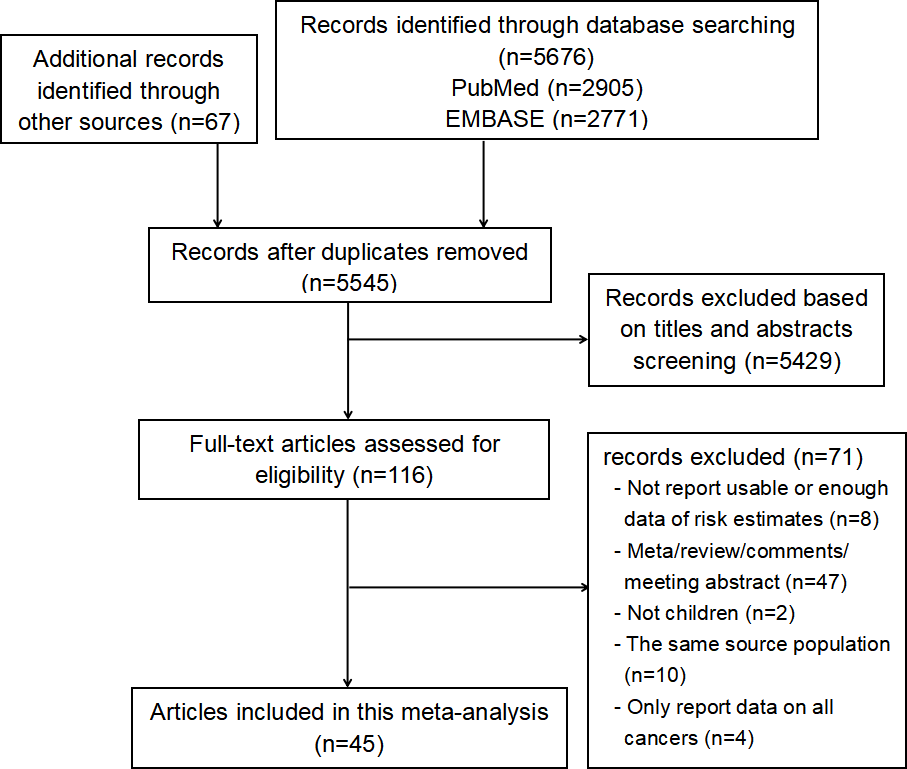
**

**Fig. S1** Flowchart of study selection

| **Table S2** List of excluded studies along with reason. | | |
| --- | --- | --- |
|  | **Study** | **Reason for exclusion** |
| 1 | Birch JM, Hartley AL, Teare MD, et al. The inter-regional epidemiological study of childhood cancer (IRESCC): case-control study of children with central nervous system tumours. Br J Neurosurg. 1990;4(1):17-25. | Not report usable or enough data of risk estimates |
| 2 | Schwartzbaum JA, George SL, Pratt CB, et al. An exploratory study of environmental and medical factors potentially related to childhood cancer. Med Pediatr Oncol. 1991;19(2):115-21. | Not report usable or enough data of risk estimates |
| 3 | Kaatsch P, Kaletsch U, Krummenauer F, et al. Case control study on childhood leukemia in Lower Saxony, Germany. Basic considerations, methodology, and summary of results. Klin Padiatr. 1996;208(4):179-85. | Not report usable or enough data of risk estimates |
| 4 | Mwanda OW. Cancers in children younger than age 16 years in Kenya. East Afr Med J. 1999;76(1):3-9. | Not report usable or enough data of risk estimates |
| 5 | Hrusák O, Trka J, Zuna J, et al. Acute lymphoblastic leukemia incidence during socioeconomic transition: selective increase in children from 1 to 4 years. Leukemia. 2002;16(4):720-5. | Not report usable or enough data of risk estimates |
| 6 | Karimi M, Haghighat M, Dialameh Z, et al. Breastfeeding as a Protective Effect Against Childhood Leukemia and Lymphoma. Iran Red Crescent Med J. 2016;18(9):e29771. | Not report usable or enough data of risk estimates |
| 7 | Monroy-Torres R, Ibarra-Miranda D, Naves-Sanchez J, et al. Main Nutritional and Environmental Risk Factors in Children with Leukemia from a Public Hospital of the State of Guanajuato, Mexico. Current Cancer Therapy Reviews. 2018; 14(3). | Not report usable or enough data of risk estimates |
| 8 | Cai YL, Zou Y, Chen XJ, et al. [Epidemidogical Analysis of 982 Hospitalized Cases of Childhood Acute Lymphoblastic Leukemia]. Zhongguo Shi Yan Xue Ye Xue Za Zhi. 2020;28(2):371-376. | Not report usable or enough data of risk estimates |
| 9 | Smigel KL. Breast-feeding linked to decreased cancer risk for mother, child. J Natl Cancer Inst. 1988;80(17):1362-3. | Meta/review/comments/ meeting abstract |
| 10 | Cunningham AS, Jelliffe DB, Jelliffe EF. Breast-feeding and health in the 1980s: a global epidemiologic review. J Pediatr. 1991;118(5):659-66. | Meta/review/comments/ meeting abstract |
| 11 | Davis MK. Review of the evidence for an association between infant feeding and childhood cancer. Int J Cancer Suppl. 1998;11:29-33. | Meta/review/comments/ meeting abstract |
| 12 | Davis MK. Breastfeeding and chronic disease in childhood and adolescence. Pediatr Clin North Am. 2001;48(1):125-41, ix. | Meta/review/comments/ meeting abstract |
| 13 | Parker L. Breast-feeding and cancer prevention. Eur J Cancer. 2001;37(2):155-8. | Meta/review/comments/ meeting abstract |
| 14 | Fear NT, Roman E. Re: Breast-feeding and neuroblastoma, USA and Canada. Cancer Causes Control. 2003;14(3):299; author reply 300. | Meta/review/comments/ meeting abstract |
| 15 | Kwan ML, Buffler PA, Abrams B, et al. Breastfeeding and the risk of childhood leukemia: a meta-analysis. Public Health Rep. 2004;119(6):521-35. | Meta/review/comments/ meeting abstract |
| 16 | McNally RJ, Eden TO. An infectious aetiology for childhood acute leukaemia: a review of the evidence. Br J Haematol. 2004;127(3):243-63. | Meta/review/comments/ meeting abstract |
| 17 | Tripathy AK, Mishra L, Bakhshi S, et al. Breast feeding and childhood hematological malignancy. Indian J Pediatr. 2004;71(5):417-8. | Meta/review/comments/ meeting abstract |
| 18 | Fewtrell M S. The long-term benefits of having been breast-fed. Current Paediatrics. 2004;14(2):97-103. | Meta/review/comments/ meeting abstract |
| 19 | Ferrís i Tortajada J, Ortega García JA, García i Castell J, et al. Factores de riesgo para el neuroblastoma [Risk factors for neuroblastoma]. An Pediatr (Barc). 2005;63(1):50-60. | Meta/review/comments/ meeting abstract |
| 20 | Guise JM, Austin D, Morris CD. Review of case-control studies related to breastfeeding and reduced risk of childhood leukemia. Pediatrics. 2005;116(5):e724-31. | Meta/review/comments/ meeting abstract |
| 21 | Martin RM, Gunnell D, Owen CG, et al. Breast-feeding and childhood cancer: A systematic review with metaanalysis. Int J Cancer. 2005;117(6):1020-31. | Meta/review/comments/ meeting abstract |
| 22 | Schack-Nielsen L, Larnkjaer A, Michaelsen KF. Long term effects of breastfeeding on the infant and mother. Adv Exp Med Biol. 2005;569:16-23. | Meta/review/comments/ meeting abstract |
| 23 | McNally RJ, Parker L. Environmental factors and childhood acute leukemias and lymphomas. Leuk Lymphoma. 2006;47(4):583-98. | Meta/review/comments/ meeting abstract |
| 24 | Schack-Nielsen L, Michaelsen KF. Breast feeding and future health. Curr Opin Clin Nutr Metab Care. 2006;9(3):289-96. | Meta/review/comments/ meeting abstract |
| 25 | Bosnjak AP, Grgurić J. Dugotrajni ucinci dojenja na zdravlje [Long-term health effects of breastfeeding]. Lijec Vjesn. 2007;129(8-9):293-8. | Meta/review/comments/ meeting abstract |
| 26 | Ip S, Chung M, Raman G, et al. Breastfeeding and maternal and infant health outcomes in developed countries. Evid Rep Technol Assess (Full Rep). 2007;(153):1-186. | Meta/review/comments/ meeting abstract |
| 27 | Piper KM, Berry CA, Cregan MD. The bioactive nature of human breastmilk. Breastfeed Rev. 2007;15(3):5-10. | Meta/review/comments/ meeting abstract |
| 28 | Løland BF, Baerug AB, Nylander G. Morsmelk, immunrespons og helseeffekter [Human milk, immune responses and health effects]. Tidsskr Nor Laegeforen. 2007;127(18):2395-8. | Meta/review/comments/ meeting abstract |
| 29 | Ip S, Chung M, Raman G, et al. A summary of the Agency for Healthcare Research and Quality's evidence report on breastfeeding in developed countries. Breastfeed Med. 2009;4 Suppl 1:S17-30. | Meta/review/comments/ meeting abstract |
| 30 | Pinarli F, Oguz A, Congar A, et al. The possible risk factors for childhood cancer. Pediatric Blood and Cancer. 2009;53(3):847 | Meta/review/comments/ meeting abstract |
| 31 | Zur Hausen H. Childhood leukemias and other hematopoietic malignancies: interdependence between an infectious event and chromosomal modifications. Int J Cancer. 2009 Oct;125(8):1764-70. | Meta/review/comments/ meeting abstract |
| 32 | Martin R, Davey Smith G, Gunnell D. Do infants who are breast-fed have an altered risk of developing cancer? Adv Exp Med Biol. 2009;639:167-98. | Meta/review/comments/ meeting abstract |
| 33 | Wali Y, Waly M, Al-Mukhaini Y, et al. The Relationship between Breastfeeding and Childhood Leukemia Risk in Oman. FASEB JOURNAL. 2010. | Meta/review/comments/ meeting abstract |
| 34 | Van Gelder C, Wijers C, Aben K, et al. Prenatal and neonatal risk factors for testicular germ cell cancer. American Journal of Epidemiology. 2011;173(Supplement 11), S1-S316. | Meta/review/comments/ meeting abstract |
| 35 | Prakash A, Yadav S P, Dinand V, et al. Consumption of fruits, vegetables and milk products in childhood decreases the risk of pediatric acute lymphoblastic leukemia. Pediatric Blood & Cancer. 2012;59(6):965-1152. | Meta/review/comments/ meeting abstract |
| 36 | Schraw J, Dong Y Q, Scheurer M E, et al. Abstract A102: Longer formula feeding and later age at introduction of solids increase the odds ratio of pediatric acute lymphoblastic leukemia. Cancer Prevention Research. 2012; 5(11_Supplement):A102-A102. | Meta/review/comments/ meeting abstract |
| 37 | Maia Rda R, Wünsch Filho V. Infection and childhood leukemia: review of evidence. Rev Saude Publica. 2013;47(6):1172-85. | Meta/review/comments/ meeting abstract |
| 38 | Wang KL, Liu CL, Zhuang Y, et al. Breastfeeding and the risk of childhood Hodgkin lymphoma: a systematic review and meta-analysis. Asian Pac J Cancer Prev. 2013;14(8):4733-7. | Meta/review/comments/ meeting abstract |
| 39 | Puumala SE, Ross JA, Aplenc R, et al. Epidemiology of childhood acute myeloid leukemia. Pediatr Blood Cancer. 2013;60(5):728-33. | Meta/review/comments/ meeting abstract |
| 40 | Amitay E, Keinan-Boker L. [Breastfeeding and childhood leukemia and lymphoma]. Harefuah. 2014;153(5):273-9, 305, 304. | Meta/review/comments/ meeting abstract |
| 41 | Nency Y, Fardhani R, Wibawa S, et al. THE CORRELATION BETWEEN HISTORY OF BREASTFEEDING AND THE INCIDENCE OF CHILDHOOD ACUTE LEUKEMIA IN SEMARANG, INDONESIA. Asia-Pacific Journal of Clinical Oncology. 2014;10:144-144. | Meta/review/comments/ meeting abstract |
| 42 | Bols, E, Smits, L, Weijenberg, M. Healthy Living: The European Congress of Epidemiology. European Journal of Epidemiology. 2015;30(8):709-1001. | Meta/review/comments/ meeting abstract |
| 43 | Amitay EL, Keinan-Boker L. Breastfeeding and Childhood Leukemia Incidence: A Meta-analysis and Systematic Review. JAMA Pediatr. 2015;169(6):e151025. | Meta/review/comments/ meeting abstract |
| 44 | Amitay EL, Keinan-Boker L. Breastfeeding and Childhood Leukemia Incidence--Reply. JAMA Pediatr. 2015;169(11):1071-2. | Meta/review/comments/ meeting abstract |
| 45 | Gammill HS, Milano F, Nelson JL. Breastfeeding and Childhood Leukemia Incidence: Duplicate Data Inadvertently Included in the Meta-analysis and Consideration of Possible Confounders. JAMA Pediatr. 2015;169(11):1071. | Meta/review/comments/ meeting abstract |
| 46 | Jeremy M. Schraw, Michael Scheurer, et al. Abstract 1877: Age at introduction to solids is associated with the odds ratio of pediatric acute lymphoblastic leukemia. Cancer Research. 2015;75(15 Supplement):1877-1877. | Meta/review/comments/ meeting abstract |
| 47 | Gertosio C, Meazza C, Pagani S, et al. Breastfeeding and its gamut of benefits. Minerva Pediatr. 2016;68(3):201-12. | Meta/review/comments/ meeting abstract |
| 48 | Kannan Kutty P. Breastfeeding counsel against cancers. Asian Pacific Journal of Tropical Biomedicine. 2016;6(5):422-428. | Meta/review/comments/ meeting abstract |
| 49 | Michie C. Breast feeding could reduce the risk of childhood leukaemias. Evid Based Nurs. 2016;19(3):83. | Meta/review/comments/ meeting abstract |
| 50 | Metayer C, Dahl G, Wiemels J, et al. Childhood Leukemia: A Preventable Disease. Pediatrics. 2016 Nov;138(Suppl 1):S45-S55. | Meta/review/comments/ meeting abstract |
| 51 | Whitehead TP, Metayer C, Wiemels JL, et al. Childhood Leukemia and Primary Prevention. Curr Probl Pediatr Adolesc Health Care. 2016;46(10):317-352. | Meta/review/comments/ meeting abstract |
| 52 | Mohammadian A, Mahdavifar N. Investigating the relationship between breastfeeding with childhood leukemia in sistan and baluchestan province. Iranian Journal of Blood and Cancer. 2017. | Meta/review/comments/ meeting abstract |
| 53 | Stuebe A M. Abstract IA23: Reducing cancer risk by enabling women to breastfeed. Abstracts: AACR Special Conference: Improving Cancer Risk Prediction for Prevention and Early Detection November 16-19, 2016 Orlando, FL. 2017. | Meta/review/comments/ meeting abstract |
| 54 | Brahm P, Valdés V. Beneficios de la lactancia materna y riesgos de no amamantar [The benefits of breastfeeding and associated risks of replacement with baby formulas]. Rev Chil Pediatr. 2017;88(1):7-14. | Meta/review/comments/ meeting abstract |
| 55 | Güngör D, Nadaud P, Dreibelbis C, et al. Infant milk-feeding practices and childhood leukemia: a systematic review. Am J Clin Nutr. 2019;109(Suppl_7):757S-771S. | Meta/review/comments/ meeting abstract |
| 56 | Rudant J, Lightfoot T, Urayama KY, et al. Childhood acute lymphoblastic leukemia and indicators of early immune stimulation: a Childhood Leukemia International Consortium study. Am J Epidemiol. 2015;181(8):549-62. | The same source population |
| 57 | Rudant J, Orsi L, Bonaventure A, et al. ARID5B, IKZF1 and non-genetic factors in the etiology of childhood acute lymphoblastic leukemia: the ESCALE study. PLoS One. 2015;10(3):e0121348. | The same source population |
| 58 | Crouch S, Lightfoot T, Simpson J, et al. Infectious illness in children subsequently diagnosed with acute lymphoblastic leukemia: modeling the trends from birth to diagnosis. Am J Epidemiol. 2012;176(5):402-8. | The same source population |
| 59 | Mallol-Mesnard N, Menegaux F, Lacour B, et al. Birth characteristics and childhood malignant central nervous sytem tumors: the ESCALE study (French Society for Childhood Cancer). Cancer Detect Prev. 2008;32(1):79-86. | The same source population |
| 60 | Harding NJ, Birch JM, Hepworth SJ, et al. Breastfeeding and risk of childhood CNS tumours. Br J Cancer. 2007;96(5):815-7. | The same source population |
| 61 | Kwan ML, Buffler PA, Wiemels JL, et al. Breastfeeding patterns and risk of childhood acute lymphoblastic leukaemia. Br J Cancer. 2005;93(3):379-84. | The same source population |
| 62 | Menegaux F, Olshan AF, Neglia JP, et al. Day care, childhood infections, and risk of neuroblastoma. Am J Epidemiol. 2004;159(9):843-51. | The same source population |
| 63 | Perrillat F, Clavel J, Auclerc MF, et al. Day-care, early common infections and childhood acute leukaemia: a multicentre French case-control study. Br J Cancer. 2002 8;86(7):1064-9. | The same source population |
| 64 | Bener A, Denic S, Galadari S. Longer breast-feeding and protection against childhood leukaemia and lymphomas. Eur J Cancer. 2001;37(2):234-8. | The same source population |
| 65 | McKinney PA, Juszczak E, Findlay E, et al. Pre- and perinatal risk factors for childhood leukaemia and other malignancies: a Scottish case control study. Br J Cancer. 1999;80(11):1844-51. | The same source population |
| 66 | Feltbower RG, Fleming SJ, Picton SV, et al. UK case control study of brain tumours in children, teenagers and young adults: a pilot study. BMC Res Notes. 2014;7:14. | Not among children |
| 67 | Frentzel-Beyme R, Becher H, Salzer-Kuntschik M, et al. Factors affecting the incident juvenile bone tumors in an Austrian case-control study. Cancer Detect Prev. 2004;28(3):159-69. | Not among children |
| 68 | Hartley AL, Birch JM, McKinney PA, et al. The Inter-Regional Epidemiological Study of Childhood Cancer (IRESCC): past medical history in children with cancer. J Epidemiol Community Health. 1988;42(3):235-42. | Only report data on all cancers |
| 69 | Golding J, Paterson M, Kinlen LJ. Factors associated with childhood cancer in a national cohort study. Br J Cancer. 1990;62(2):304-8. | Only report data on all cancers |
| 70 | Ortega-García JA, Ferrís-Tortajada J, Torres-Cantero AM, et al. Full breastfeeding and paediatric cancer. J Paediatr Child Health. 2008;44(1-2):10-3. | Only report data on all cancers |
| 71 | Küçükçongar A, Oğuz A, Pınarlı FG, et al. Breastfeeding and Childhood Cancer: Is Breastfeeding Preventative to Childhood Cancer? Pediatr Hematol Oncol. 2015;32(6):374-81. | Only report data on all cancers |

| **Table S3** Details of included studies for subgroup analysis. | |
| --- | --- |
|  | **Included studies** |
| A.1 | Magnani et al. [33], Shu et al. [35], Shu et al. [38], UKCCS Investigators. [42], Hardell et al. [14], Perrillat et al. [46], Jourdan-Da et al. [47], Altinkaynak et al. [16], Rudant et al. [54], Mattioli et al. [59], Jiménez-Hernández et al. [18] |
| A.2 | Davis et al. [15], Magnani et al. [33], van Duijn et al. [34], Shu et al. [35], Schüz et al. [37], Shu et al. [38], Dockerty et al. [40], Infante-Rivard et al. [41], UKCCS Investigators. [42], Hardell et al. [14], Murray et al. [45], Perrillat et al. [46], Lancashire et al. [13], Jourdan-Da et al. [47], Altinkaynak et al. [16], Bener et al. [51], MacArthur et al. [52], Flores-Lujano et al. [53], Rudant et al. [54], Waly et al. [56], Urayama et al. [57], Schraw et al. [60], Ajrouche et al. [61], Greenop et al. [62], Schraw et al. [67], Jiménez-Hernández et al. [18], Rafieemehr et al. [70] |
| A.3 | Schüz et al. [37], Shu et al. [38], UKCCS Investigators. [42], Jourdan-Da et al. [47], Rudant et al. [54], Ajrouche et al. [61] |
| A.4 | Shu et al. [38], UKCCS Investigators. [42], Rudant et al. [54], Ajrouche et al. [61] |
| A.5 | Davis et al. [15], Magnani et al. [33], van Duijn et al. [34], Shu et al. [35], Petridou et al. [36], Schüz et al. [37], Shu et al. [38], Smulevich et al. [39], Dockerty et al. [40], Infante-Rivard et al. [41] |
| A.6 | UKCCS Investigators. [42], Hardell et al. [14], Murray et al. [45], Perrillat et al. [46], Lancashire et al. [13], Jourdan-Da et al. [47], Altinkaynak et al. [16], Bener et al. [51], MacArthur et al. [52], Flores-Lujano et al. [53], Rudant et al. [54], Waly et al. [56], Urayama et al. [57], Mattioli et al. [59], Schraw et al. [60], Ajrouche et al. [61], Greenop et al. [62], Amitay et al. [64], Schraw et al. [67], Gao et al. [68], Jiménez-Hernández et al. [18], Lingappa et al. [69], Rafieemehr et al. [70] |
| A.7 | Magnani et al. [33], van Duijn et al. [34], Petridou et al. [36], Schüz et al. [37], Smulevich et al. [39], UKCCS Investigators. [42], Hardell et al. [14], Murray et al. [45], Perrillat et al. [46], Lancashire et al. [13], Jourdan-Da et al. [47], Rudant et al. [54], Mattioli et al. [59], Ajrouche et al. [61] |
| A.8 | Shu et al. [35], Altinkaynak et al. [16], Bener et al. [51], Waly et al. [56], Amitay et al. [64], Gao et al. [68], Lingappa et al. [69], Rafieemehr et al. [70] |
| A.9 | Davis et al. [15], Shu et al. [38], Dockerty et al. [40], Infante-Rivard et al. [41], MacArthur et al. [52], Flores-Lujano et al. [53], Urayama et al. [57], Schraw et al. [60], Greenop et al. [62], Schraw et al. [67], Jiménez-Hernández et al. [18] |
| A.10 | Davis et al. [15], Infante-Rivard et al. [41], MacArthur et al. [42], Flores-Lujano et al. [53], Urayama et al. [57], Schraw et al. [60], Schraw et al. [67], Jiménez-Hernández et al. [18] |
| A.11 | Dockerty et al. [40], Greenop et al. [62] |
| A.12 | Shu et al. [38] |
| A.13 | Magnani et al. [33], Murray et al. [45], Bener et al. [51], MacArthur et al. [52], Flores-Lujano et al. [53], Waly et al. [56], Mattioli et al. [59], Jiménez-Hernández et al. [18], Lingappa et al. [69], Rafieemehr et al. [70] |
| A.14 | Davis et al. [15], van Duijn et al. [34], Shu et al. [35], Petridou et al. [36], Schüz et al. [37], Shu et al. [38], Smulevich et al. [39], Dockerty et al. [40], Infante-Rivard et al. [41], UKCCS Investigators. [42], Hardell et al. [14], Perrillat et al. [46], Lancashire et al. [13], Jourdan-Da et al. [47], Altinkaynak et al. [16], Rudant et al. [54], Urayama et al. [57], Schraw et al. [60], Ajrouche et al. [61], Greenop et al. [62], Amitay et al. [64], Schraw et al. [67], Gao et al. [68] |
| A.15 | Davis et al. [15], Magnani et al. [33], Shu et al. [35], Petridou et al. [36], Dockerty et al. [40], Hardell et al. [14], Perrillat et al. [46], Altinkaynak et al. [16], Bener et al. [51], Flores-Lujano et al. [53], Waly et al. [56], Schraw et al. [60], Amitay et al. [64], Lingappa et al. [69], Rafieemehr et al. [70] |
| A.16 | van Duijn et al. [34], Schüz et al. [37], Shu et al. [38], Smulevich et al. [39], Infante-Rivard et al. [41], UKCCS Investigators. [42], Murray et al. [45], Lancashire et al. [13], Jourdan-Da et al. [47], MacArthur et al. [52], Rudant et al. [54], Urayama et al. [57], Mattioli et al. [59], Ajrouche et al. [61], Greenop et al. [62], Schraw et al. [67], Gao et al. [68], Jiménez-Hernández et al. [18] |
| A.17 | Davis et al. [15], van Duijn et al. [34], Shu et al. [35], Schüz et al. [37], Shu et al. [38], Smulevich et al. [39], Dockerty et al. [40], Infante-Rivard et al. [41], UKCCS Investigators. [42], Hardell et al. [14], Lancashire et al. [13], Jourdan-Da et al. [47], Altinkaynak et al. [16], MacArthur et al. [52], Rudant et al. [54], Urayama et al. [57], Mattioli et al. [59], Schraw et al. [60], Ajrouche et al. [61], Greenop et al. [62], Amitay et al. [64], Schraw et al. [67] |
| A.18 | Magnani et al. [33], Petridou et al. [36], Perrillat et al. [46], Bener et al. [51], Flores-Lujano et al. [53], Waly et al. [56], Gao et al. [68], Jiménez-Hernández et al. [18], Lingappa et al. [69], Rafieemehr et al. [70] |
| A.19 | Murray et al. [45] |
| A.20 | Magnani et al. [33], van Duijn et al. [34], Shu et al. [35], Petridou et al. [36], Shu et al. [38], Dockerty et al. [40], Infante-Rivard et al. [41], UKCCS Investigators. [42], Murray et al. [45], Perrillat et al. [46], Lancashire et al. [13], Jourdan-Da et al. [47], MacArthur et al. [52], Flores-Lujano et al. [53], Rudant et al. [54], Urayama et al. [57], Mattioli et al. [59], Schraw et al. [60], Ajrouche et al. [61], Greenop et al. [62], Schraw et al. [66], Gao et al. [68], Jiménez-Hernández et al. [18], Lingappa et al. [69], Rafieemehr et al. [70] |
| A.21 | Davis et al. [15], Schüz et al. [37], Smulevich et al. [39], Hardell et al. [14], Altinkaynak et al. [16], Bener et al. [51], Waly et al. [56], Amitay et al. [64] |
| A.22 | Davis et al. [15], Altinkaynak et al. [16], Bener et al. [51], Waly et al. [56], Amitay et al. [64] |
| A.23 | Schüz et al. [37], Smulevich et al. [39], Hardell et al. [14] |
| B.1 | Davis et al. [15], Shu et al. [35], Smulevich et al. [39], UKCCS Investigators. [42], Hardell et al. [14], Altinkaynak et al. [16], Petridou et al. [50], Bener et al. [51], Rudant et al. [55] |
| B.2 | Davis et al. [15], Magnani et al. [33], Shu et al. [35], Smulevich et al. [39], UKCCS Investigators. [42], Hardell et al. [14], Altinkaynak et al. [16], Bener et al. [51], Rudant et al. [55] |
| B.3 | Davis et al. [15], Magnani et al. [33], Shu et al. [35], Smulevich et al. [39] |
| B.4 | UKCCS Investigators. [42], Hardell et al. [14], Altinkaynak et al. [16], Petridou et al. [50], Bener et al. [51], Rudant et al. [55], Amitay et al. [64] |
| B.5 | Magnani et al. [33], Smulevich et al. [39], UKCCS Investigators. [42], Hardell et al. [14], Petridou et al. [50], Rudant et al. [55] |
| B.6 | Shu et al. [35], Altinkaynak et al. [16], Bener et al. [51], Amitay et al. [64] |
| B.7 | Davis et al. [15] |
| B.8 | Magnani et al. [33], Bener et al. [51] |
| B.9 | Davis et al. [15], Shu et al. [35], Smulevich et al. [39], UKCCS Investigators. [42], Hardell et al. [14], Altinkaynak et al. [16], Petridou et al. [50], Rudant et al. [55], Amitay et al. [64] |
| B.10 | Shu et al. [35], Hardell et al. [14], Altinkaynak et al. [16], Petridou et al. [50], Bener et al. [51] |
| B.11 | Davis et al. [15], Magnani et al. [33], Smulevich et al. [39], UKCCS Investigators. [42], Rudant et al. [55], Amitay et al. [64] |
| B.12 | Davis et al. [15], Shu et al. [35], Smulevich et al. [39], UKCCS Investigators. [42], Hardell et al. [14], Altinkaynak et al. [16], Rudant et al. [55], Amitay et al. [64] |
| B.13 | Magnani et al. [33], Petridou et al. [50], Bener et al. [51] |
| B.14 | Davis et al. [15], Magnani et al. [33], Shu et al. [35], UKCCS Investigators. [42], Petridou et al. [50], Rudant et al. [55] |
| B.15 | Smulevich et al. [39], Hardell et al. [14], Altinkaynak et al. [16], Bener et al. [51], Amitay et al. [64] |
| B.16 | Smulevich et al. [39], Hardell et al. [14] |
| B.17 | Altinkaynak et al. [16], Bener et al. [51], Amitay et al. [64] |
| C.1 | Hardell et al. [14], Schüz et al. [43], Shaw et al. [49], Bailey et al. [66] |
| C.2 | Hardell et al. [14], Schüz et al. [43], Shaw et al. [49], Bailey et al. [66] |
| C.3 | Hardell et al. [14], Schüz et al. [43] |
| C.4 | Hardell et al. [14], Shaw et al. [49], Bailey et al. [66] |
| C.5 | Davis et al. [15], Smulevich et al. [39], Hardell et al. [14], Schüz et al. [43] |
| C.6 | Shaw et al. [49], Greenop et al. [62], Bailey et al. [66] |
| C.7 | Davis et al. [15], Shaw et al. [49] |
| C.8 | Smulevich et al. [39], Hardell et al. [14], Schüz et al. [43], Bailey et al. [66] |
| C.9 | Greenop et al. [62] |
| C.10 | Davis et al. [15], Smulevich et al. [39], Schüz et al. [43], Shaw et al. [49], Greenop et al. [62] |
| C.11 | Hardell et al. [14], Bailey et al. [66] |
| C.12 | Davis et al. [15], Smulevich et al. [39], Hardell et al. [14], Shaw et al. [49] |
| C.13 | Schüz et al. [43], Greenop et al. [62], Bailey et al. [66] |
| C.14 | Shaw et al. [49], Greenop et al. [62], Bailey et al. [66] |
| C.15 | Davis et al. [15], Smulevich et al. [39], Hardell et al. [14], Schüz et al. [43] |
| C.16 | Smulevich et al. [39], Hardell et al. [14], Schüz et al. [43] |
| C.17 | Davis et al. [15] |
|  |  |

**
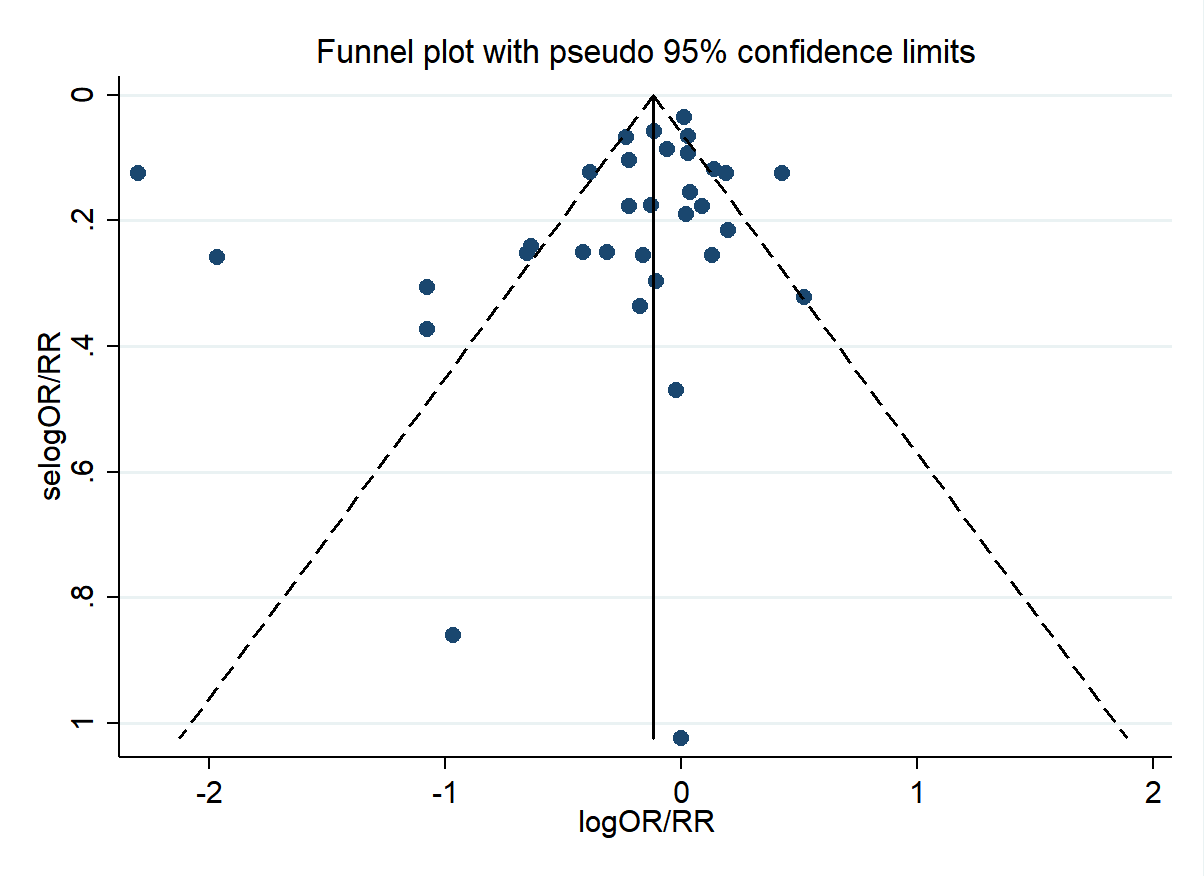
Fig. S2** Begg’s funnel plots identifying the publication bias for the association between breastfeeding and risk of childhood leukemia.

**(A.1)**


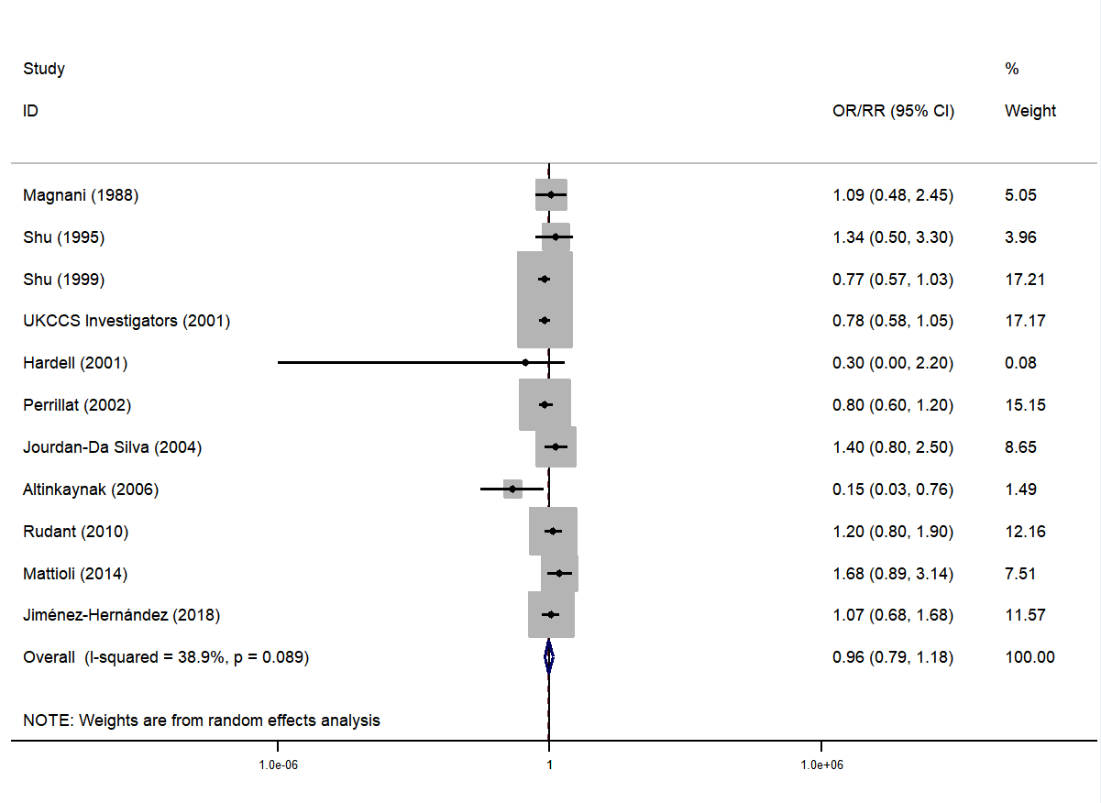


**(A.2)**


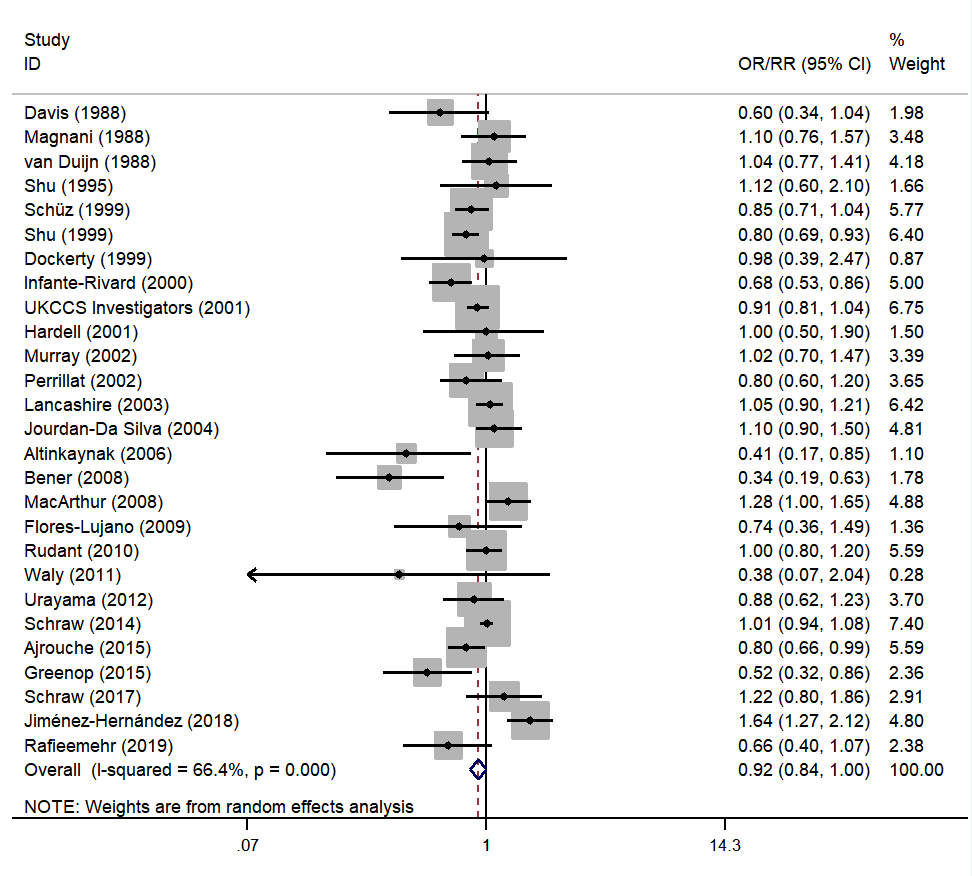


**(A.3)**


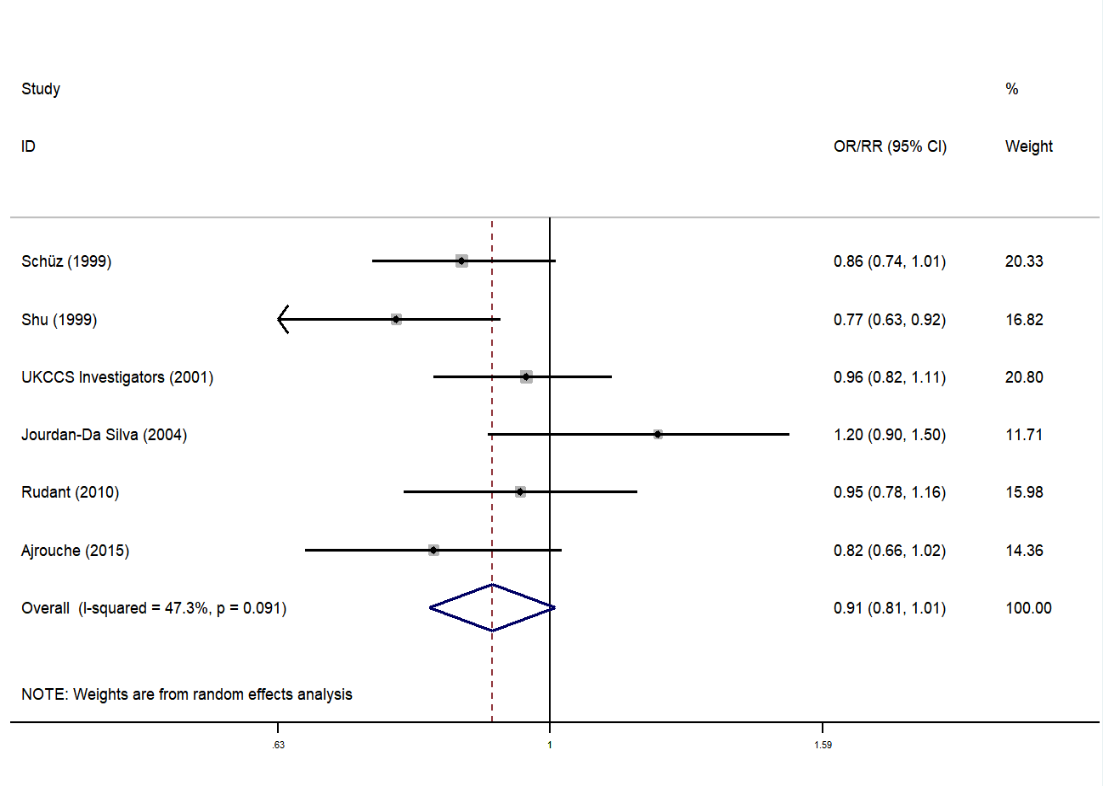


**(A.4)**


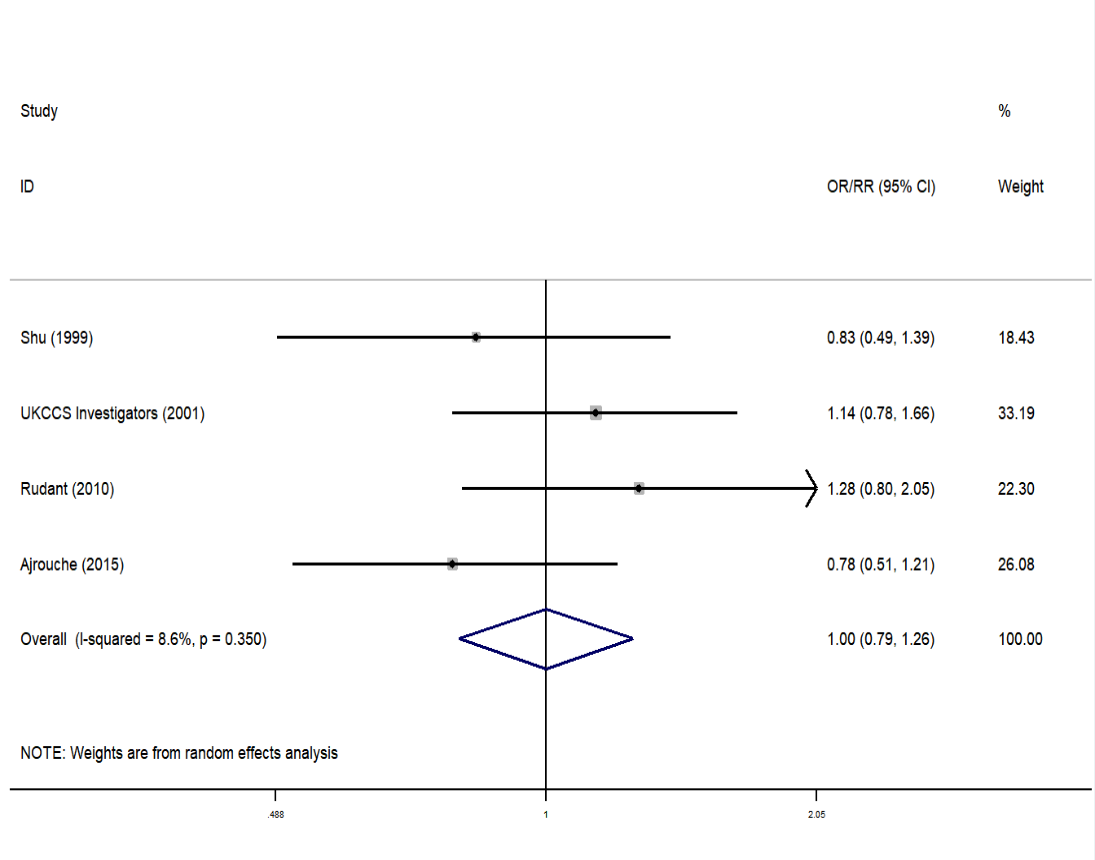


**(A.5)**

**
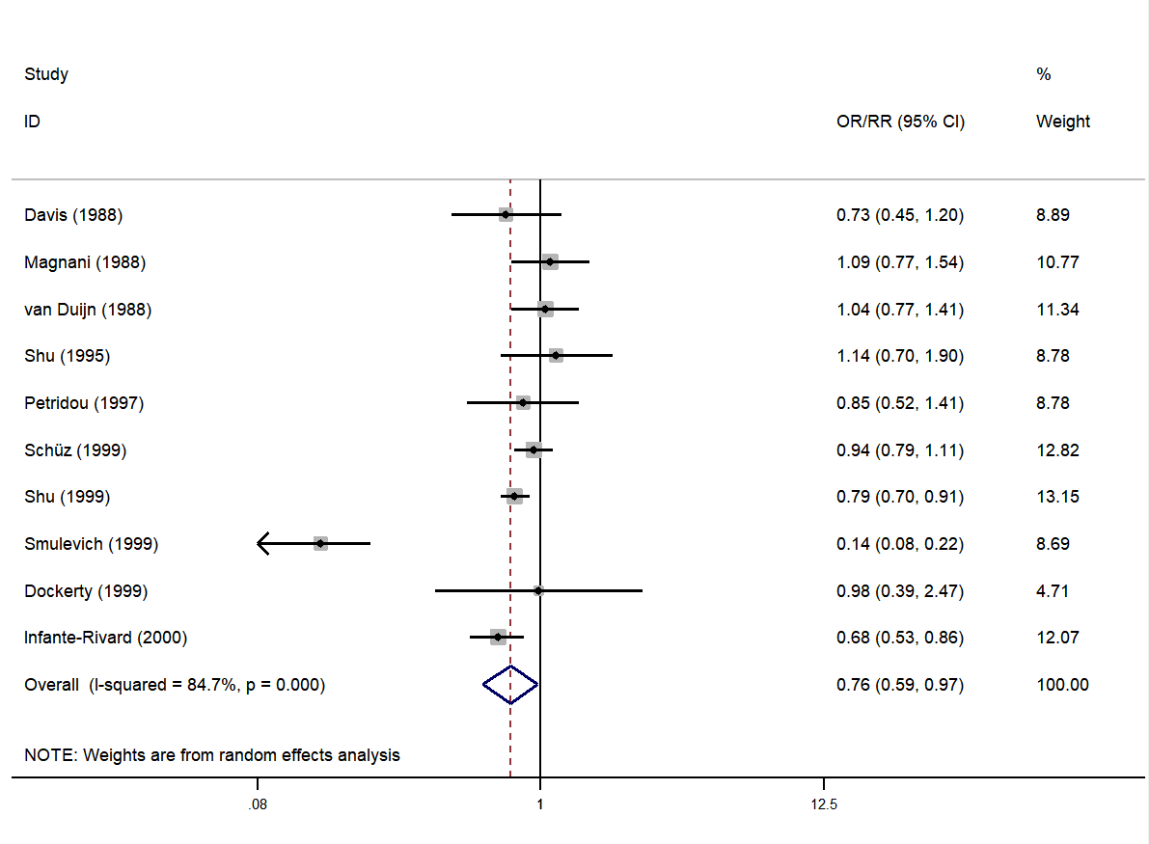
**

**(A.6)**


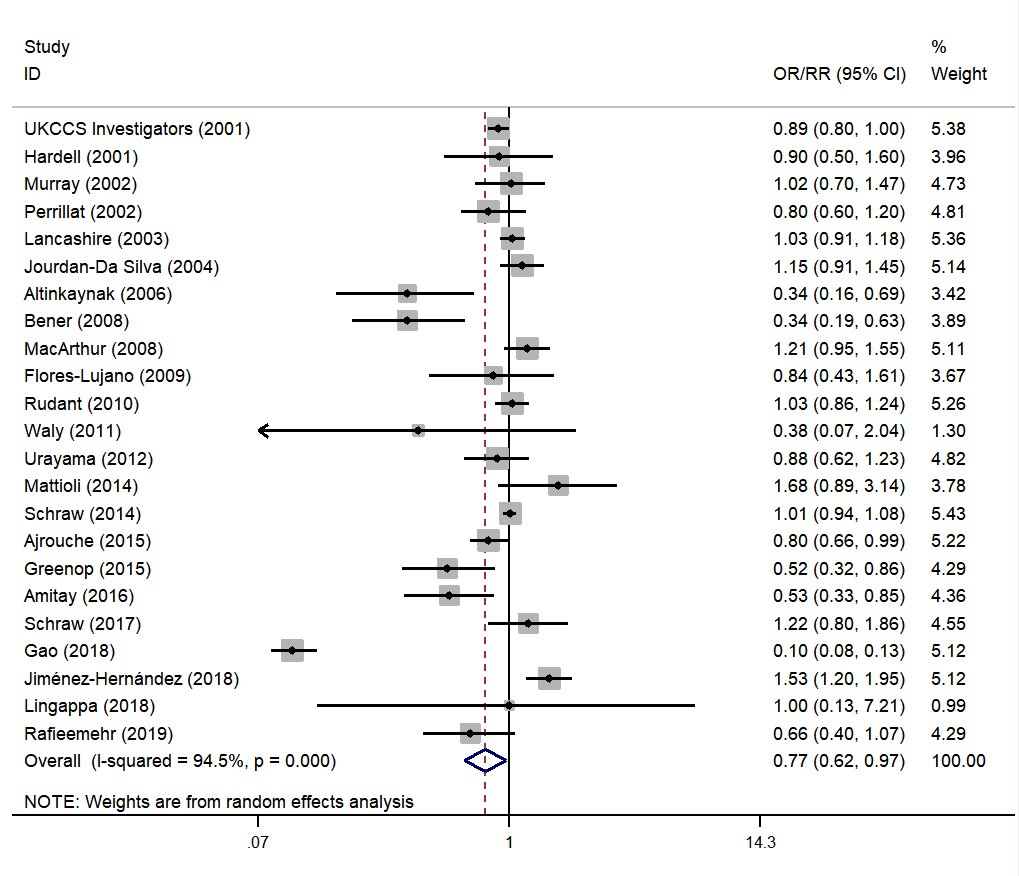


**(A.7)**

**
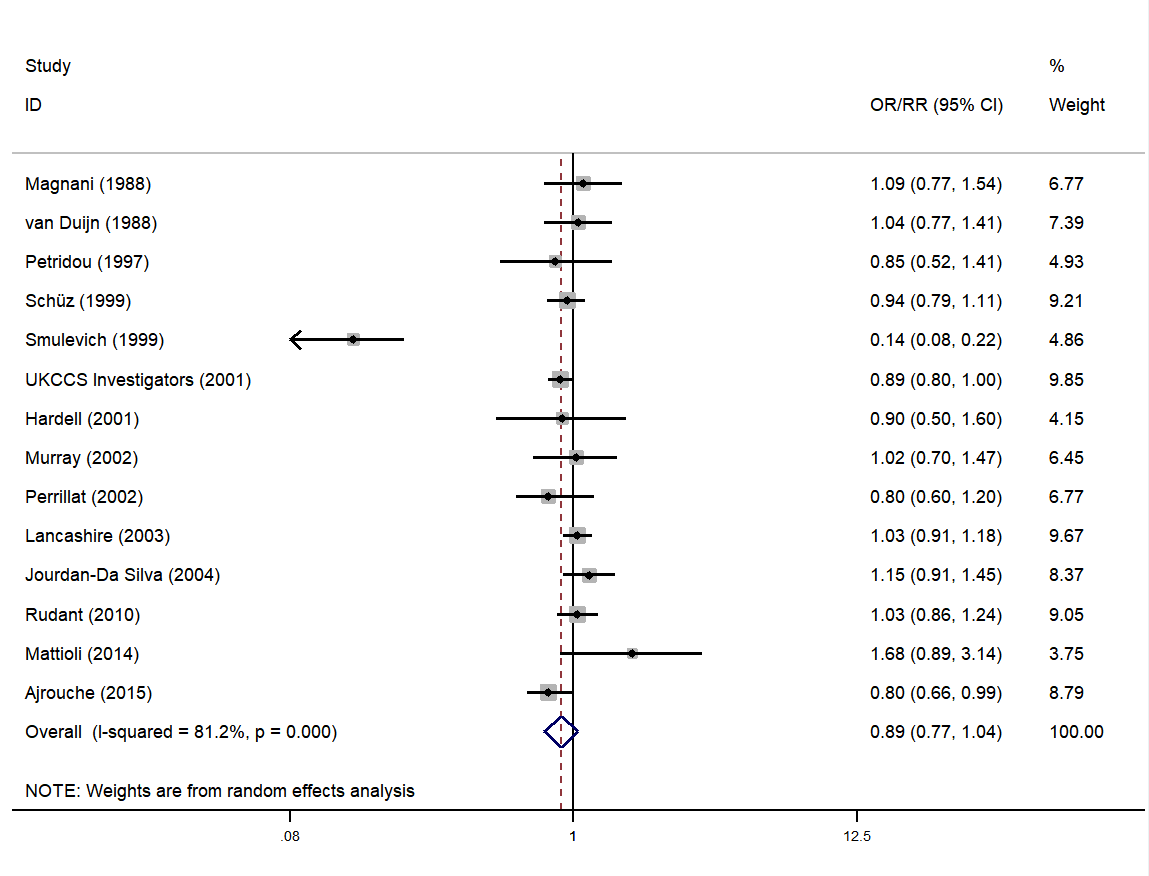
**

**(A.8)**

**
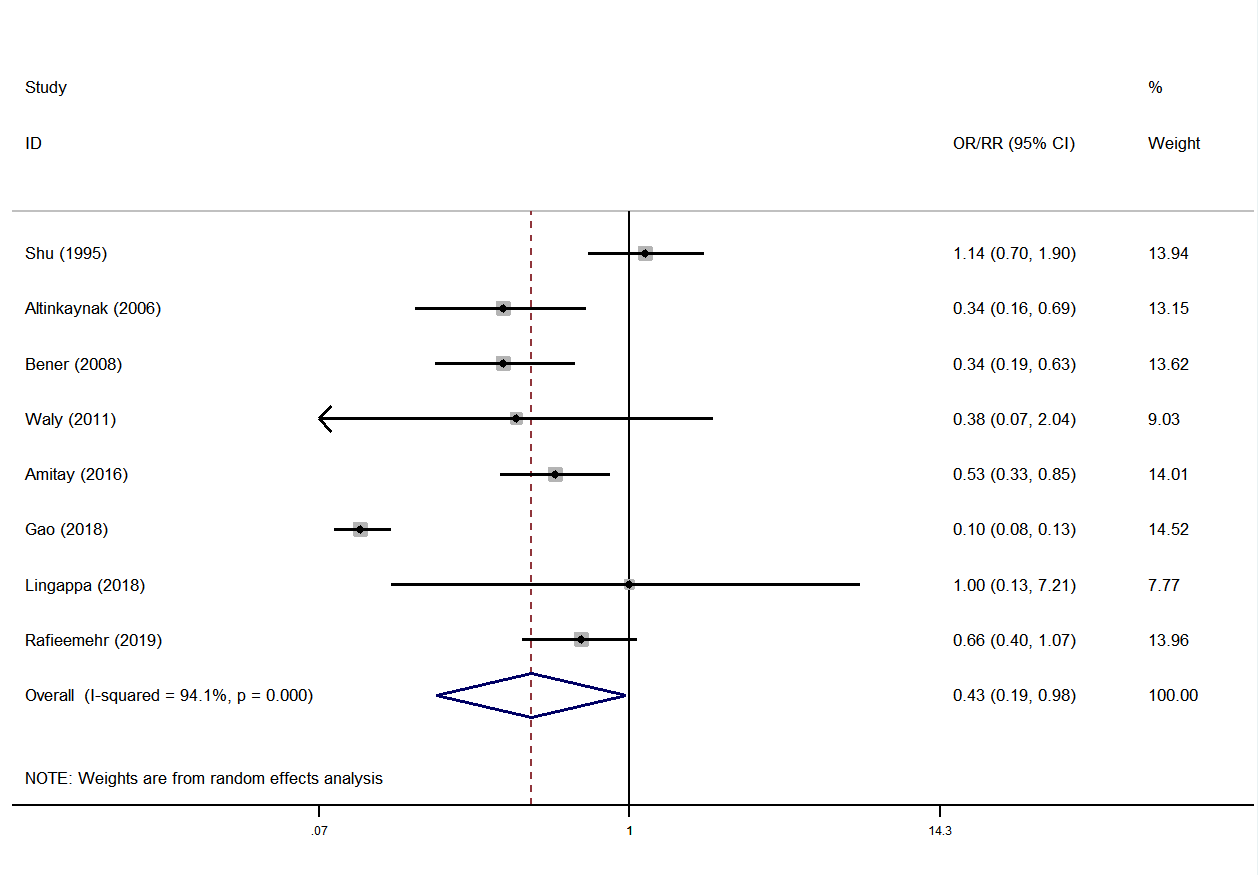
**

**(A.9)**

**
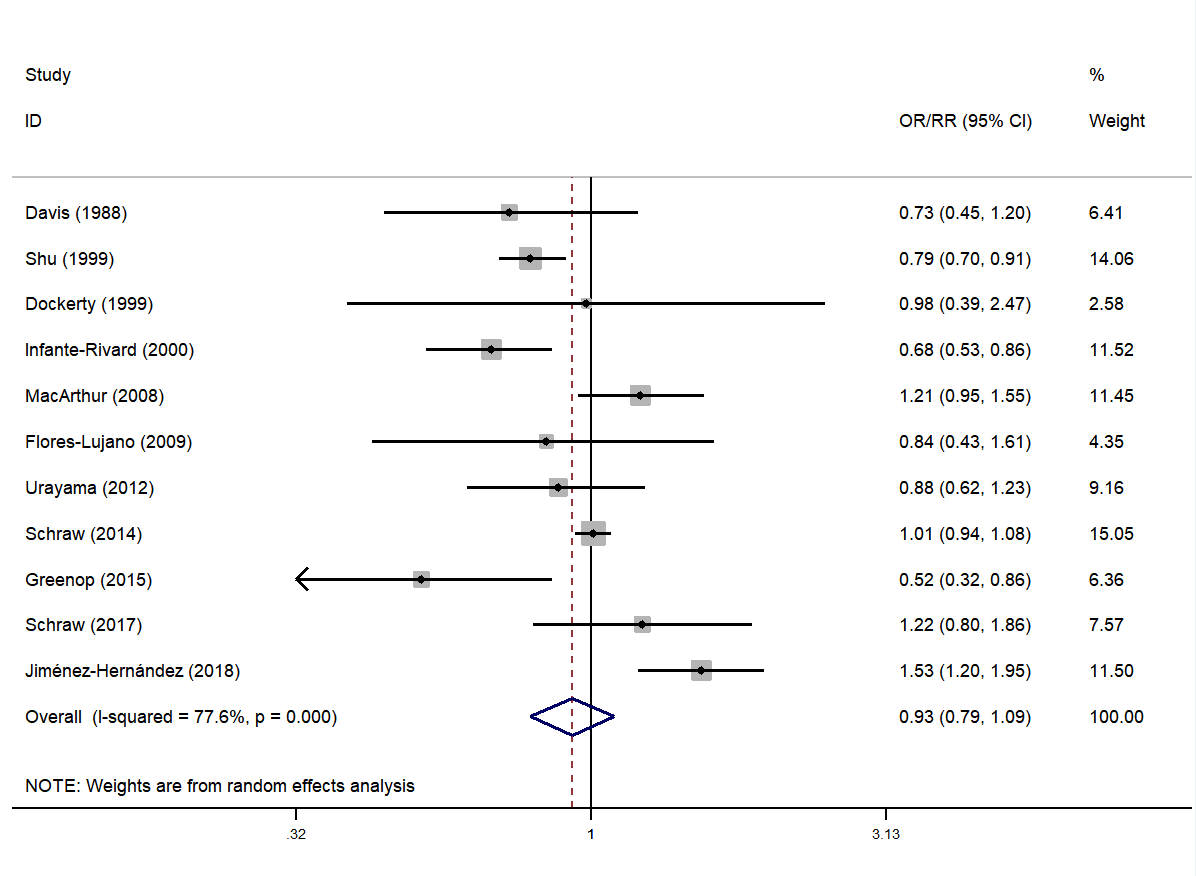
**

**(A.10)**

**
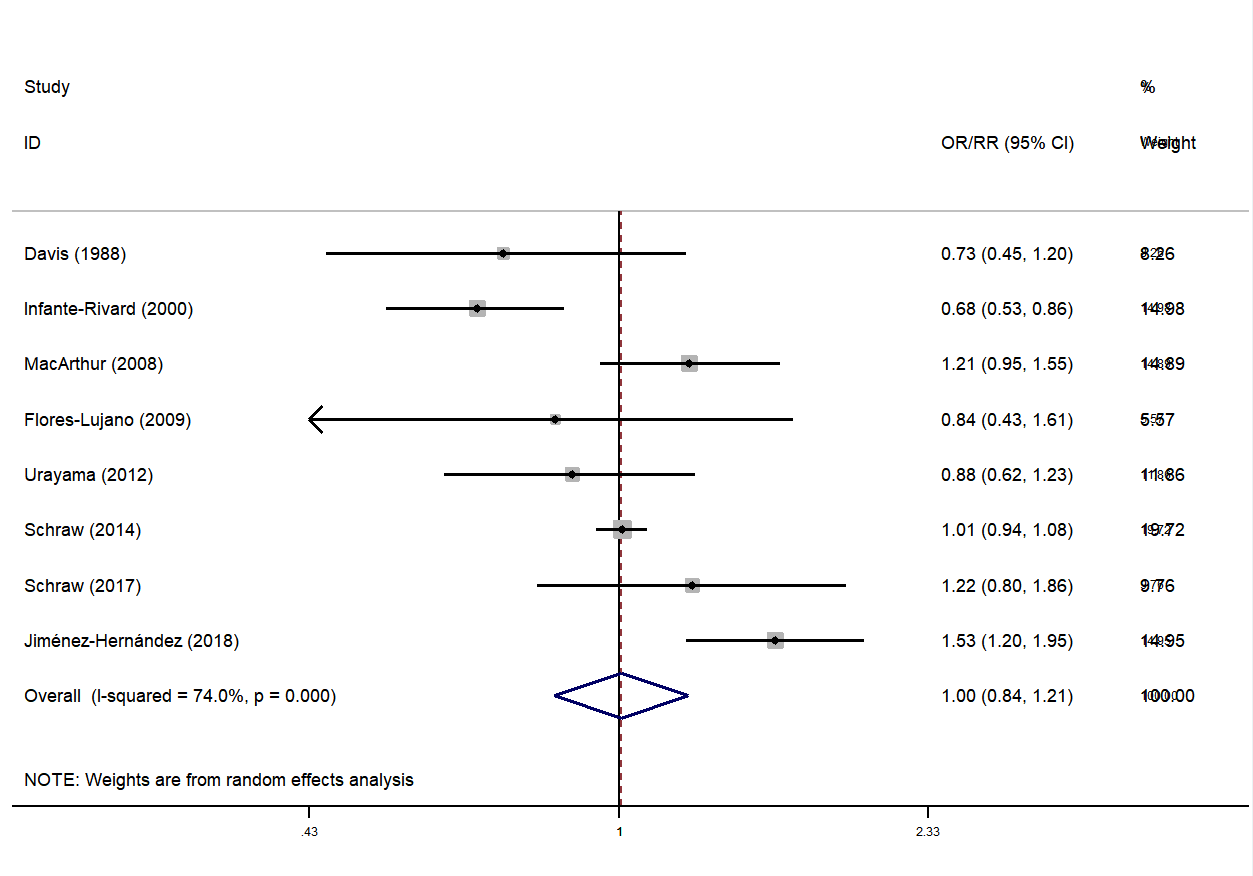
**

**(A.11)**

**
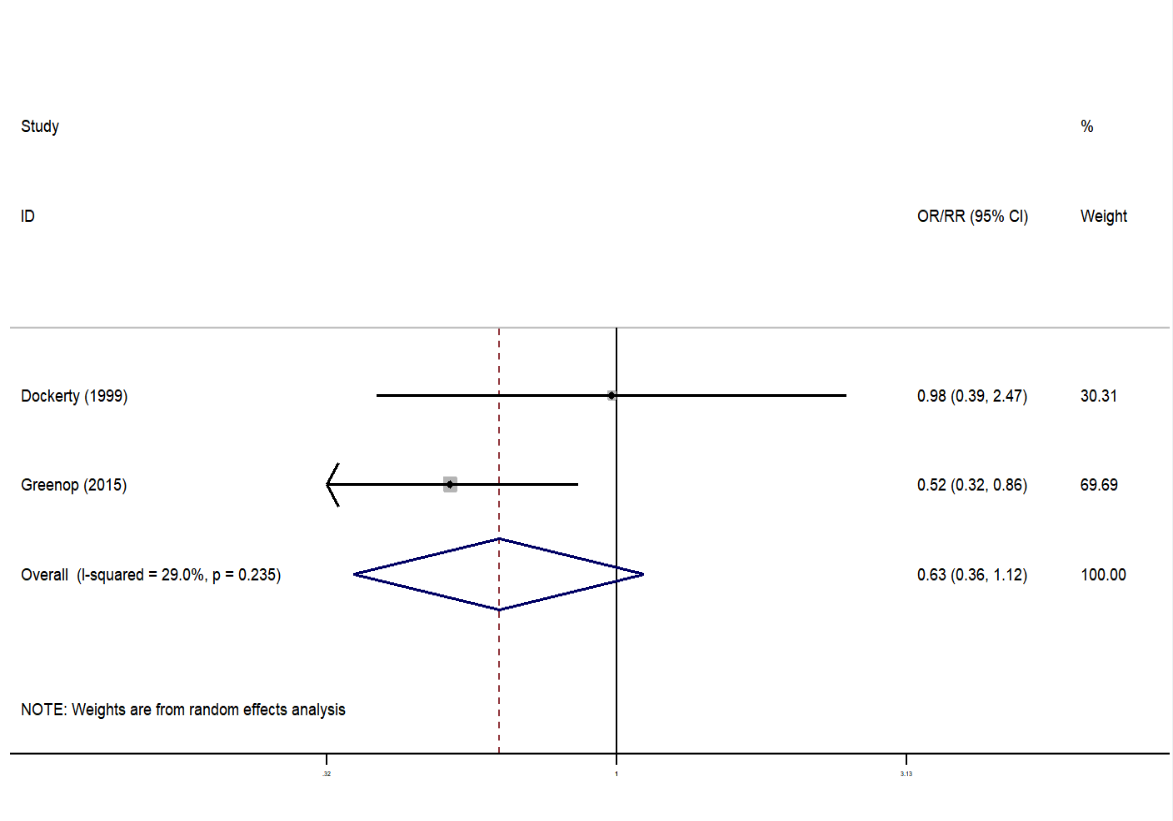
**

**(A.12)**

**
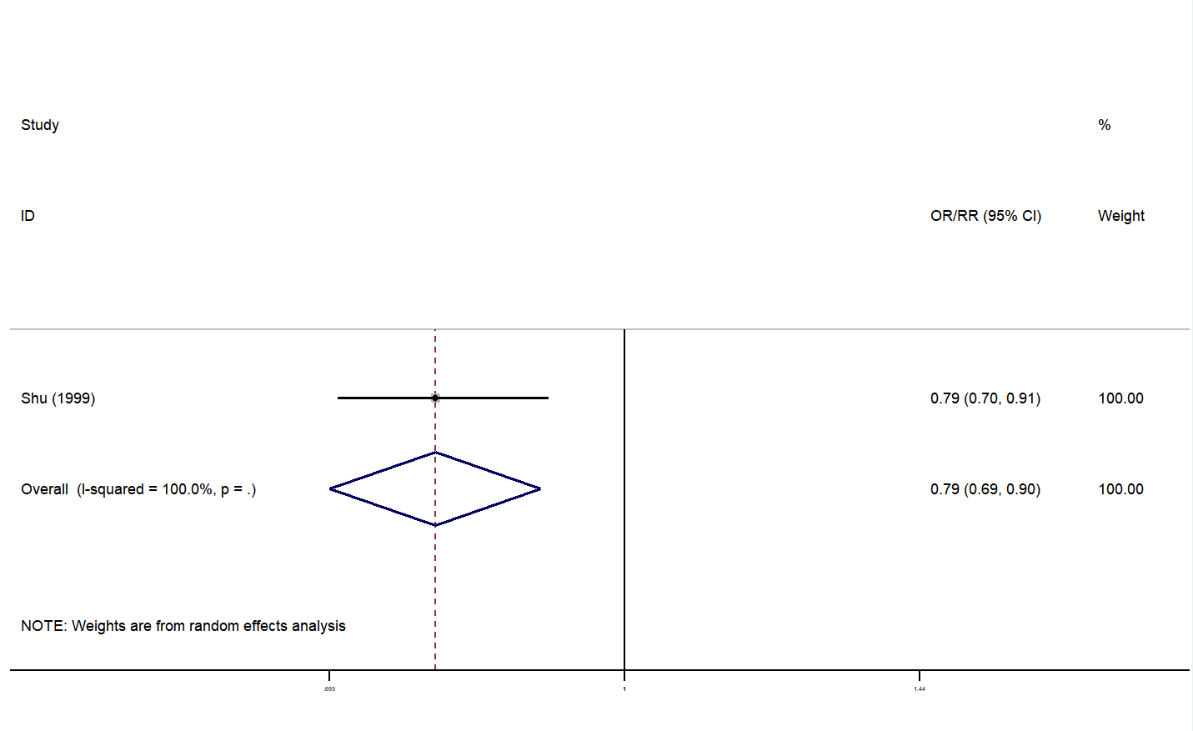
**

**(A.13)**

**
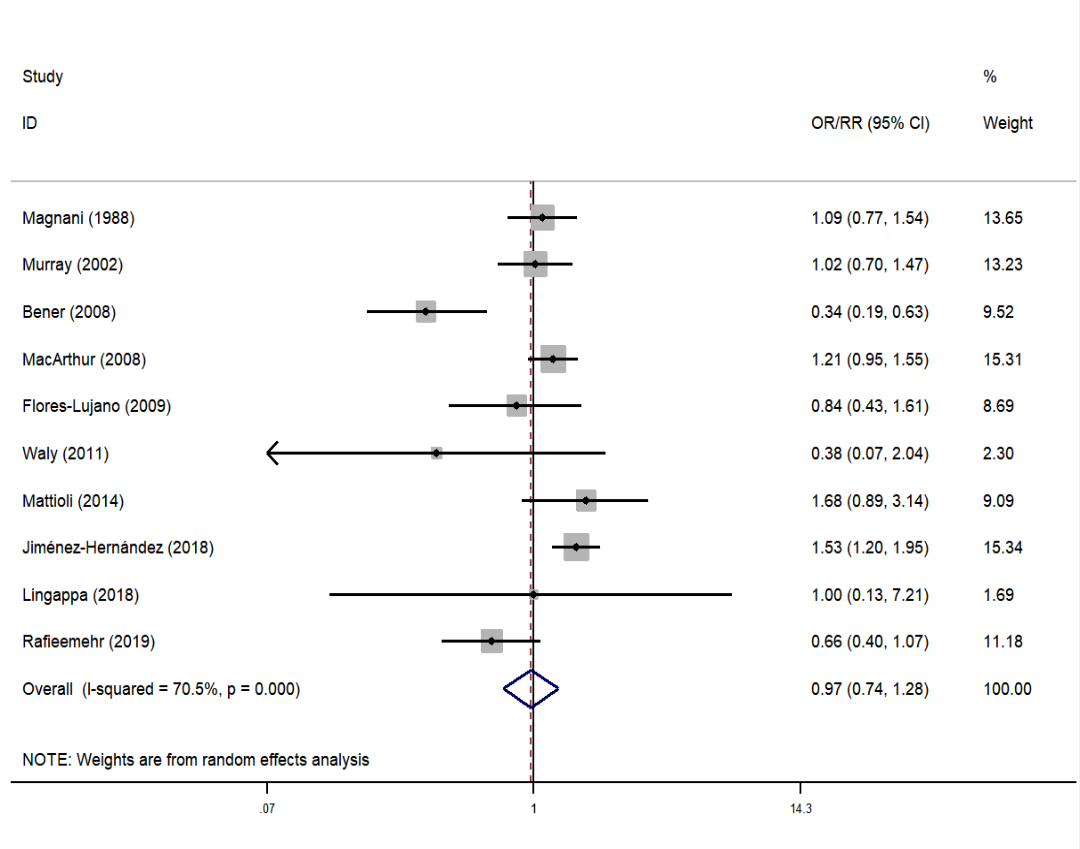
**

**(A.14)**

**
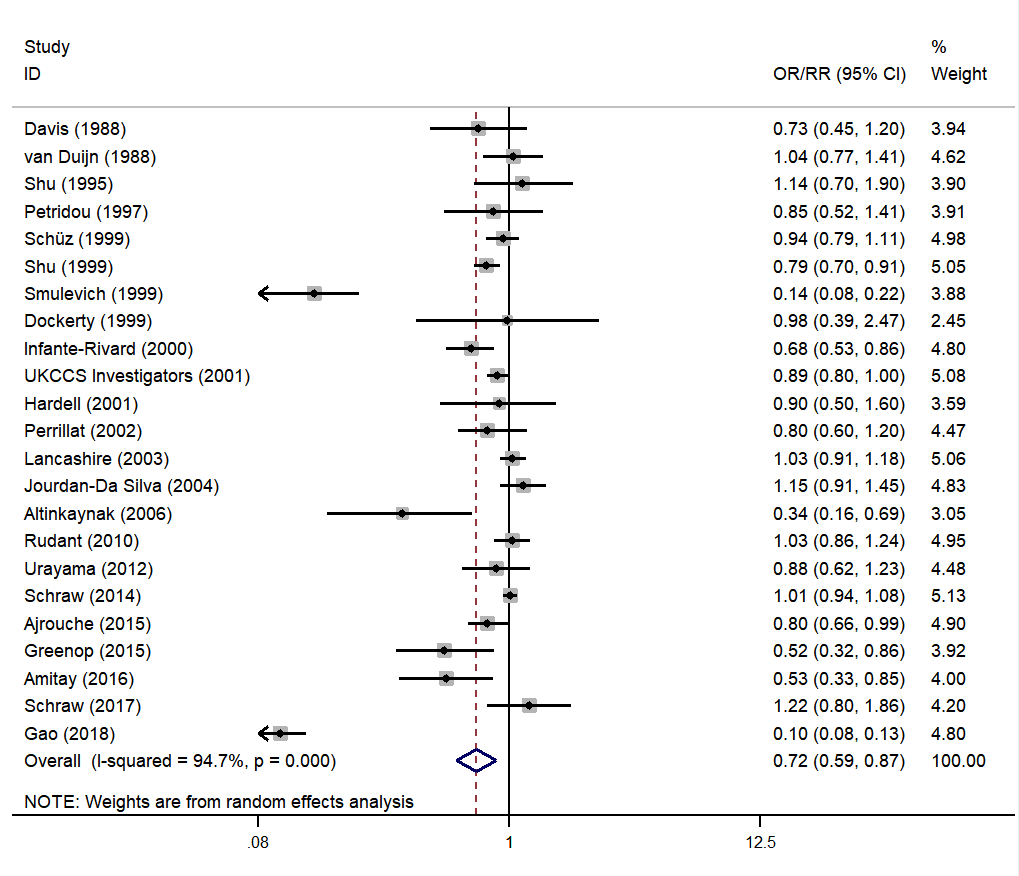
**

**(A.15)**

**
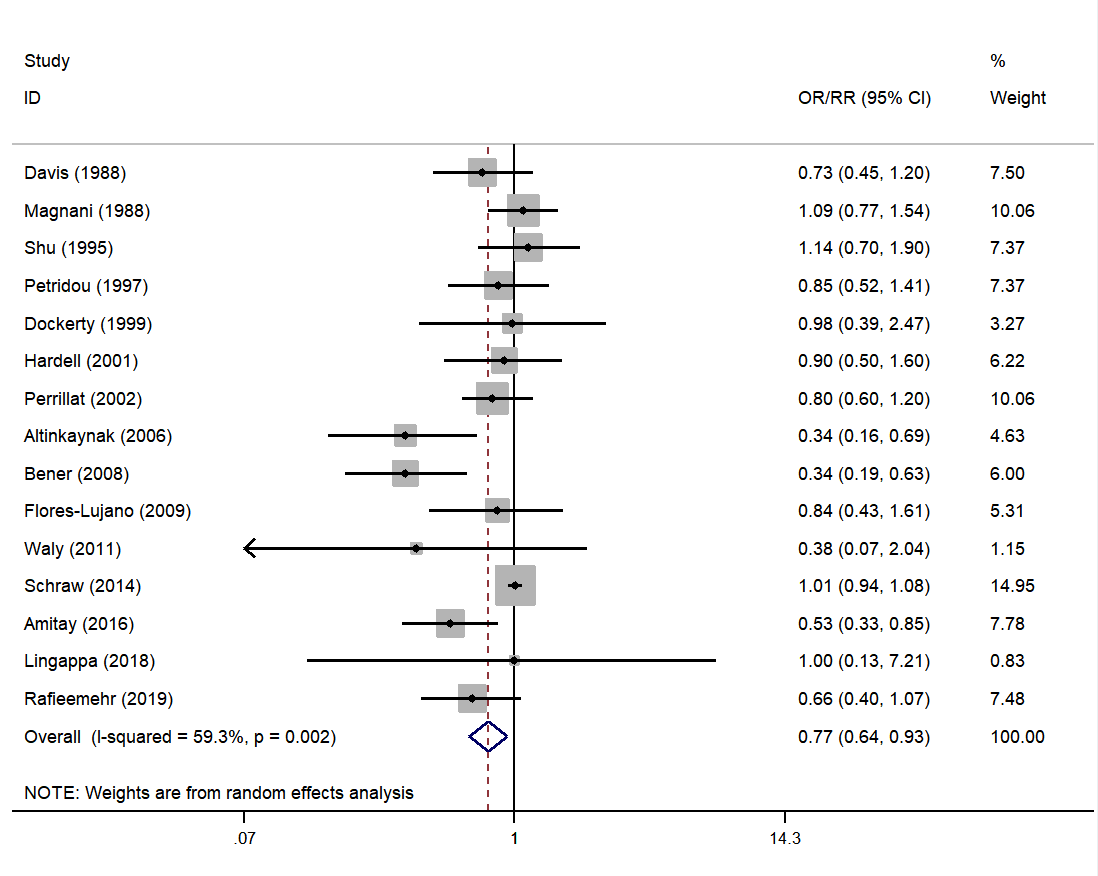
**

**(A.16)**

**
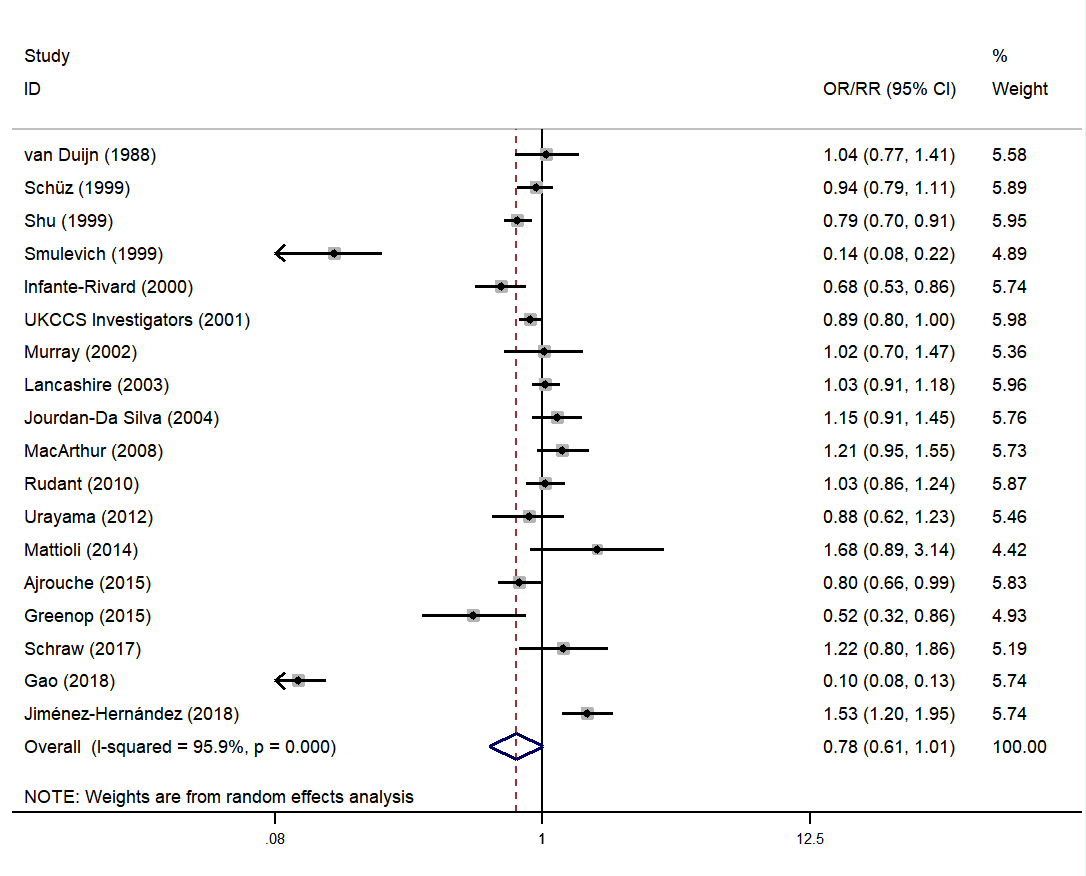
**

**(A.17)**

**
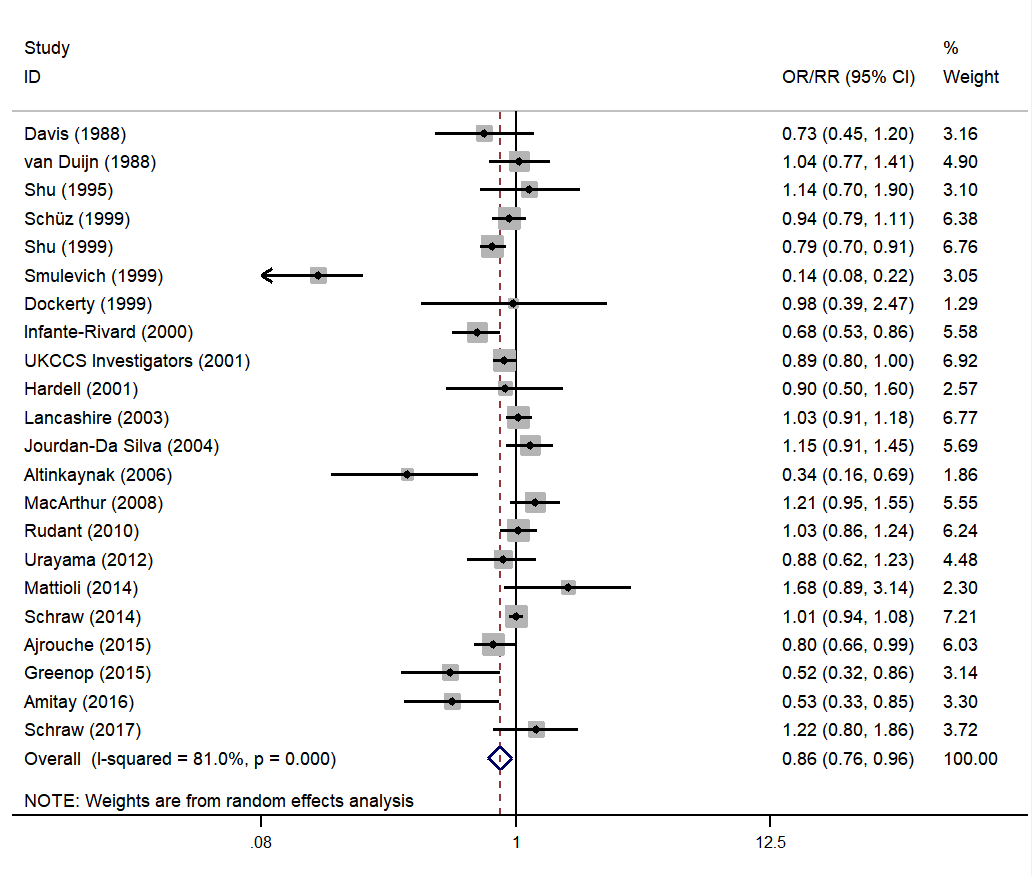
**

**(A.18)**

**
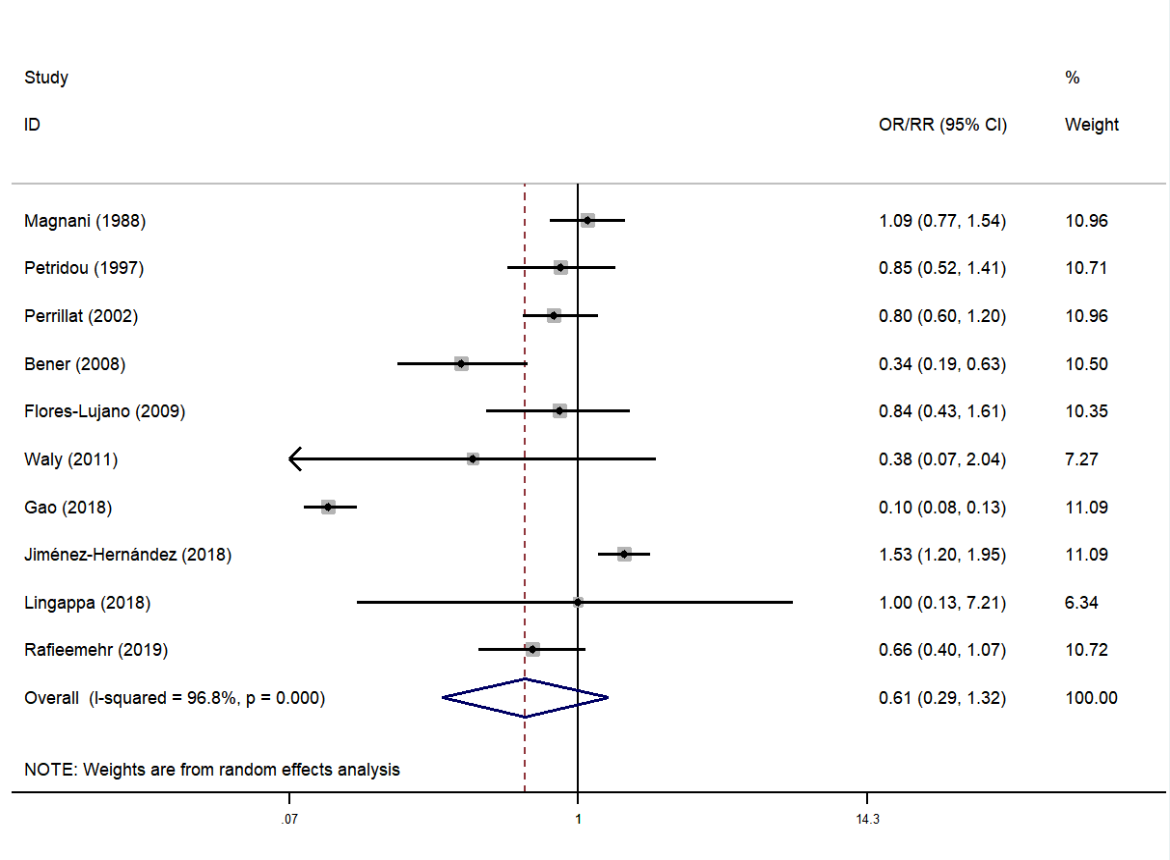
**

**(A.19)**

**
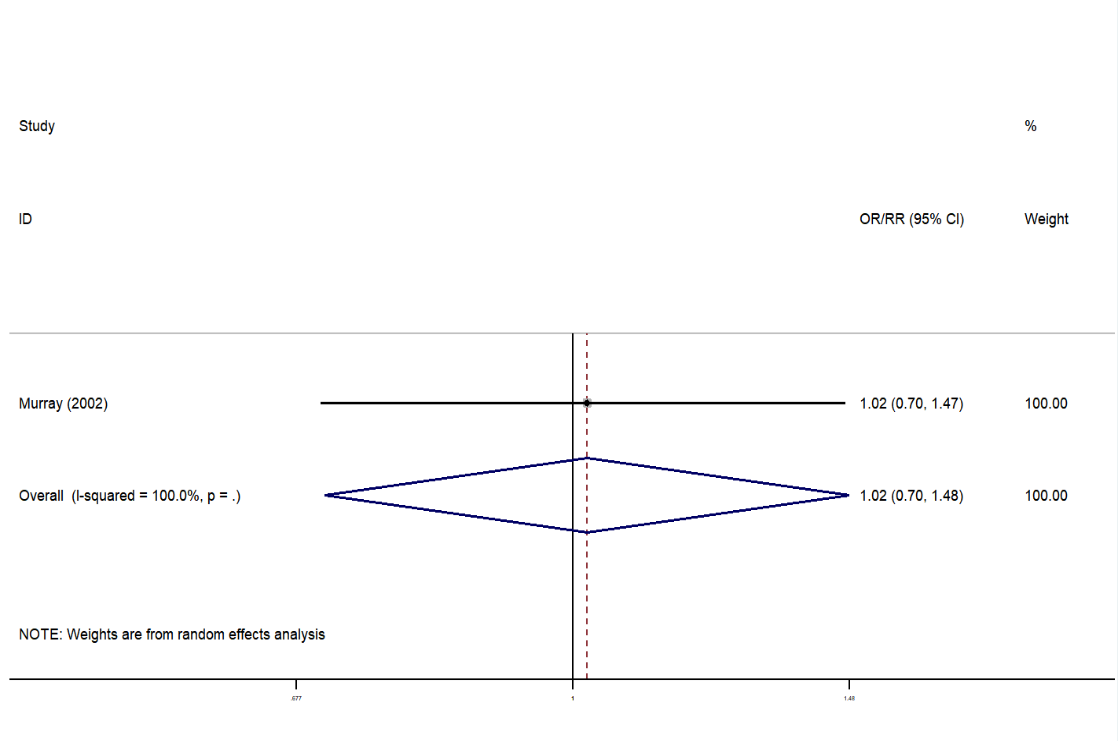
**

**(A.20)**

**
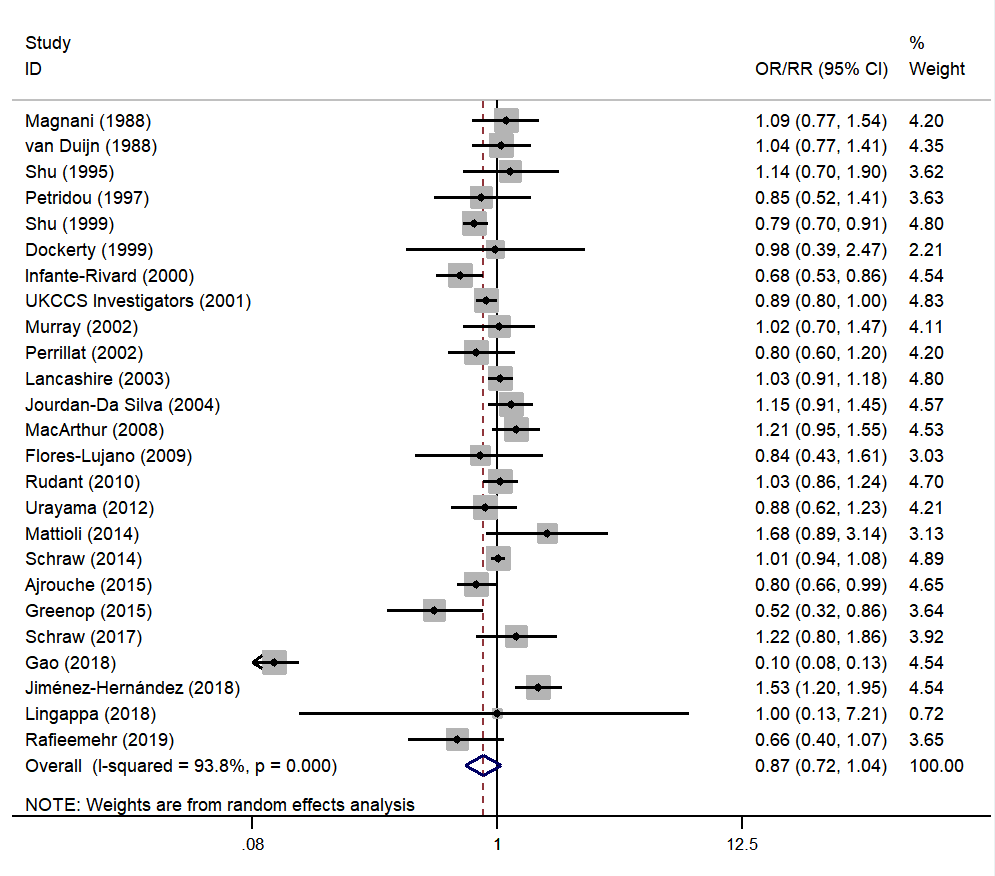
**

**(A.21)**

**
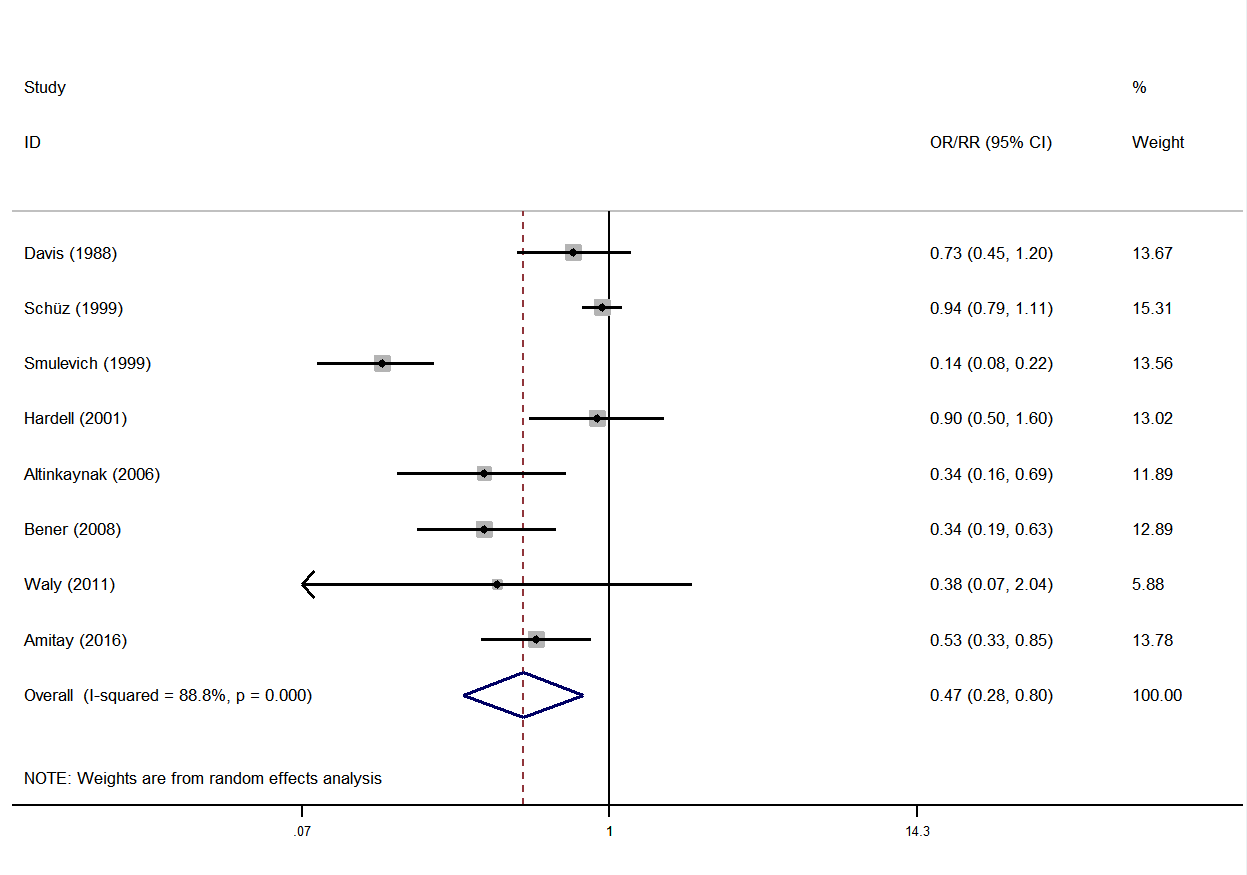
**

**(A.22)**

**
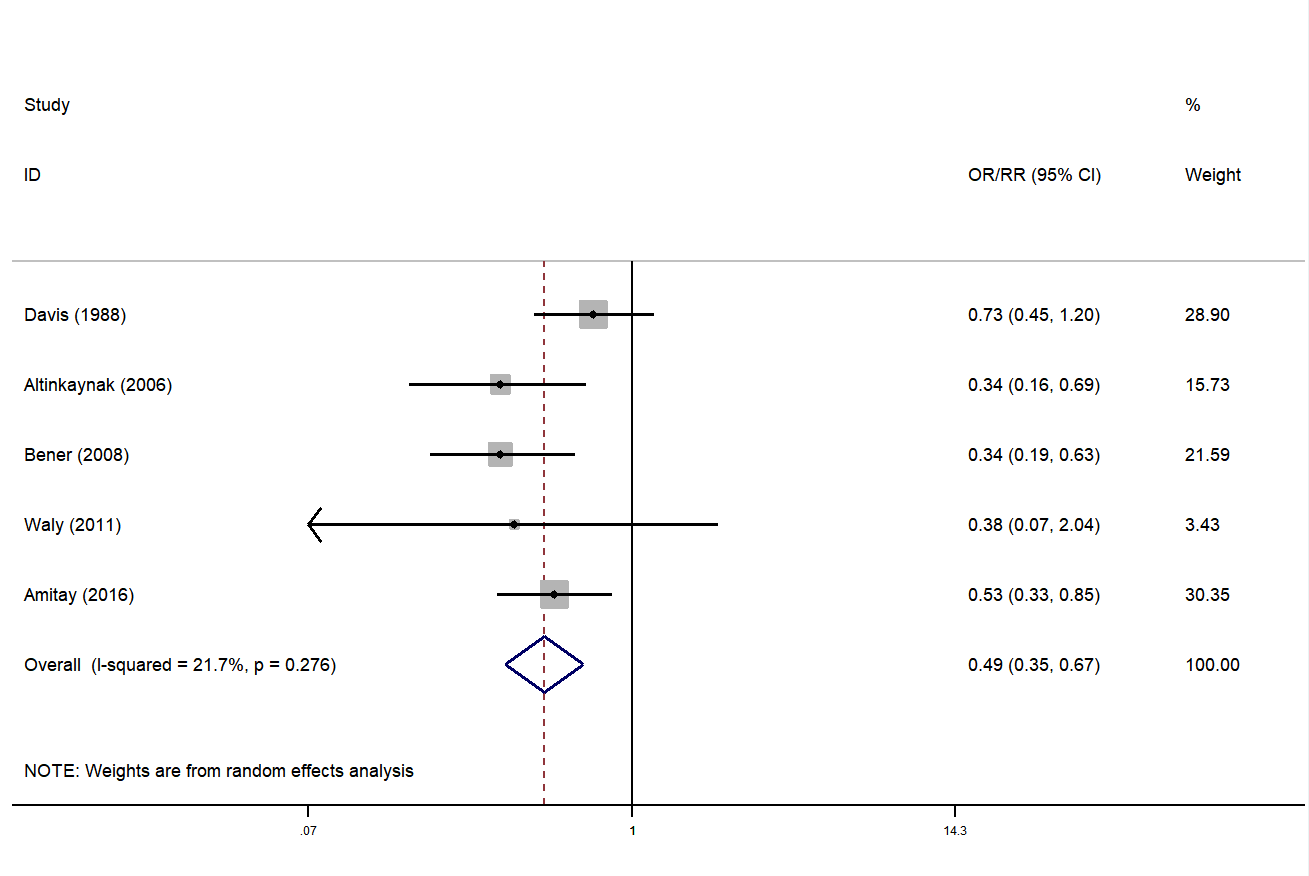
**

**(A.23)**

**
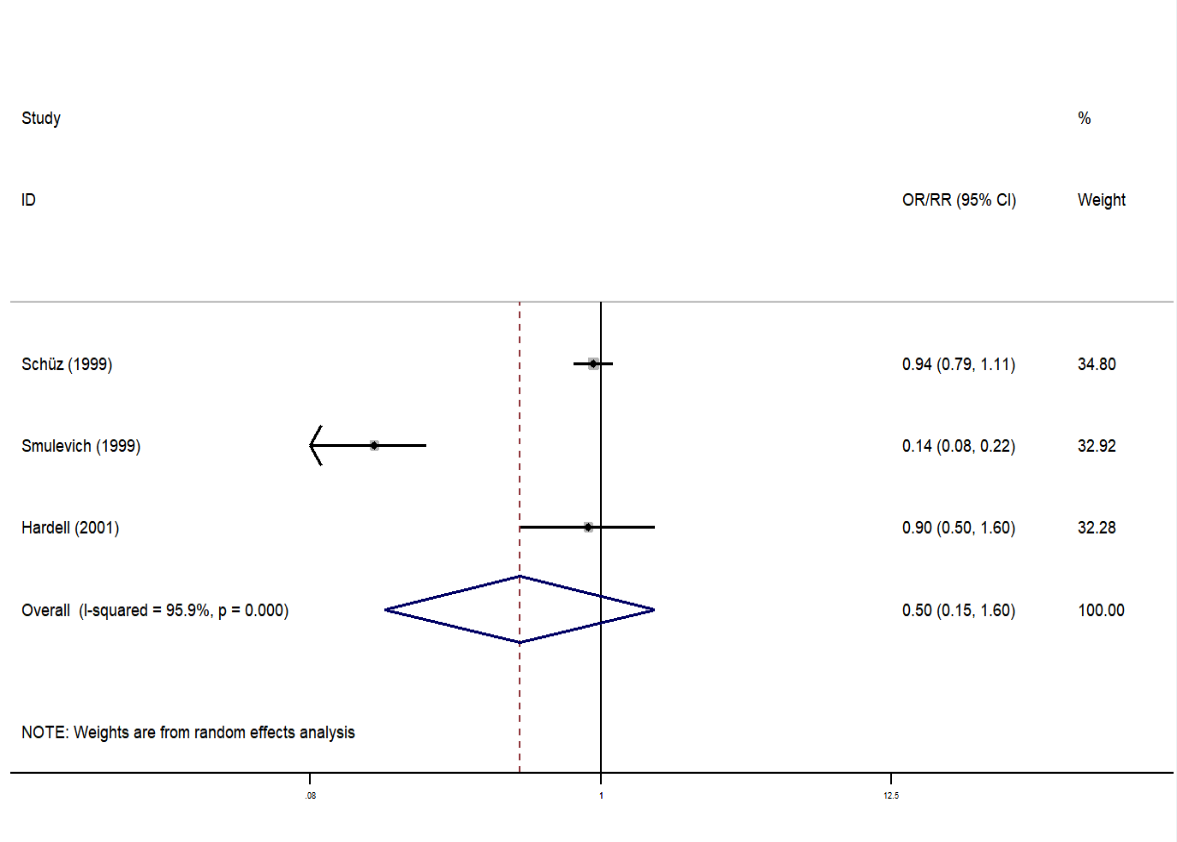
**

**Fig. S3** Forest plots of subgroup analysis of association between breastfeeding and childhood leukemia risk in the order listed in Table 2.

| **Table S4.** **Subgroup analysis of the association between breastfeeding and acute lymphoblastic leukemia risk** | | | | | | |
| --- | --- | --- | --- | --- | --- | --- |
|  | No. of studies | OR (95%CI) | P value | I^2^ (%) | *P* value for heterogeneity | *P* in meta- regression |
| Year of publication |  |  |  |  |  | 0.550 |
| Before 2000 | 8 | 0.84 (0.75~0.95) | 0.006 | 27.4 | 0.209 |  |
| In and after 2000 | 19 | 0.94 (0.84~1.05) | 0.276 | 68.6 | <0.001 |  |
| Geographic location |  |  |  |  |  | 0.164 |
| Europe | 11 | 0.95 (0.89~1.01) | 0.103 | 0.0 | 0.453 |  |
| Asia | 5 | 0.56 (0.35~0.90) | 0.016 | 53.0 | 0.075 |  |
| North America or Oceania | 11 | 0.93 (0.78~1.11) | 0.408 | 79.1 | <0.001 | 0.337 |
| North America only | 8 | 1.00 (0.81~1.23) | 0.999 | 78.7 | <0.001 |  |
| Oceania only | 2 | 0.63 (0.36~1.12) | 0.113 | 29.0 | 0.235 |  |
| Both^1^ | 1 | 0.80 (0.69~0.93) | 0.003 | NA | NA |  |
| Study quality score |  |  |  |  |  | 0.398 |
| <7 | 8 | 0.91 (0.66~1.26) | 0.565 | 78.4 | <0.001 |  |
| ≥7 | 19 | 0.90 (0.83~0.97) | 0.009 | 53.2 | 0.003 |  |
| Sample size |  |  |  |  |  | 0.127 |
| ≤500 | 12 | 0.78 (0.63~0.96) | 0.021 | 58.1 | 0.006 |  |
| >500 | 15 | 0.96 (0.86~1.07) | 0.468 | 72.4 | <0.001 |  |
| Study design |  |  |  |  |  | 0.935 |
| PC-CS | 19 | 0.92 (0.84~1.00) | 0.049 | 58.4 | 0.001 |  |
| HC-CS | 7 | 0.79 (0.52~1.21) | 0.279 | 82.0 | <0.001 |  |
| Cohort study | 1 | 1.02 (0.70~1.48) | 0.917 | NA | NA |  |
| Definition of reference category |  |  |  |  |  | 0.018 |
| Never breastfeeding | 21 | 0.96 (0.88~1.05) | 0.364 | 64.3 | <0.001 |  |
| Occasional breastfeeding | 6 | 0.61 (0.42~0.89) | 0.010 | 59.9 | 0.029 | 0.033 |
| ≤1 month | 2 | 0.86 (0.72~1.03) | 0.109 | 0.0 | 0.646 |  |
| ≤6 months | 4 | 0.45 (0.31~0.64) | <0.001 | 0.0 | 0.584 |  |

**(A)
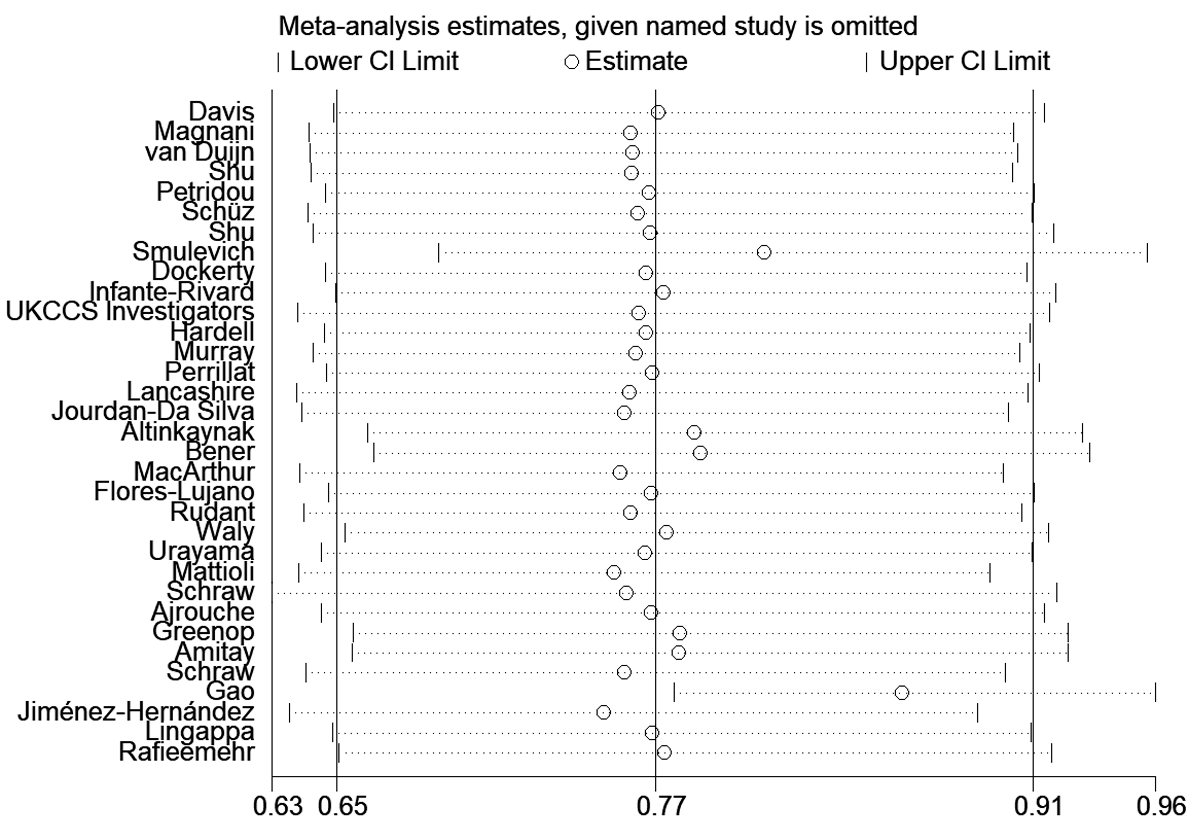
**

**(B)**

**
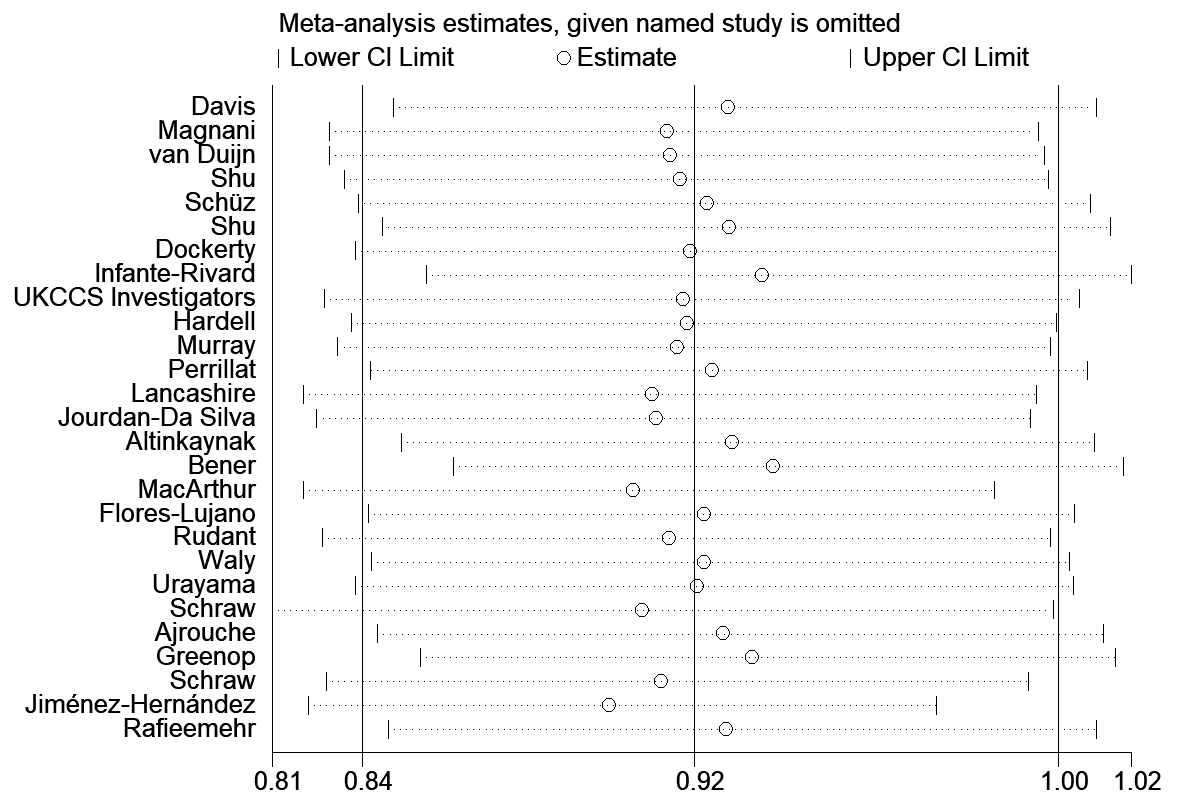
**

**Fig. S4** One-study-removed analysis on the association of breastfeeding with risk of (A) childhood leukemia and (B) acute lymphoblastic leukemia.

**(A)**


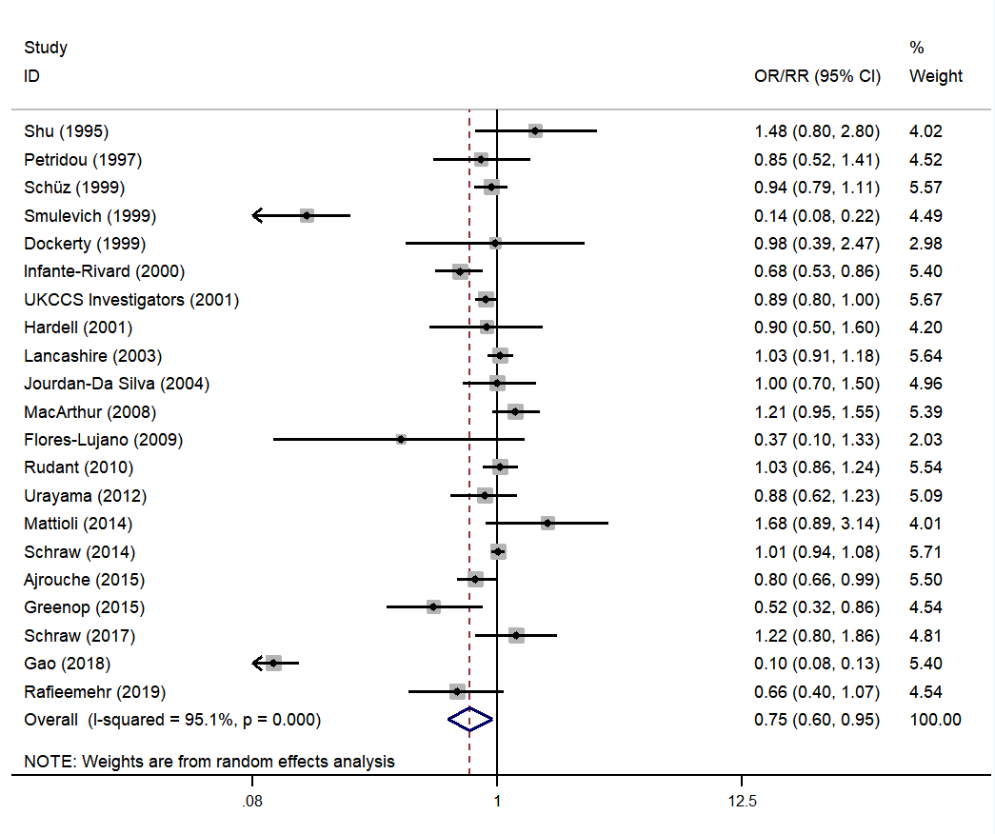


**(B)**


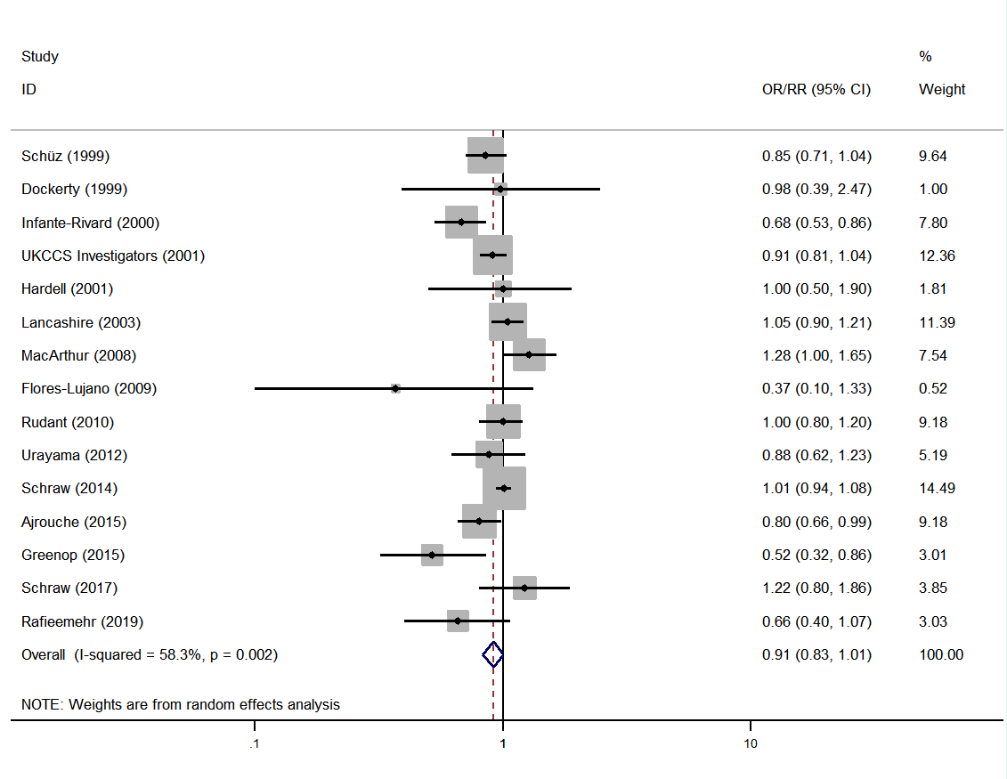


**Fig. S5** Pooled analysis of studies including only children aged 0-14 years old for the for the association of breastfeeding with risk of (A) childhood leukemia and (B) acute lymphoblastic leukemia.


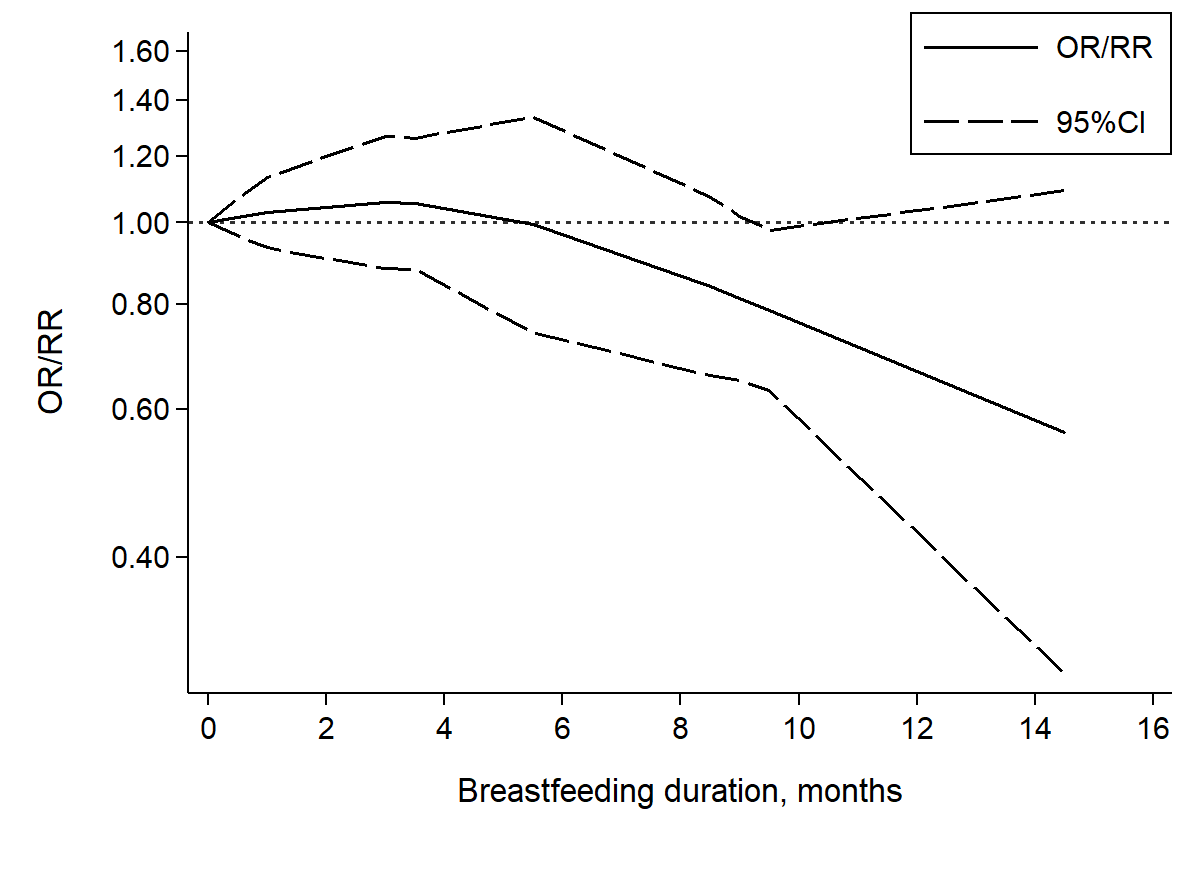


**Fig. S6** Risk estimates (solid line) and the corresponding 95% CIs (dash lines) for the dose-response relationship between breastfeeding and the risk of childhood lymphoma.


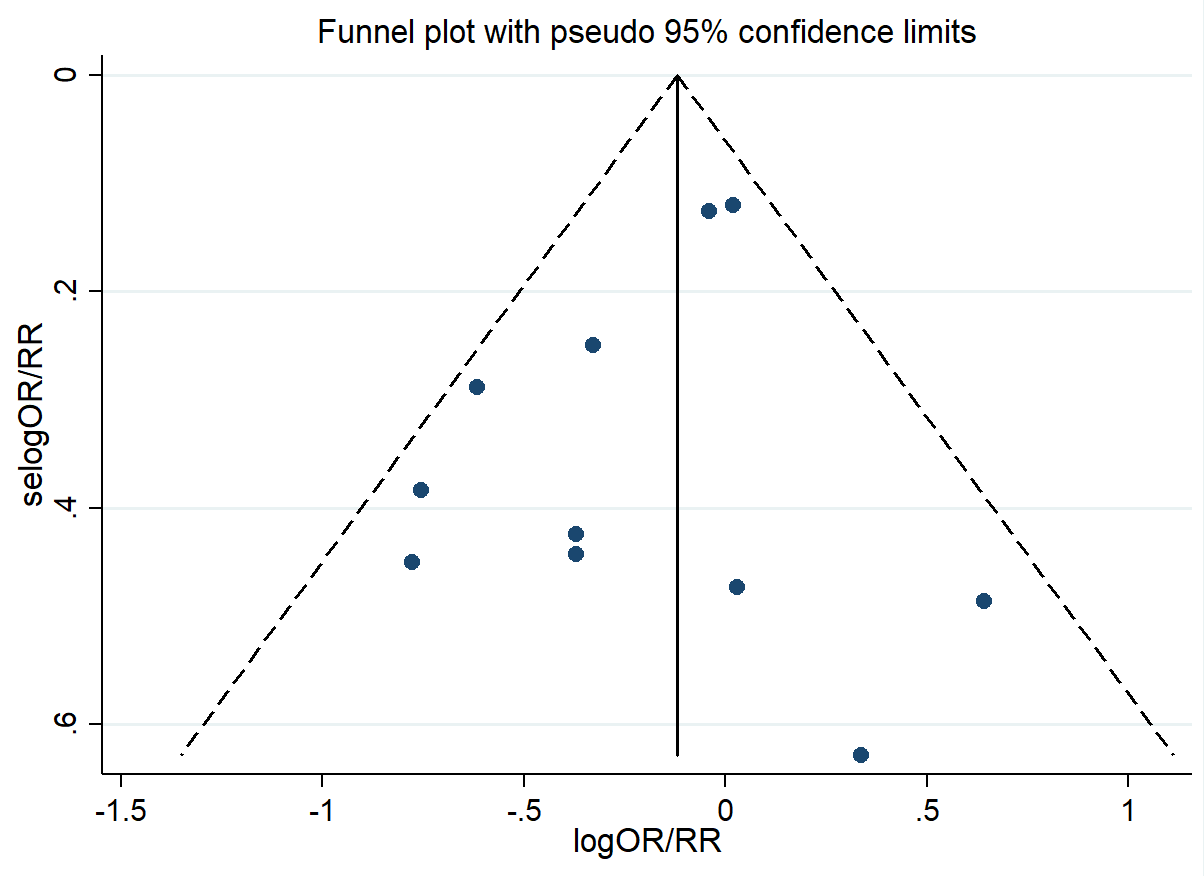


**Fig. S7** Begg’s funnel plots identifying the publication bias for the association between breastfeeding and risk of childhood lymphoma.

**(B.1)**

**
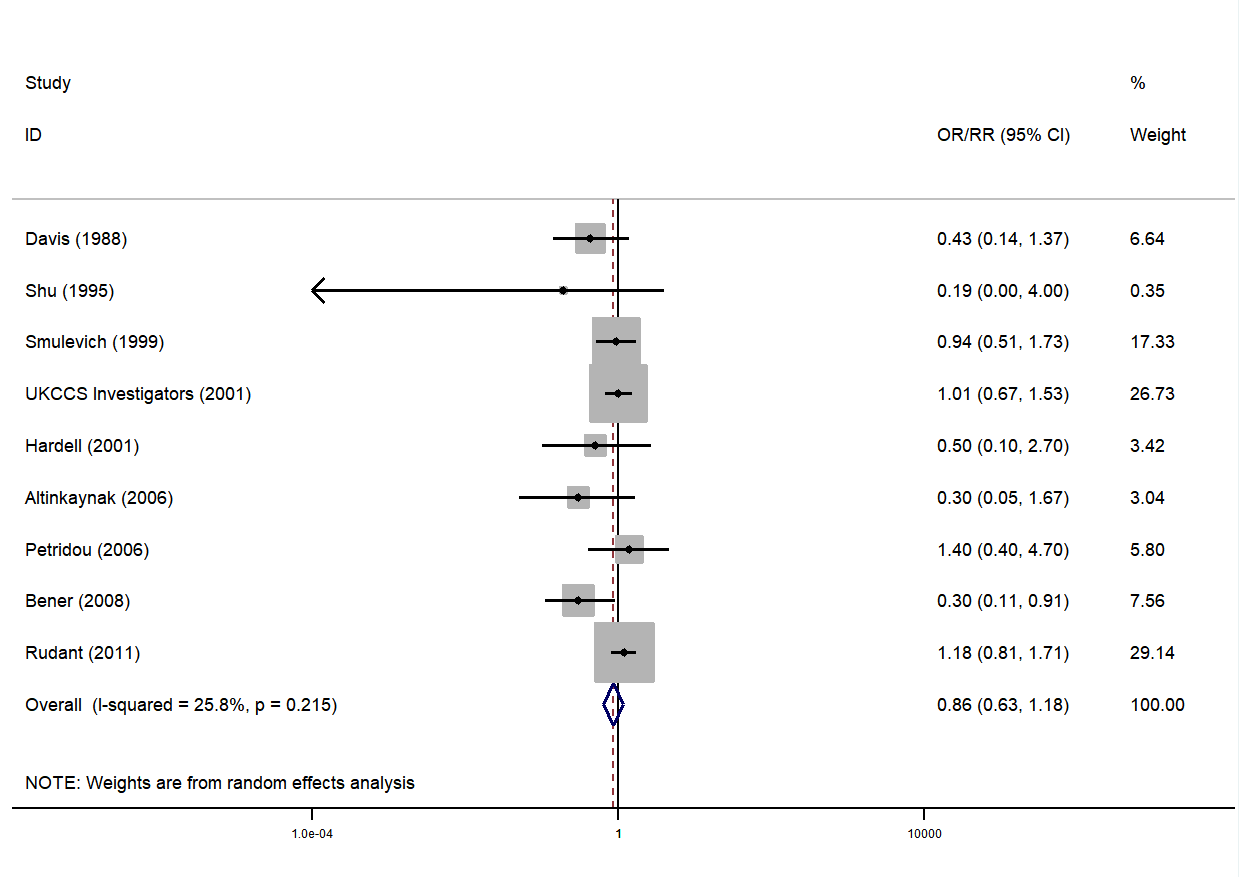
**

**(B.2)**


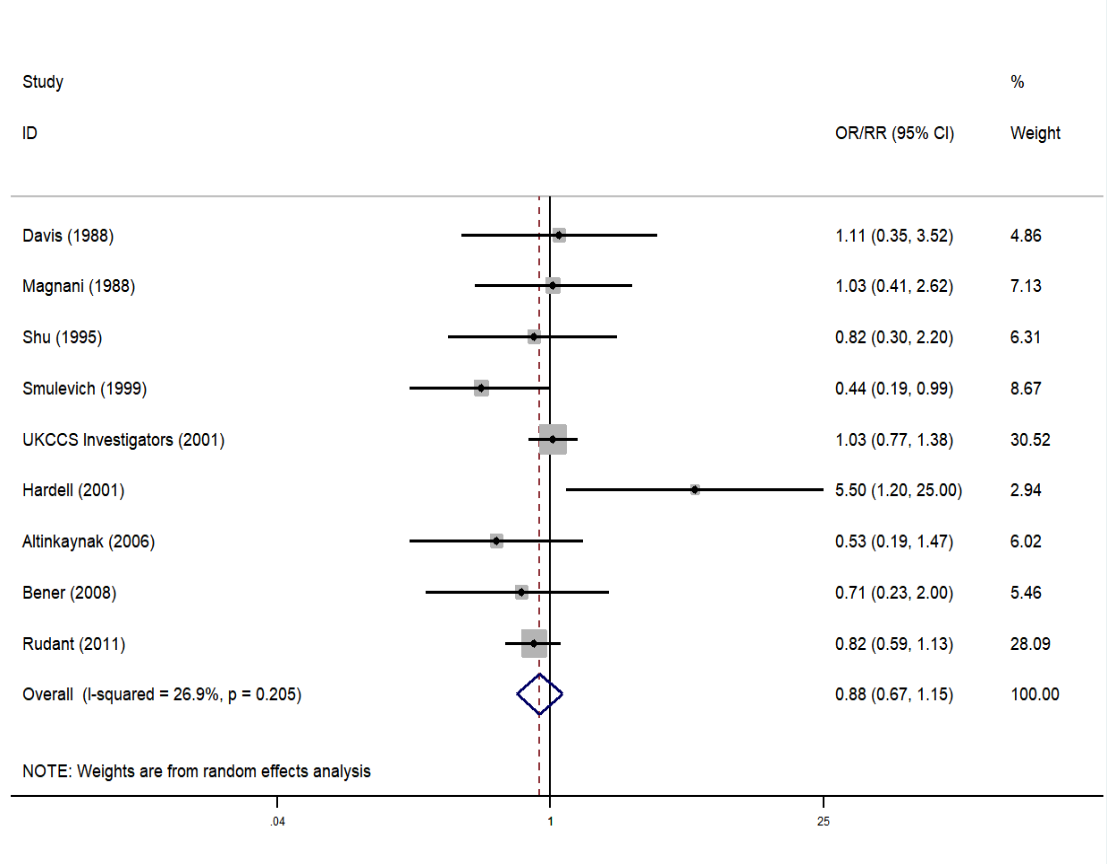


**(B.3)**


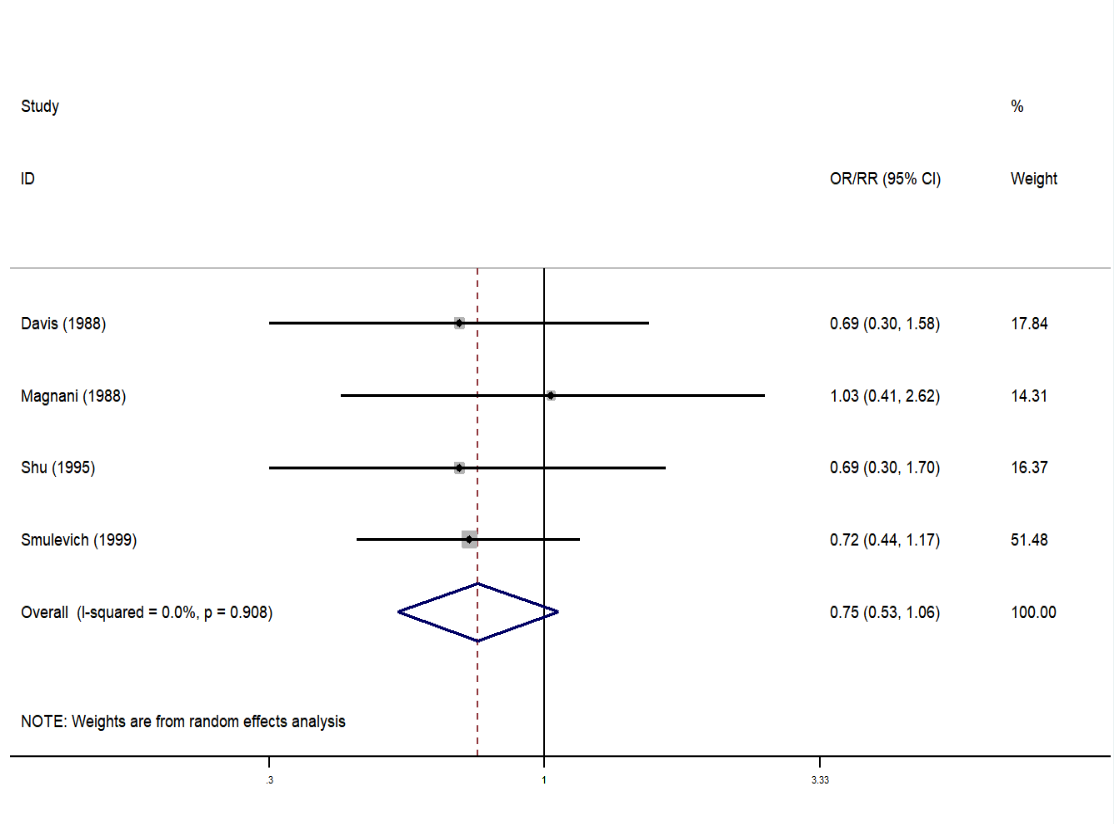


**(B.4)**


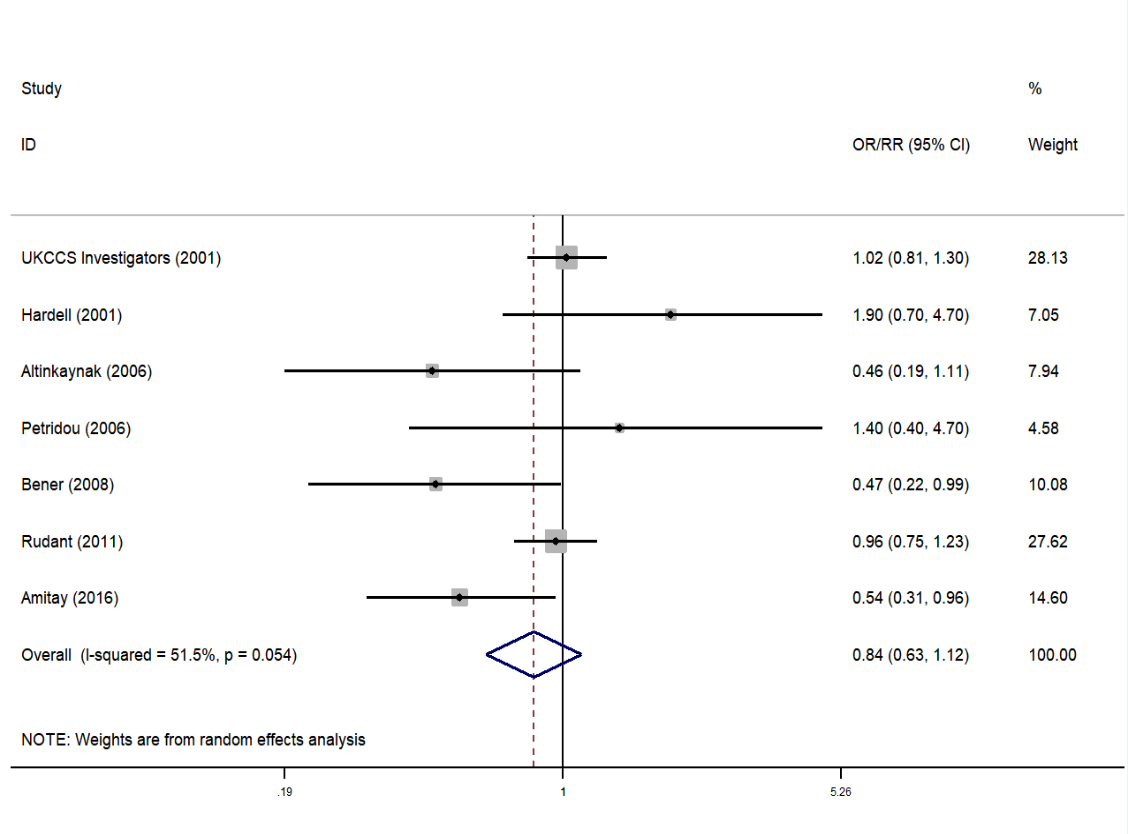


**(B.5)**


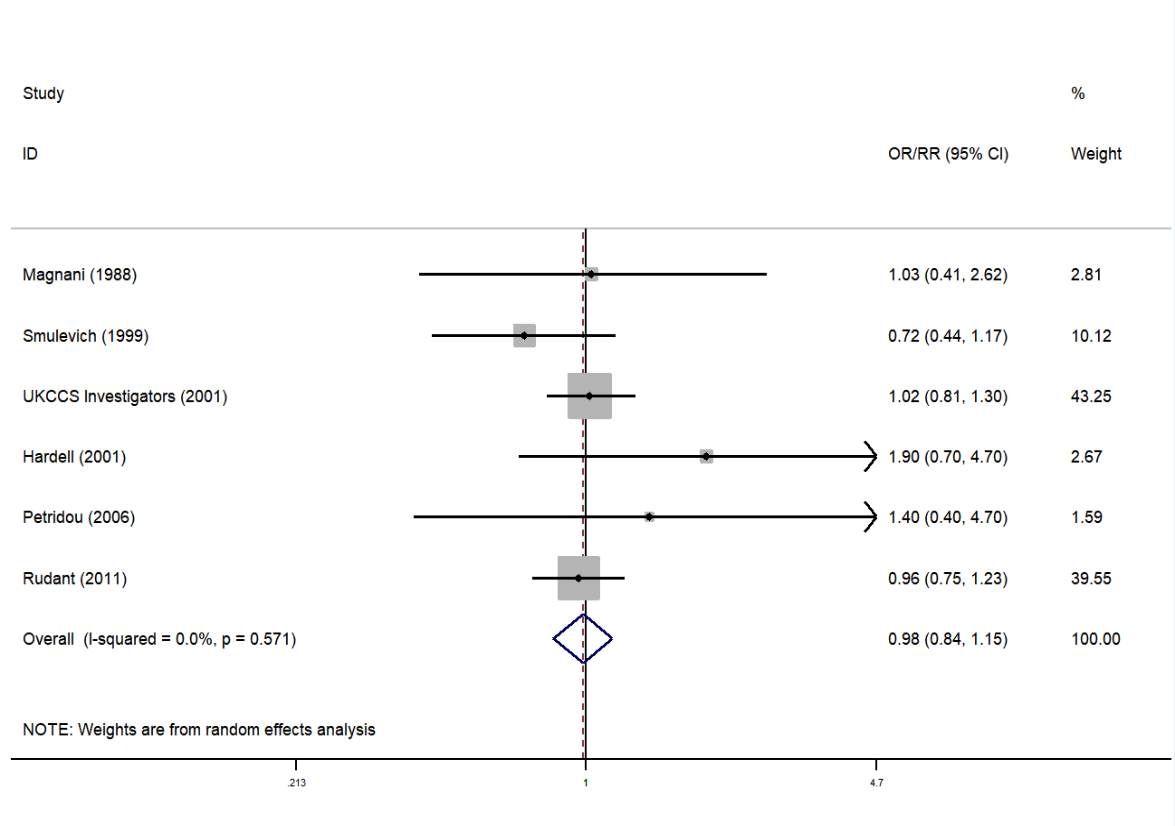


**(B.6)**


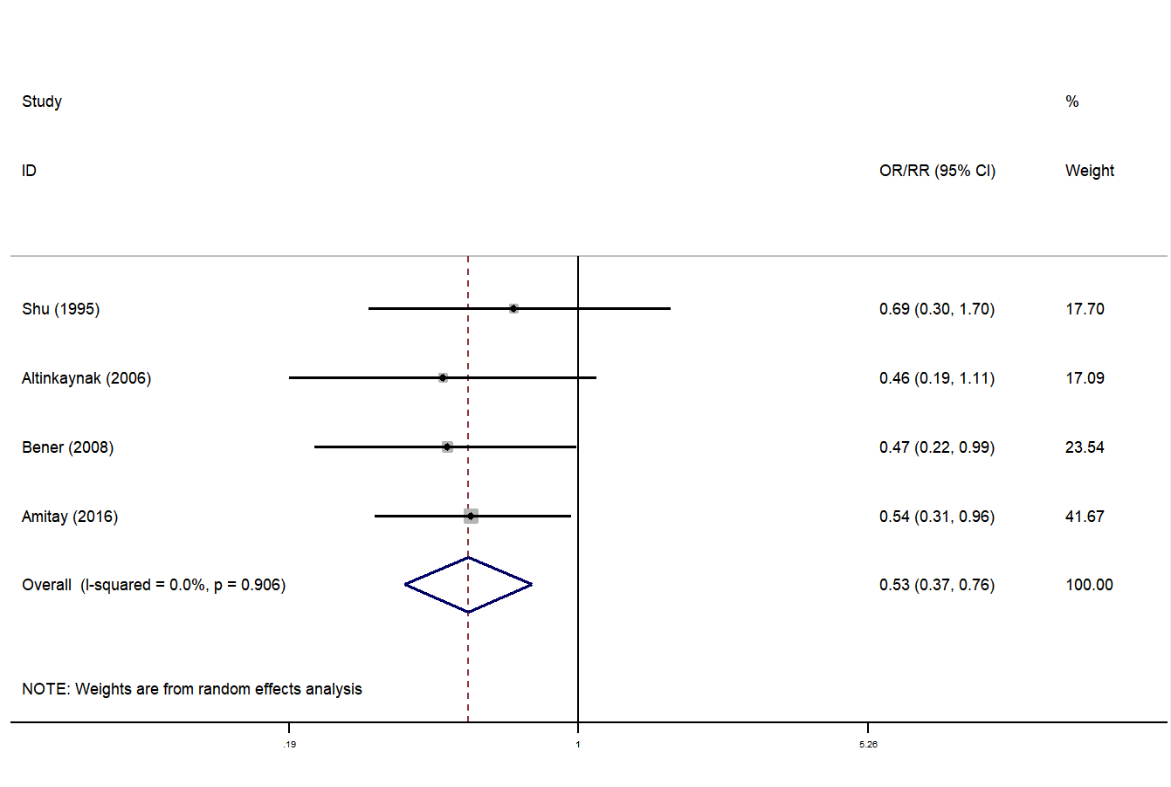


**(B.7)**


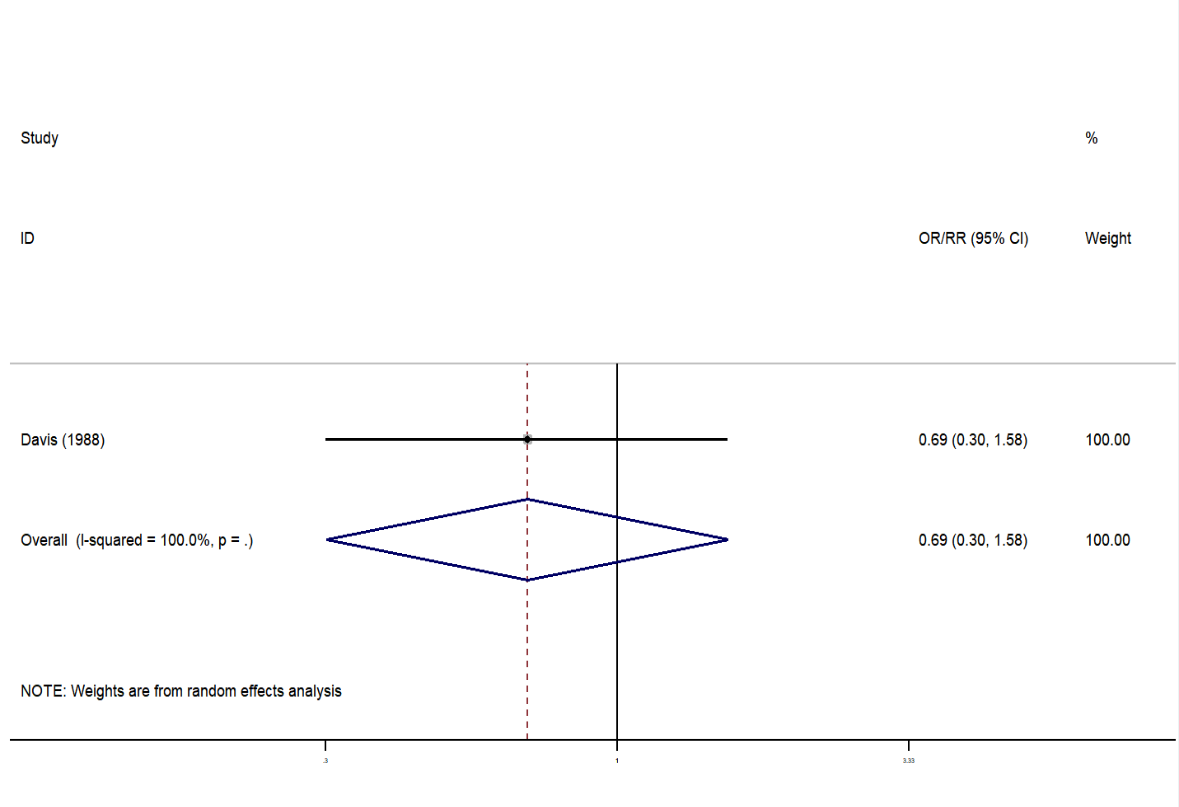


**(B.8)**


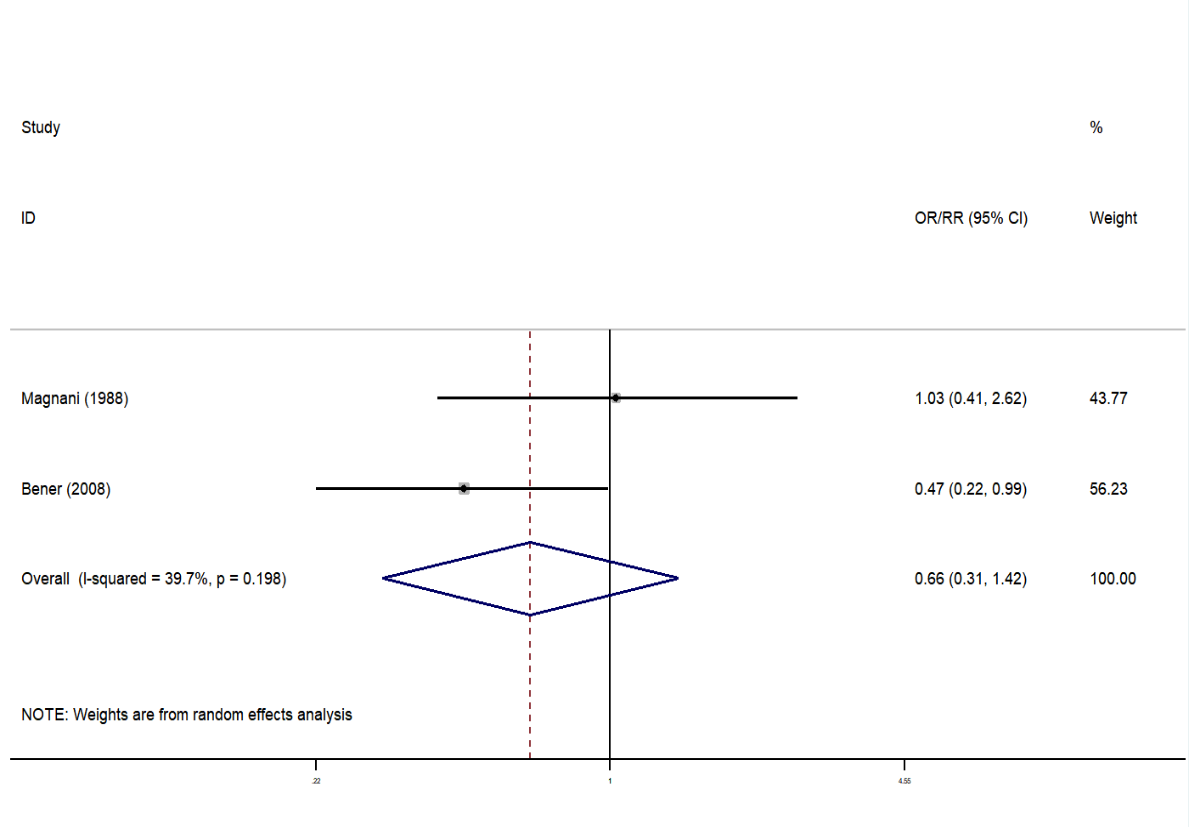


**(B.9)**


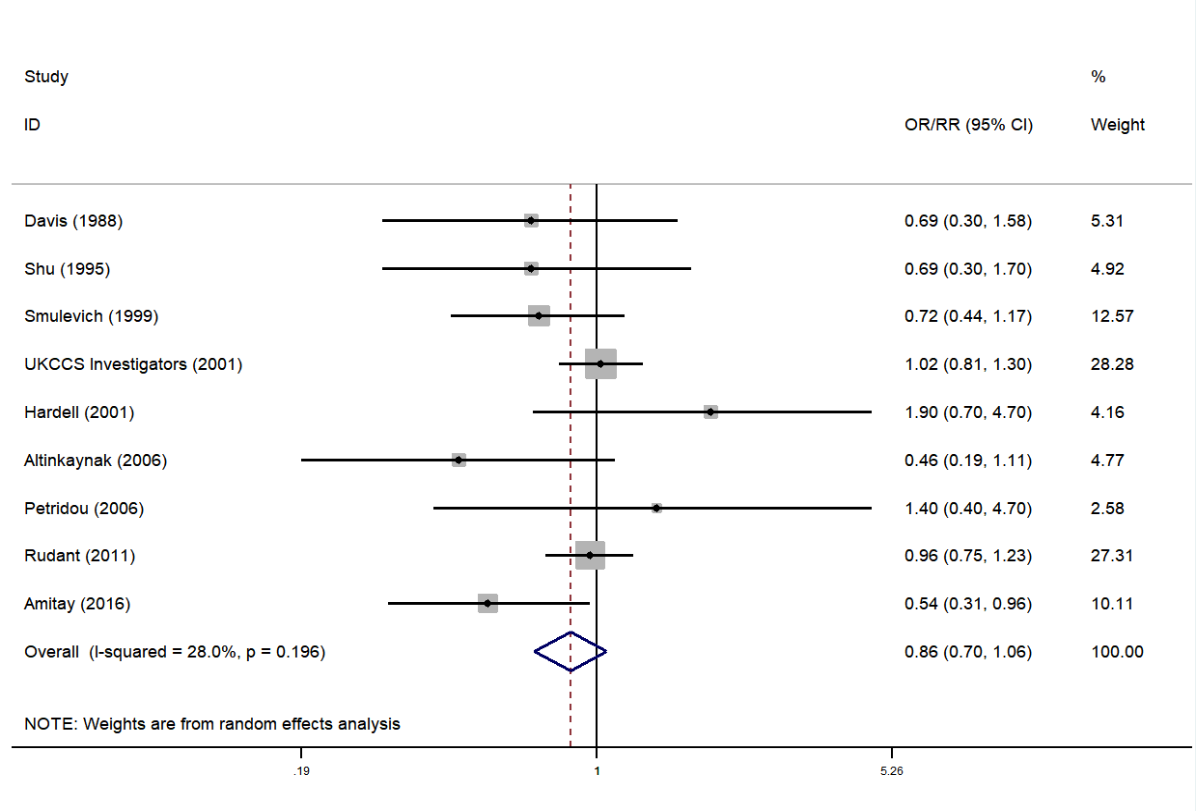


**(B.10)**


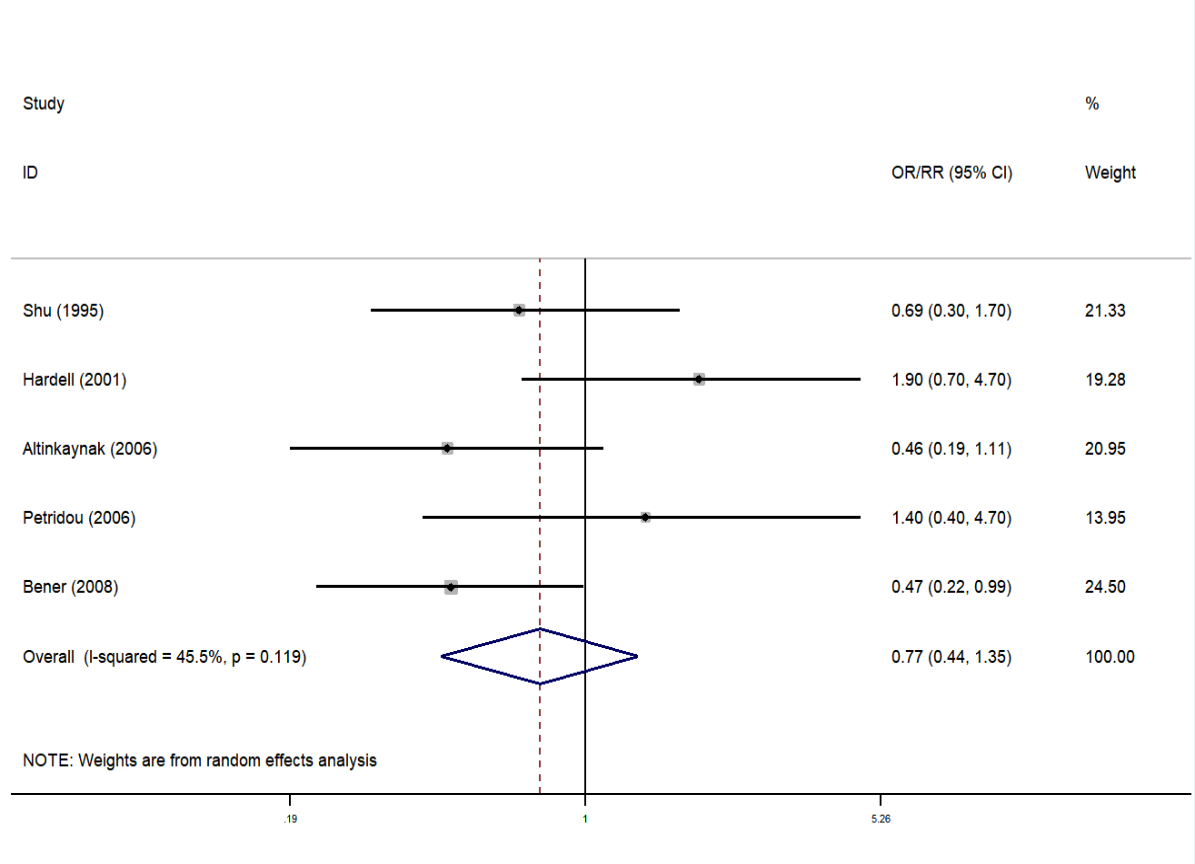


**(B.11)**


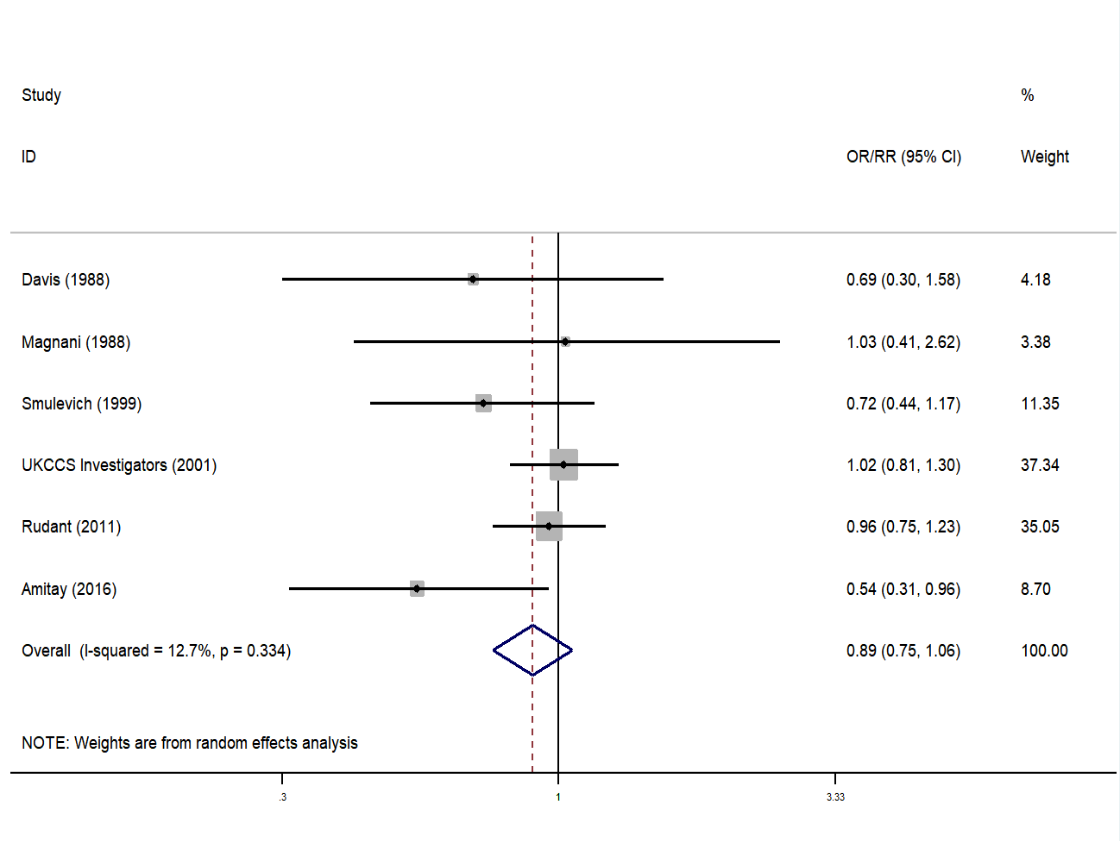


**(B.12)**


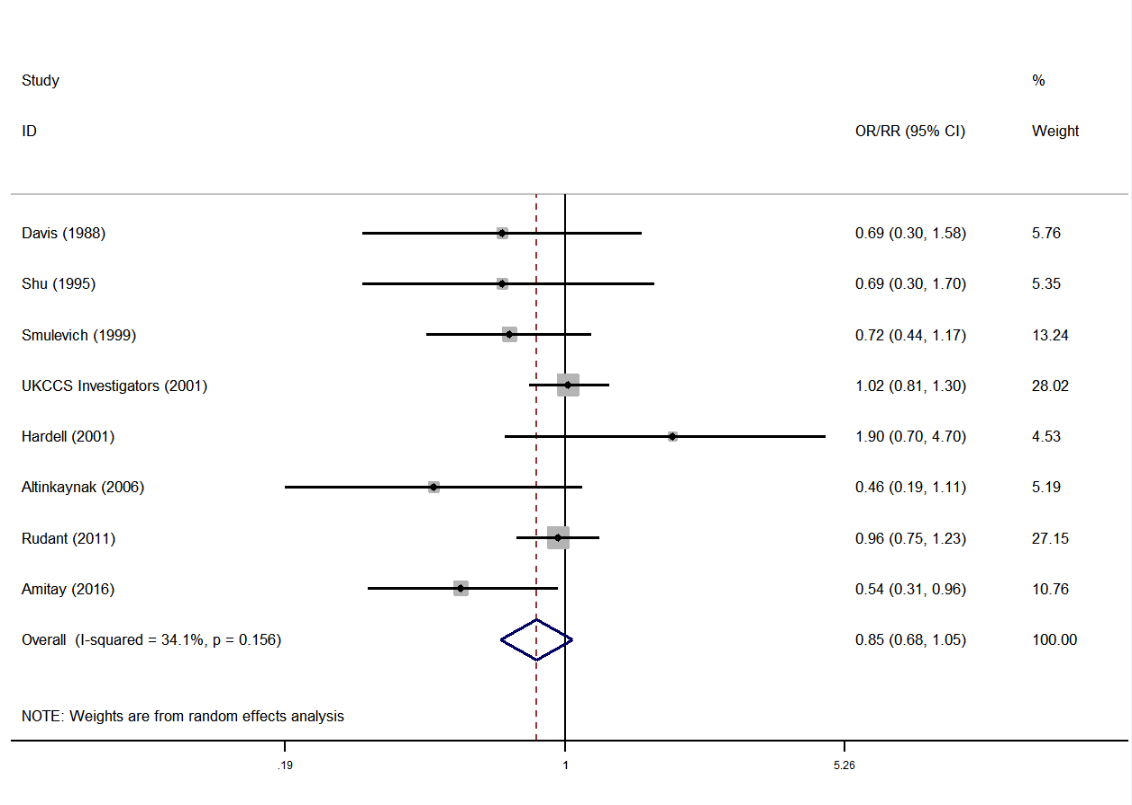


**(B.13)**


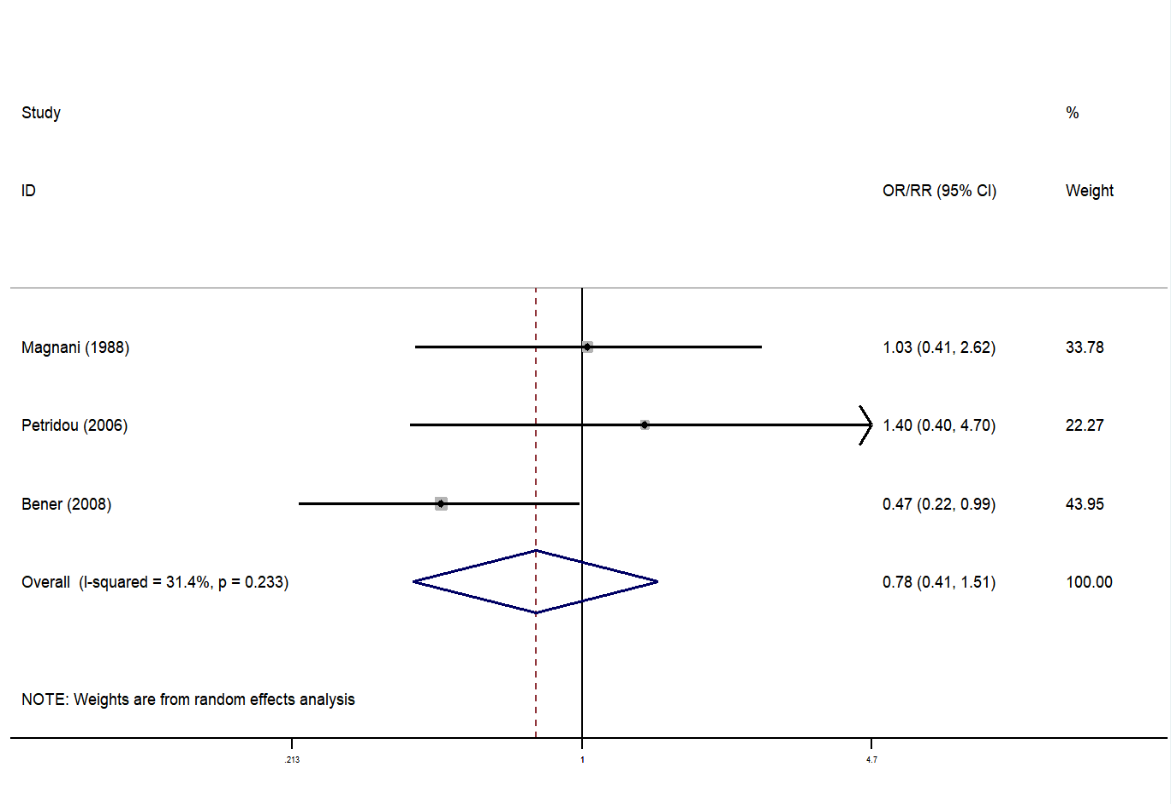


**(B.14)**


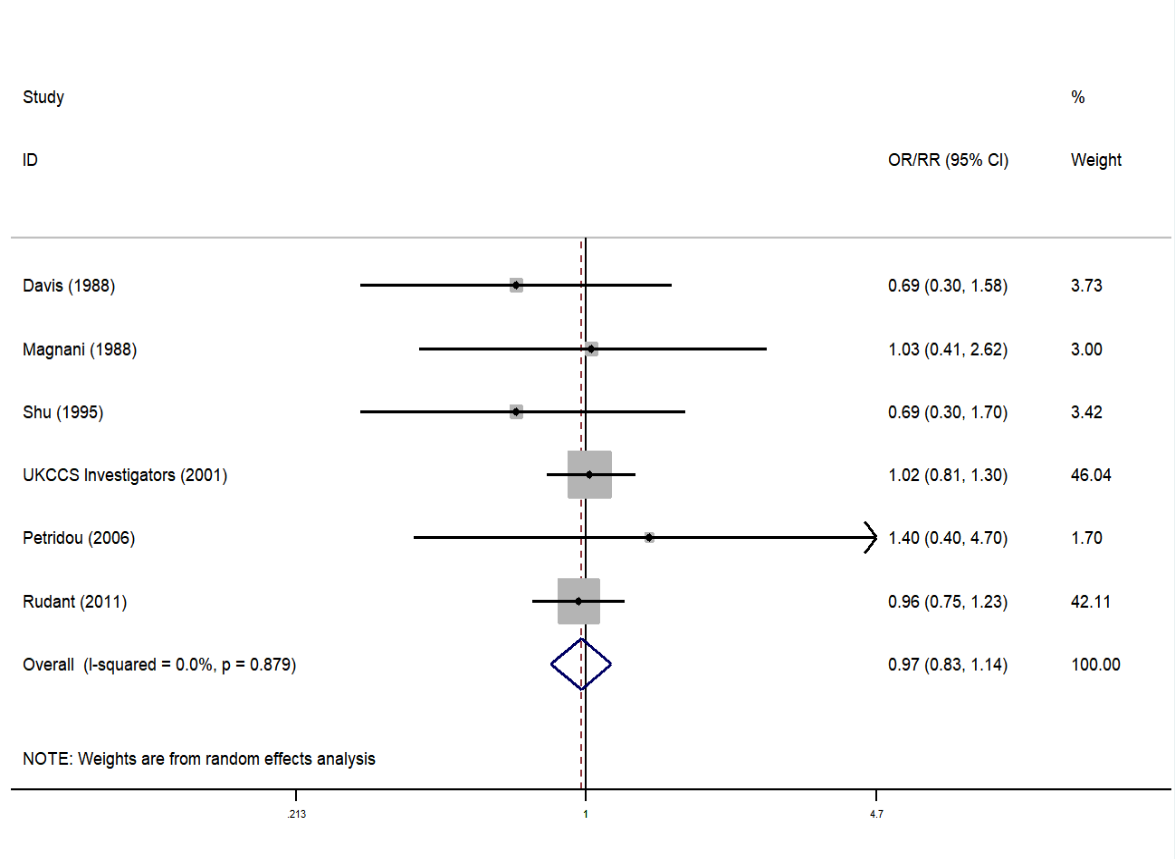


**(B.15)**


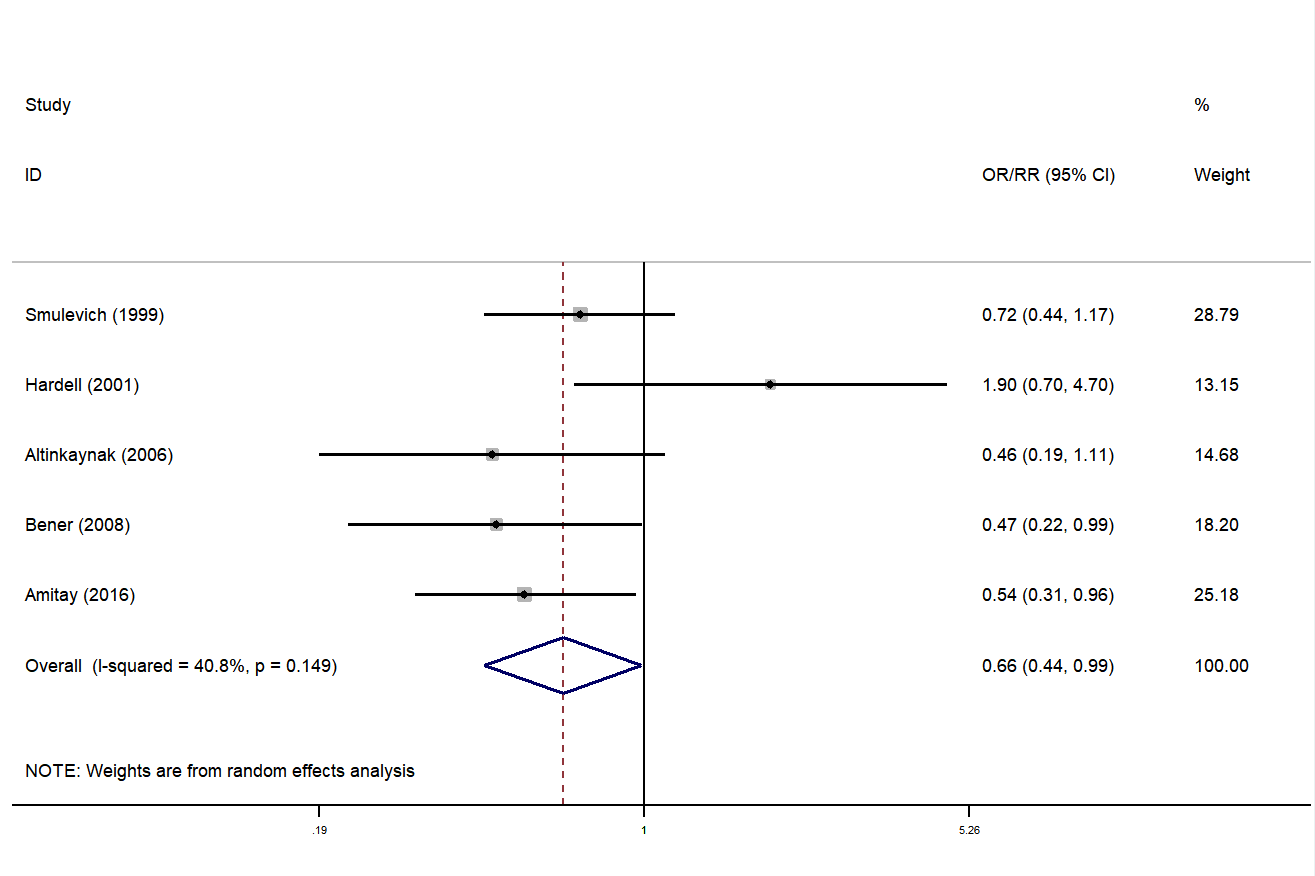


**(B.16)**


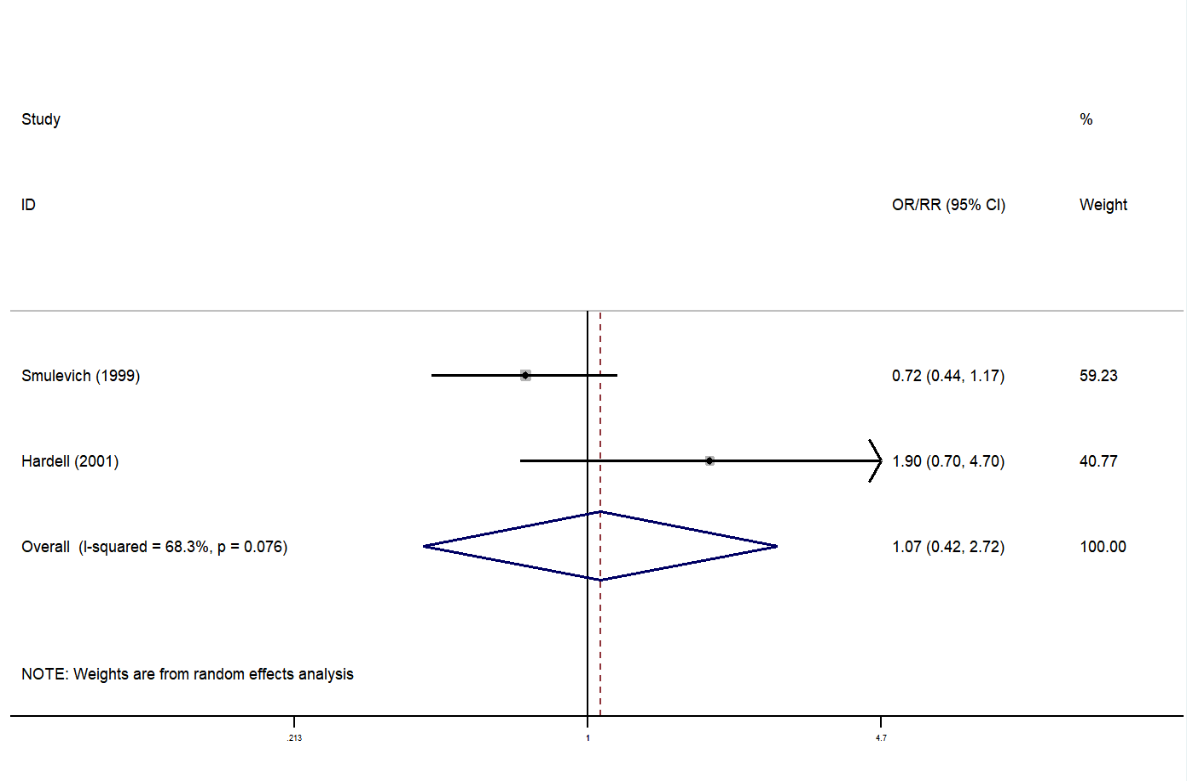


**(B.17)**


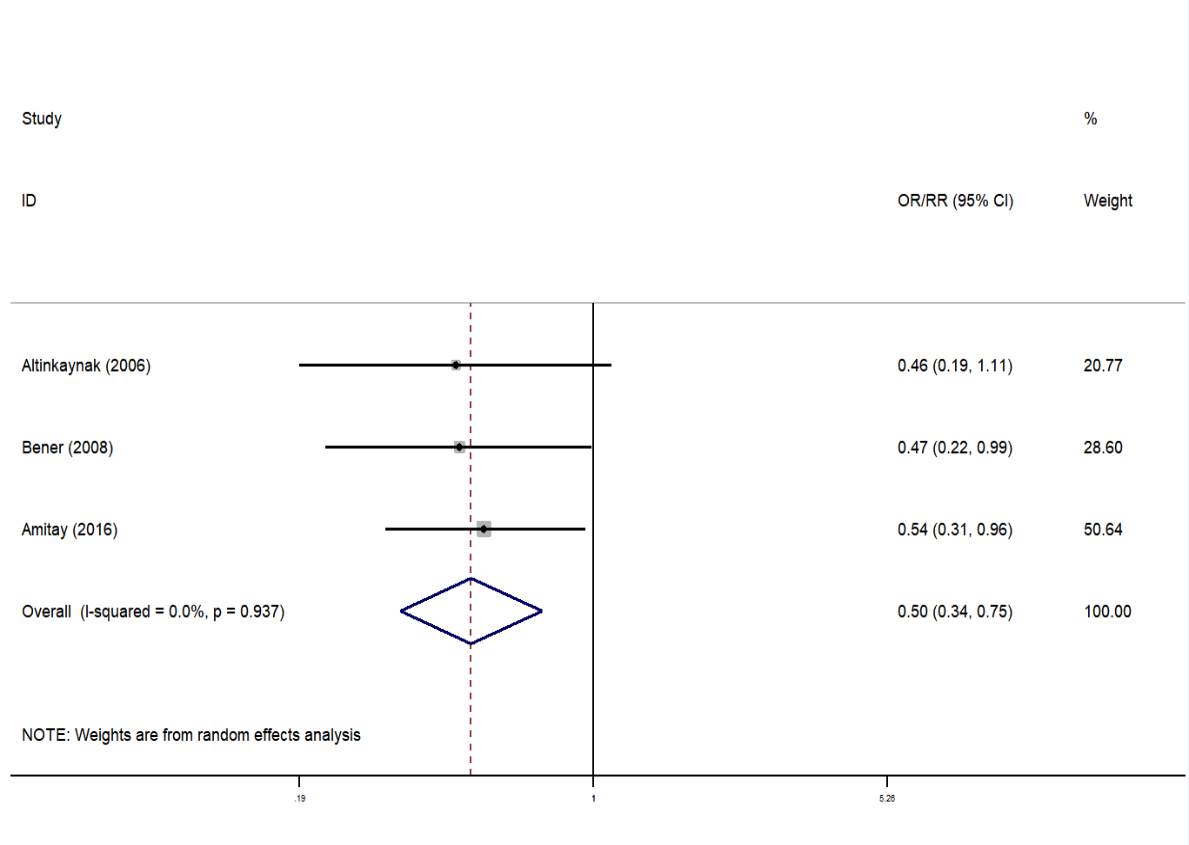


**Fig. S8** Forest plots of subgroup analysis of association between breastfeeding and childhood lymphoma risk in the order listed in Table 3.


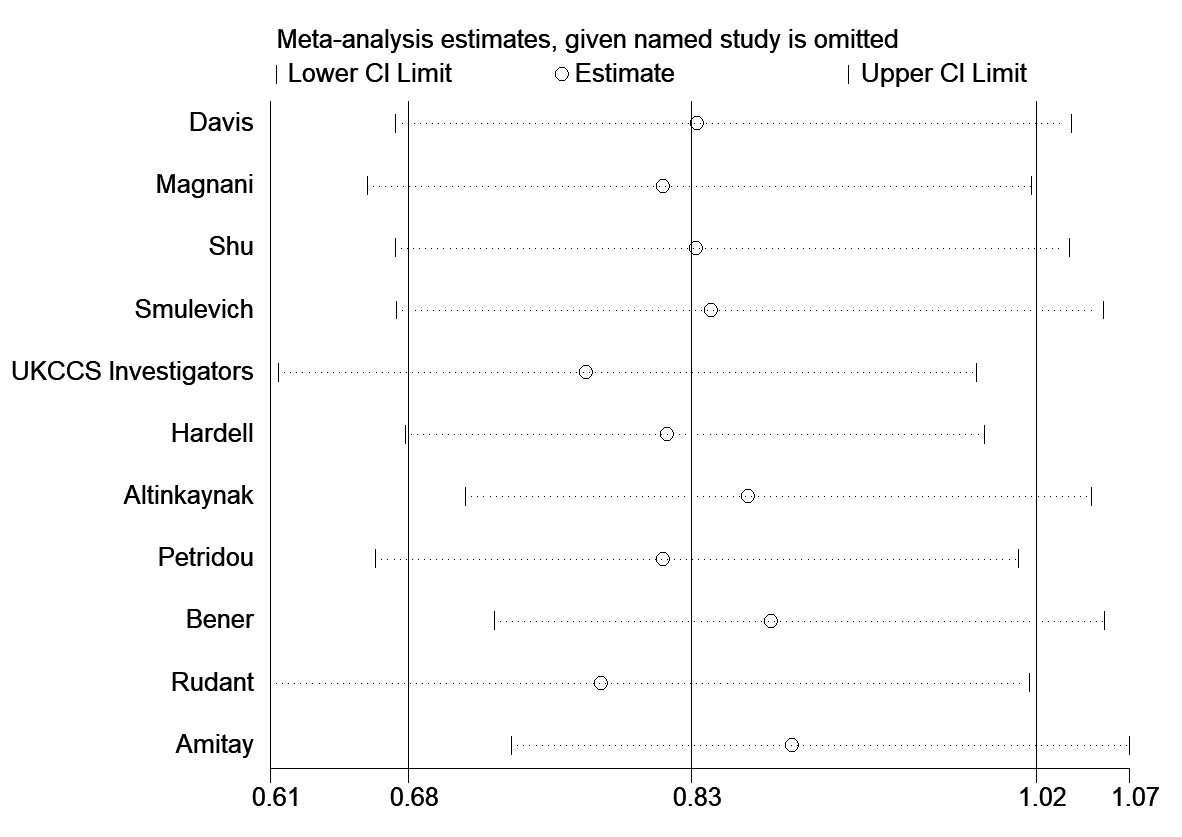


**Fig. S9** One-study-removed analysis on the association of breastfeeding with risk of childhood lymphoma.


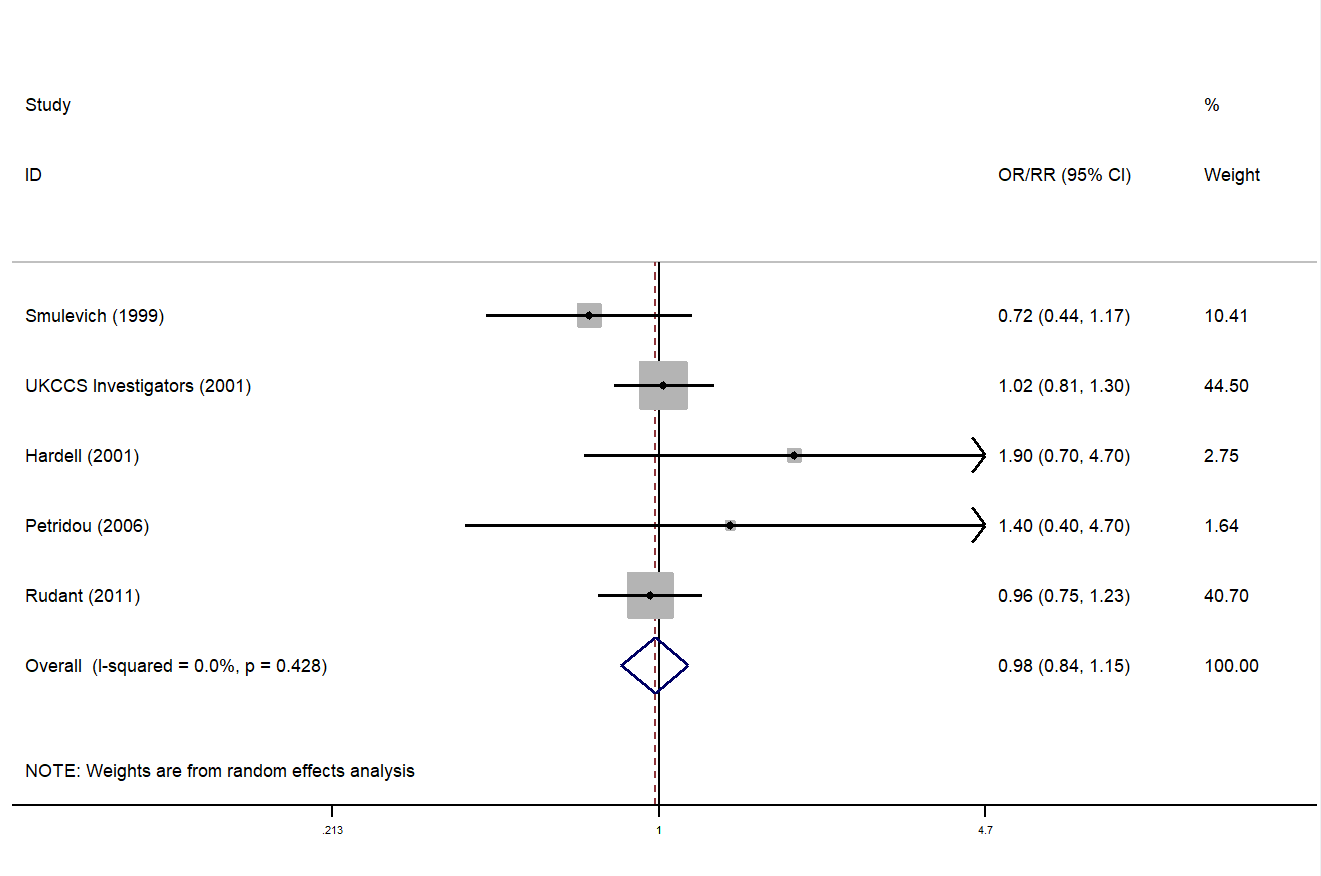


**Fig. S10** Pooled analysis of studies including only children aged 0-14 years old for the for the association of breastfeeding with risk of childhood lymphoma.


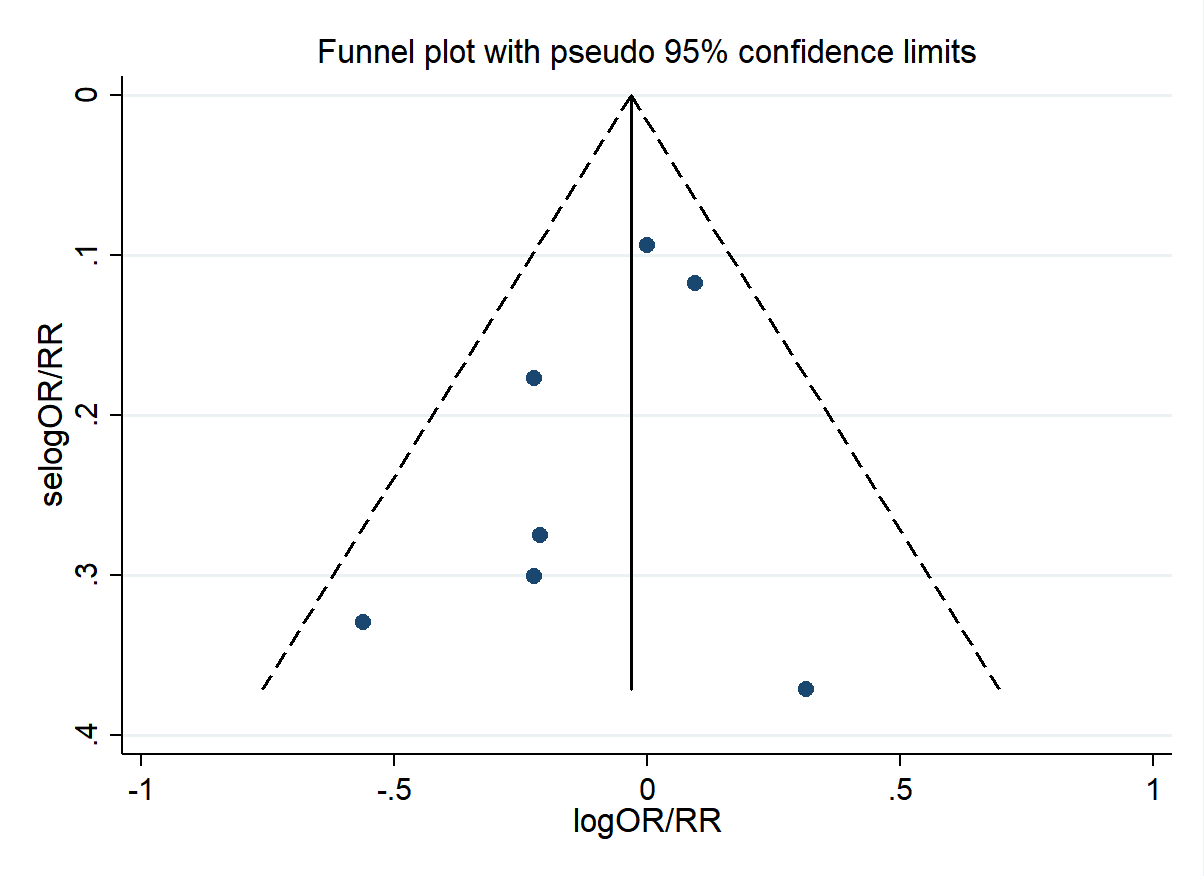


**Fig. S11** Begg’s funnel plots identifying the publication bias for the association between breastfeeding and risk of childhood brain tumors.

**(C.1)**

**
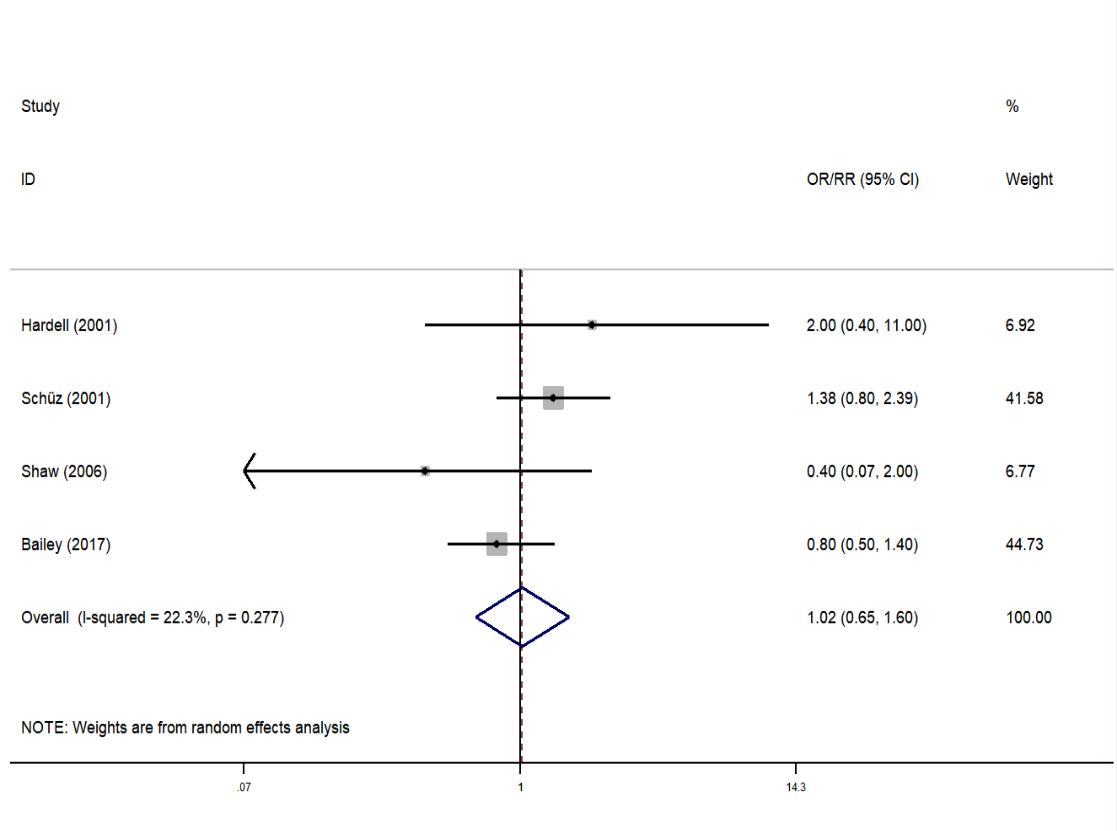
**

**(C.2)**

**
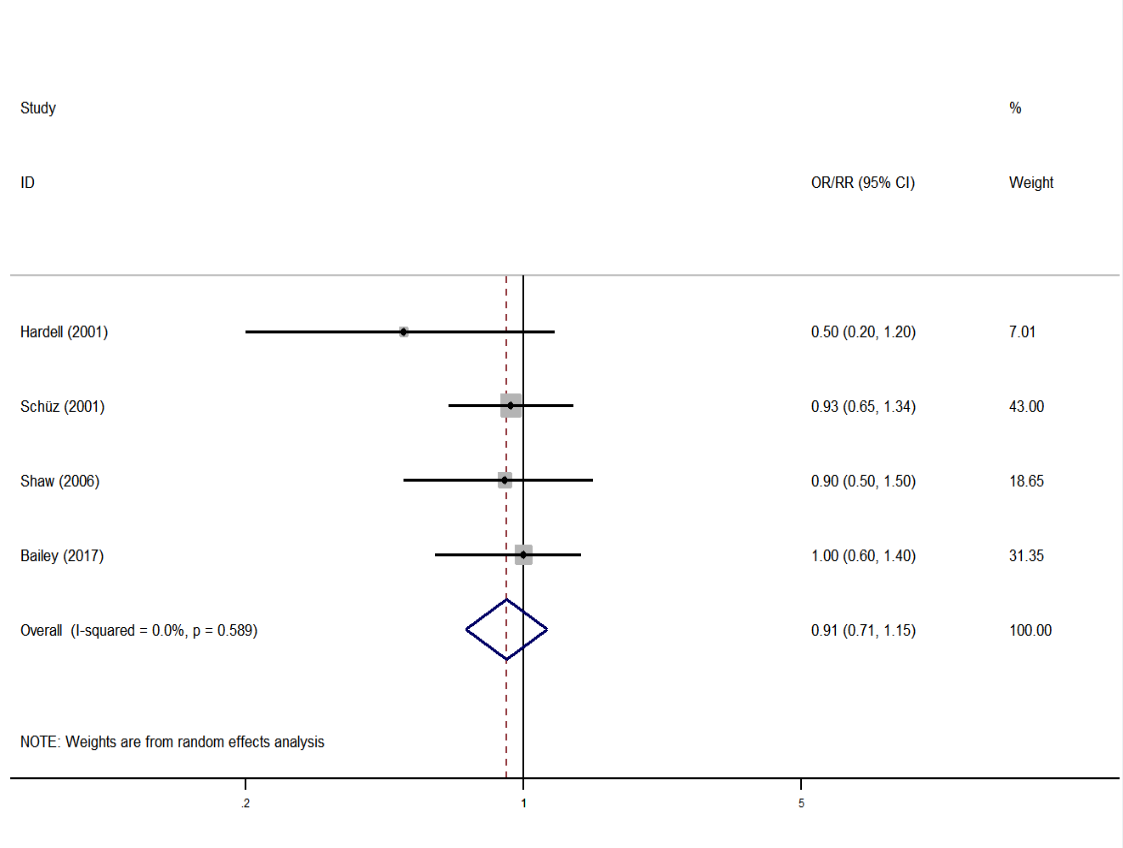
**

**(C.3)**

**
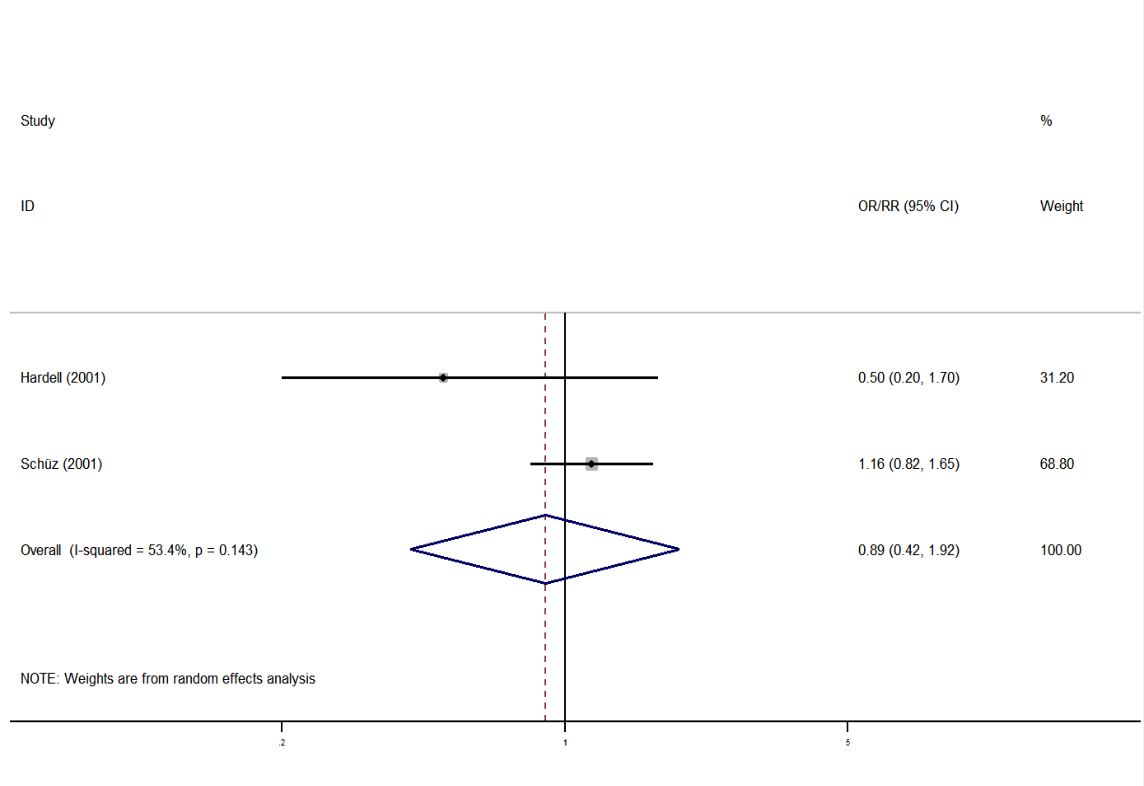
**

**(C.4)**

**
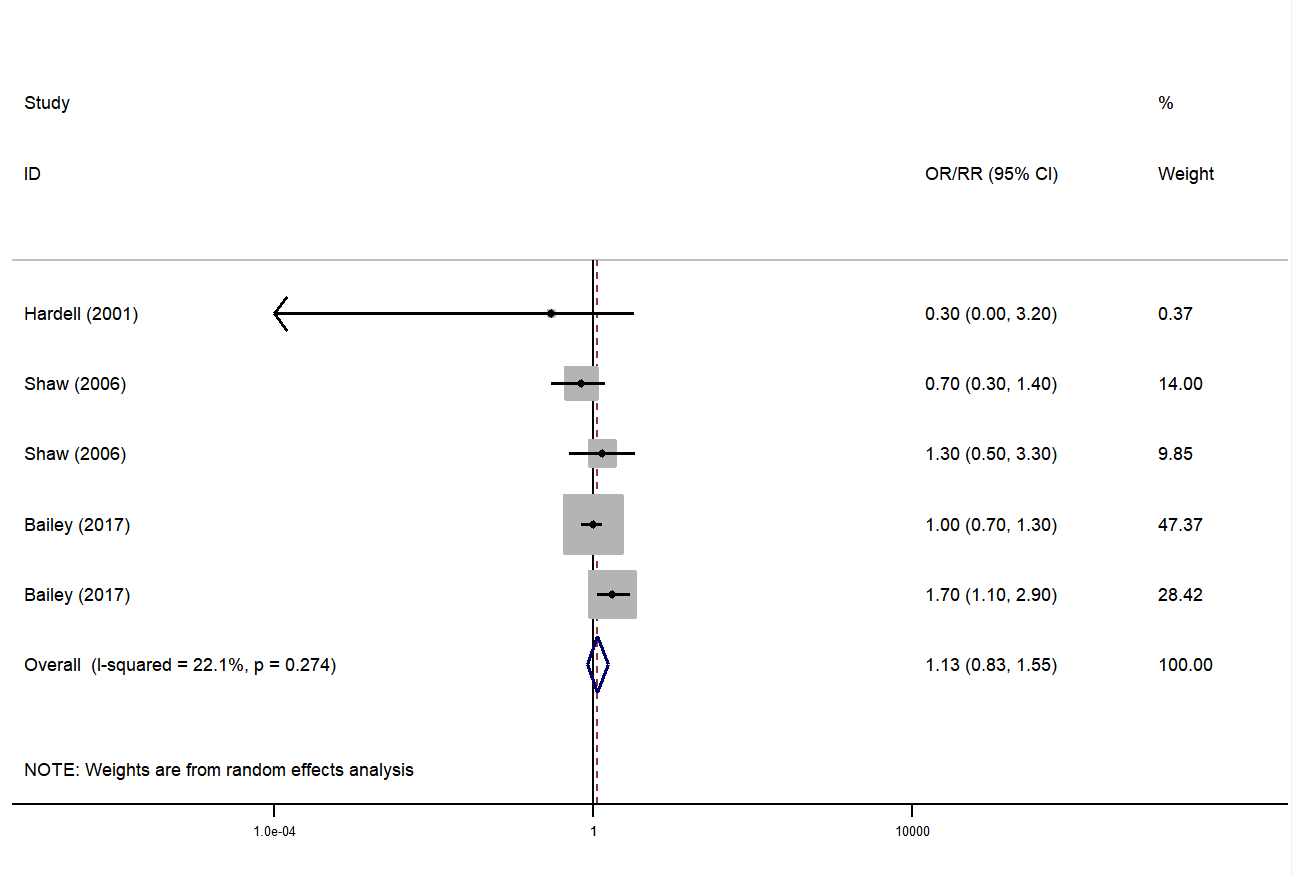
**

**(C.5)**

**
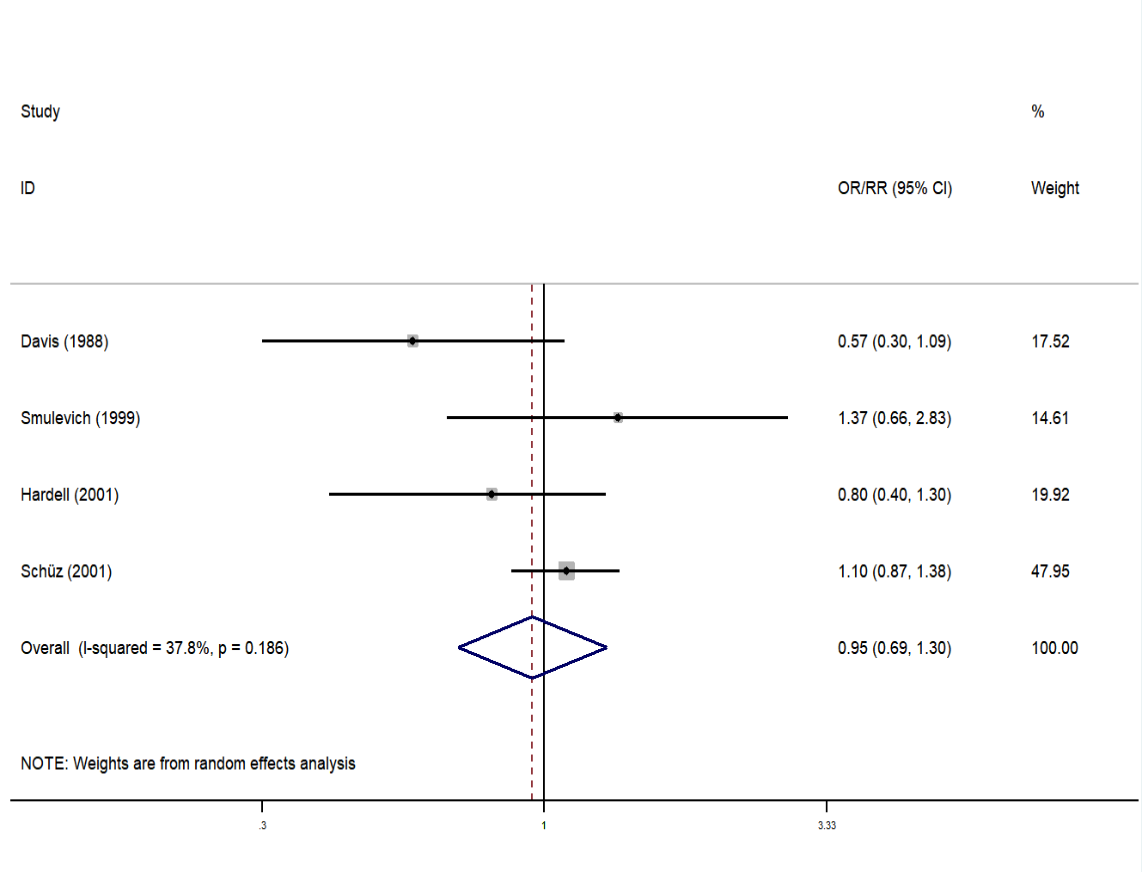
**

**(C.6)**

**
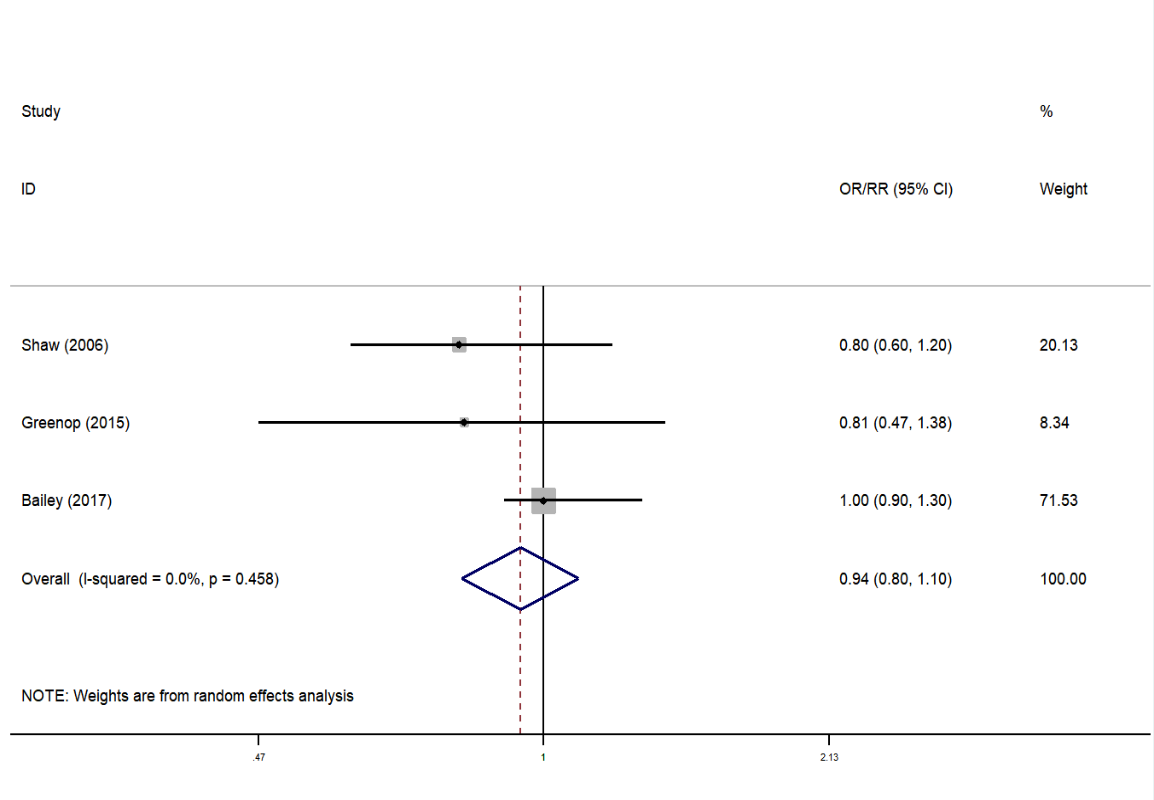
**

**(C.7)**

**
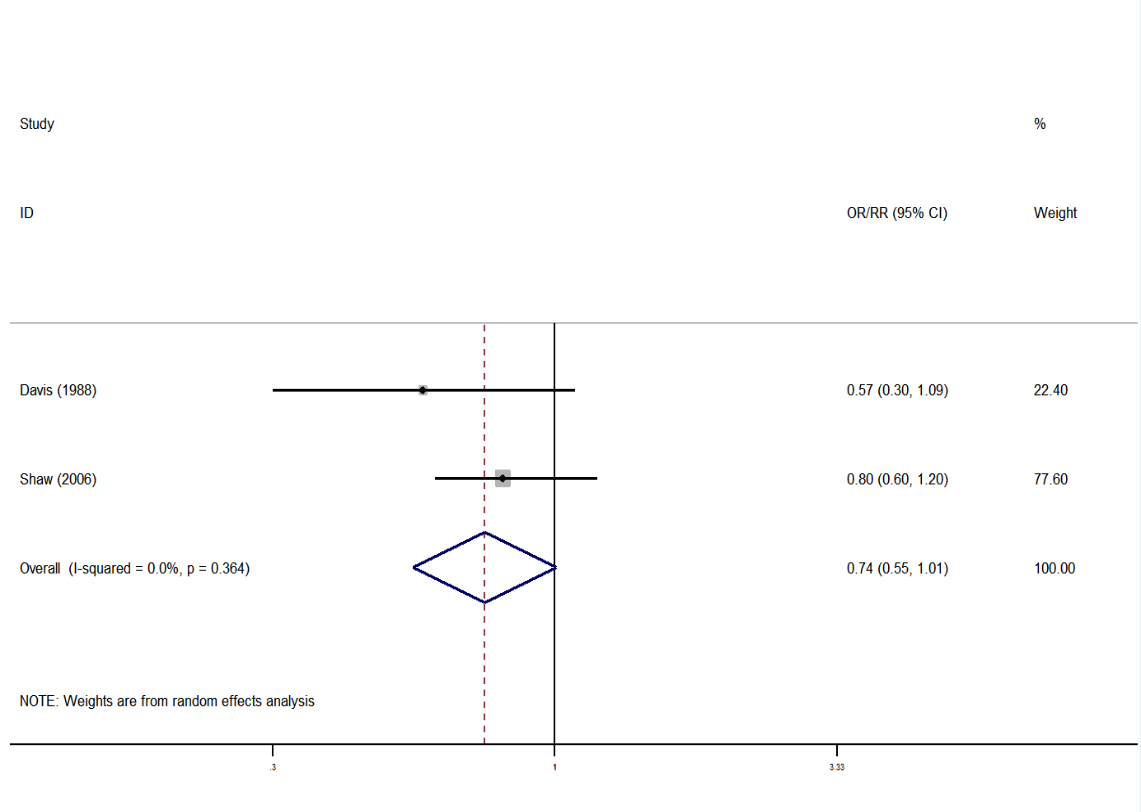
**

**(C.8)**

**
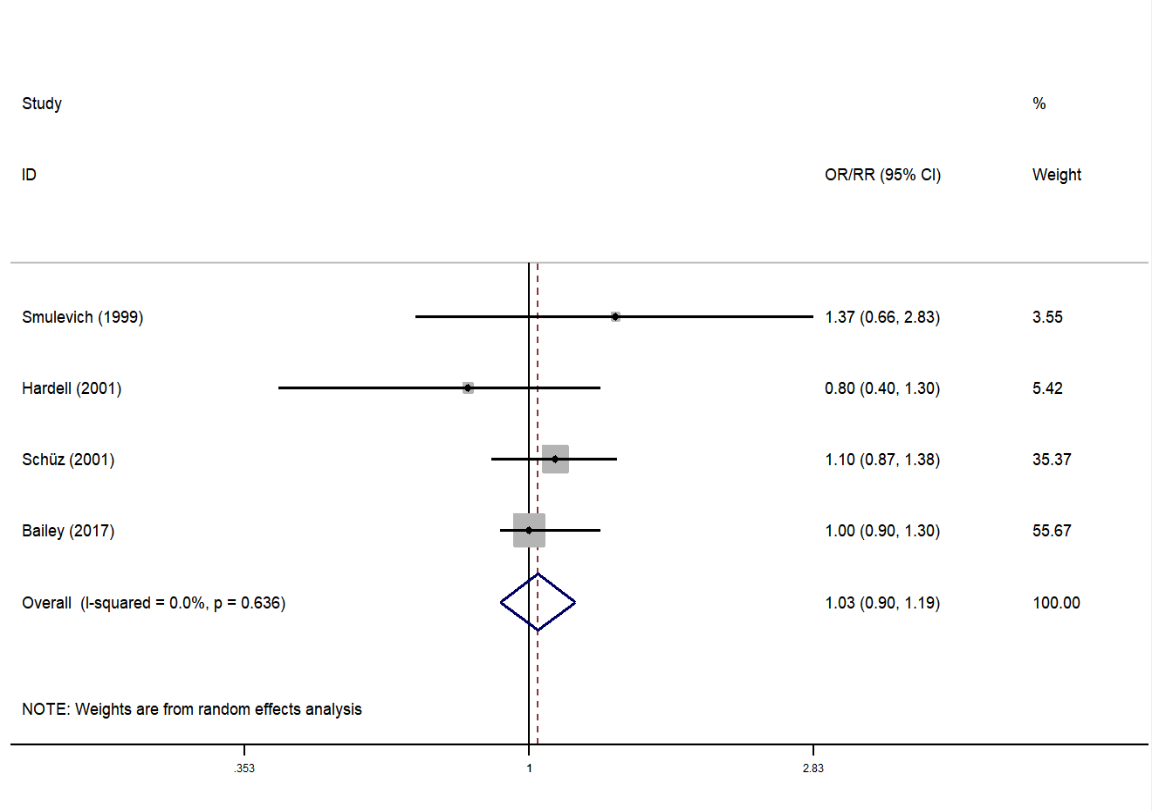
**

**(C.9)**

**
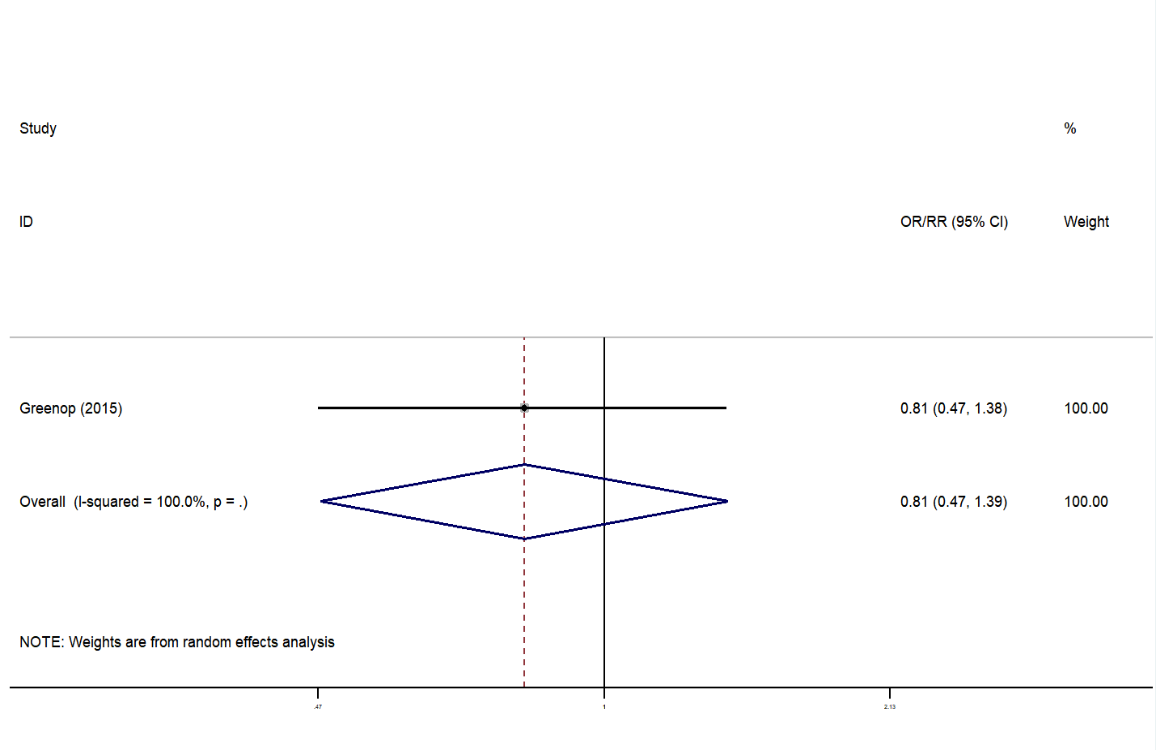
**

**(C.10)**

**
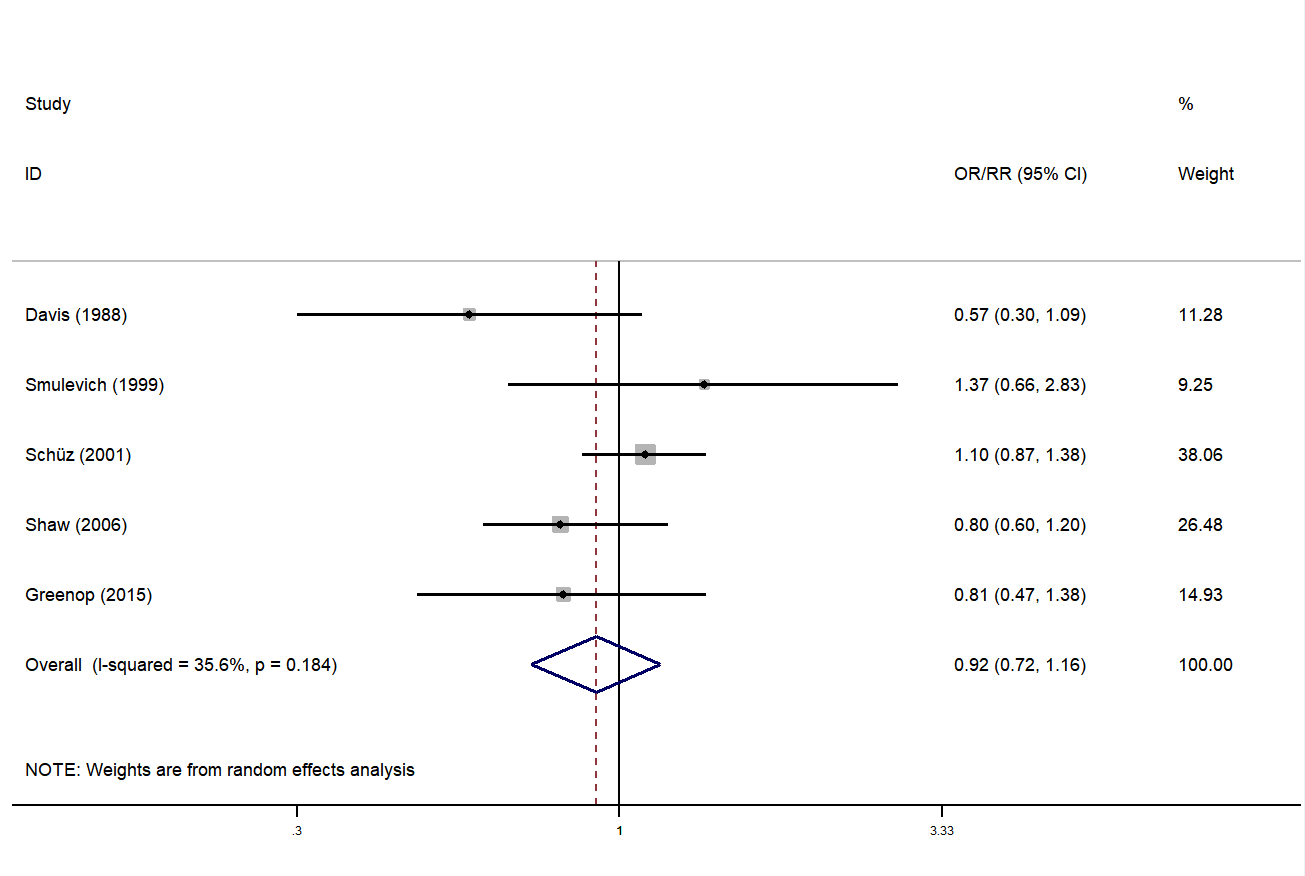
**

**(C.11)**

**
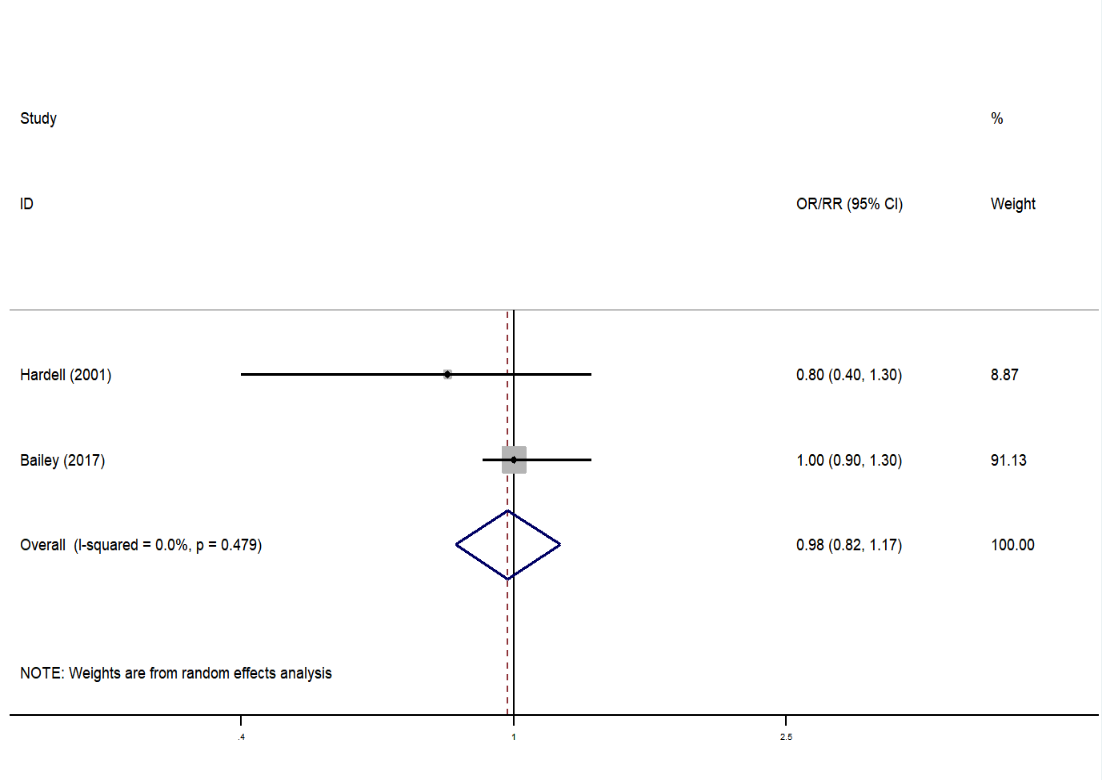
**

**(C.12)**

**
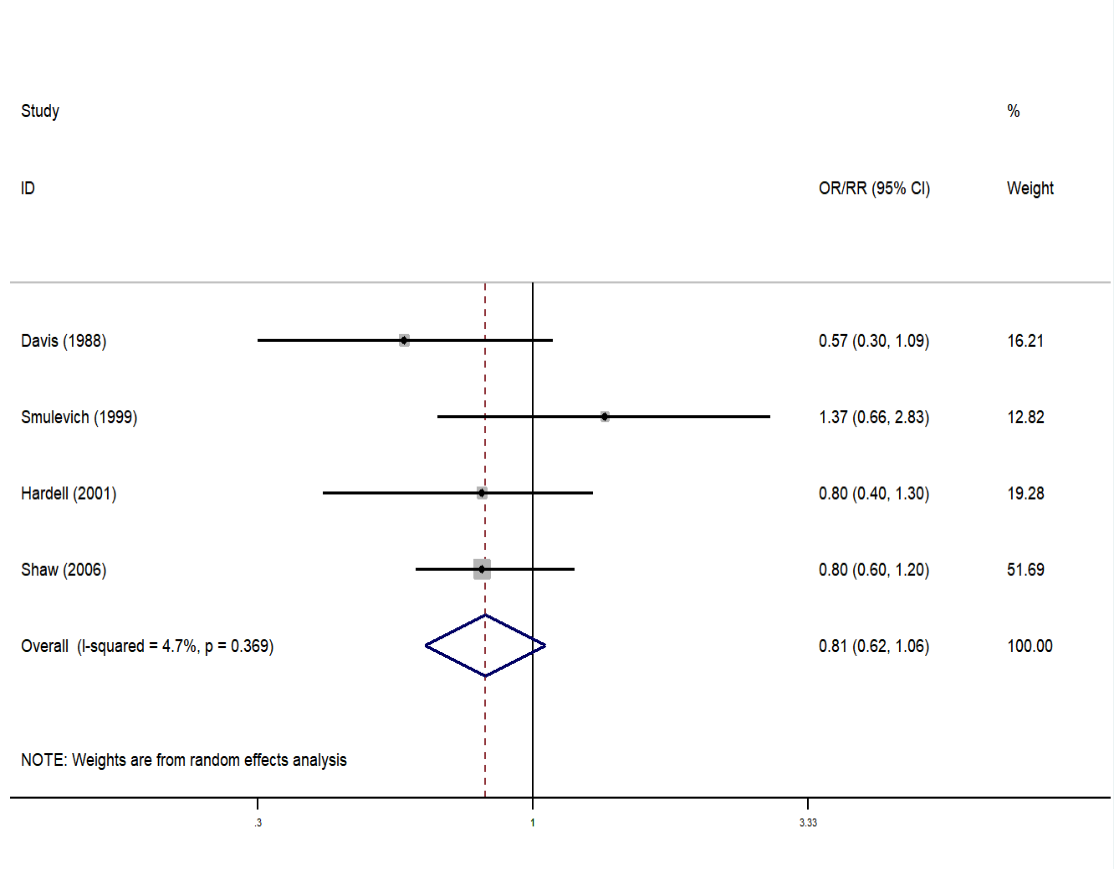
**

**(C.13)**

**
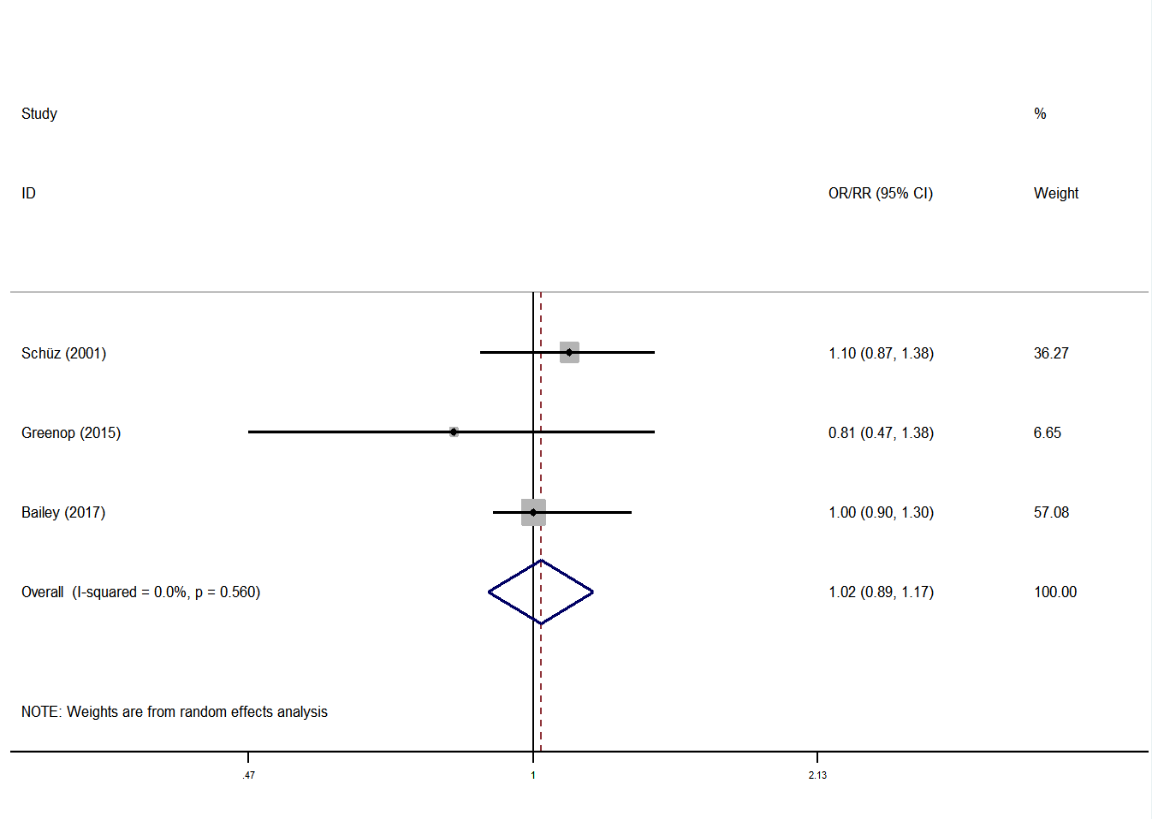
**

**(C.14)**

**
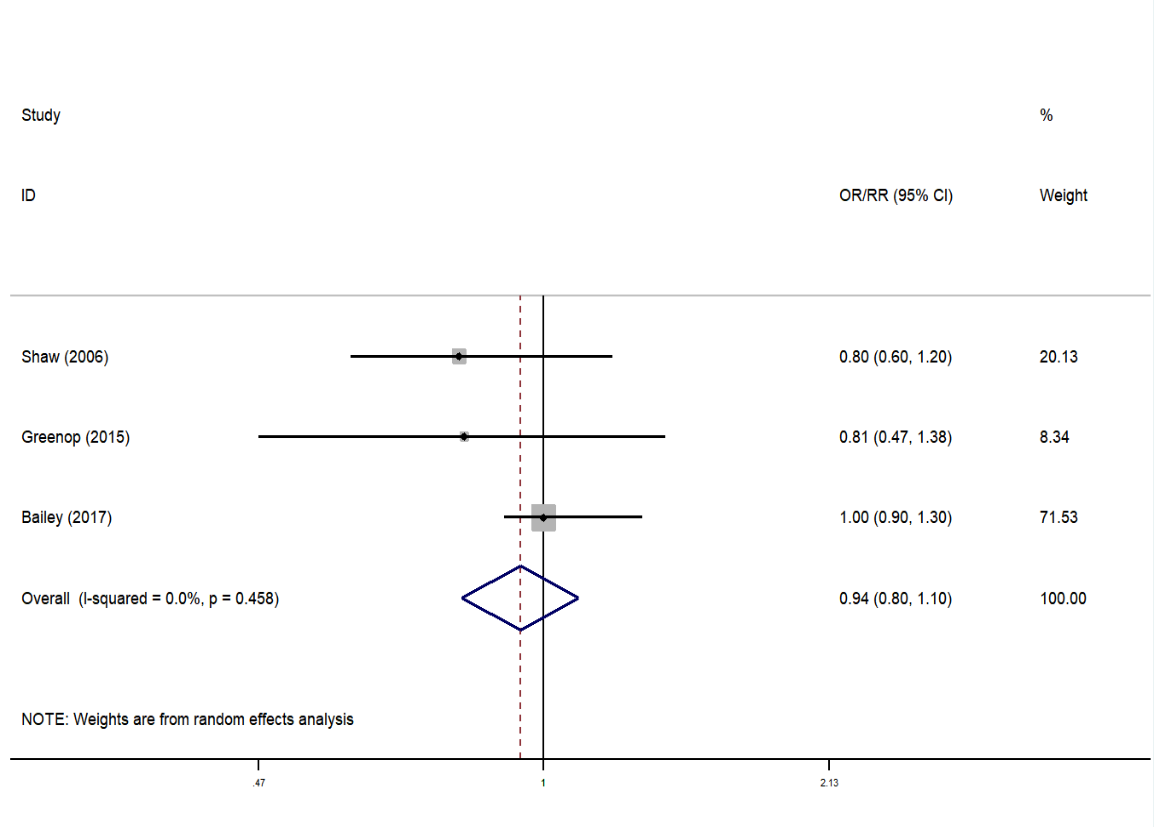
**

**(C.15)**

**
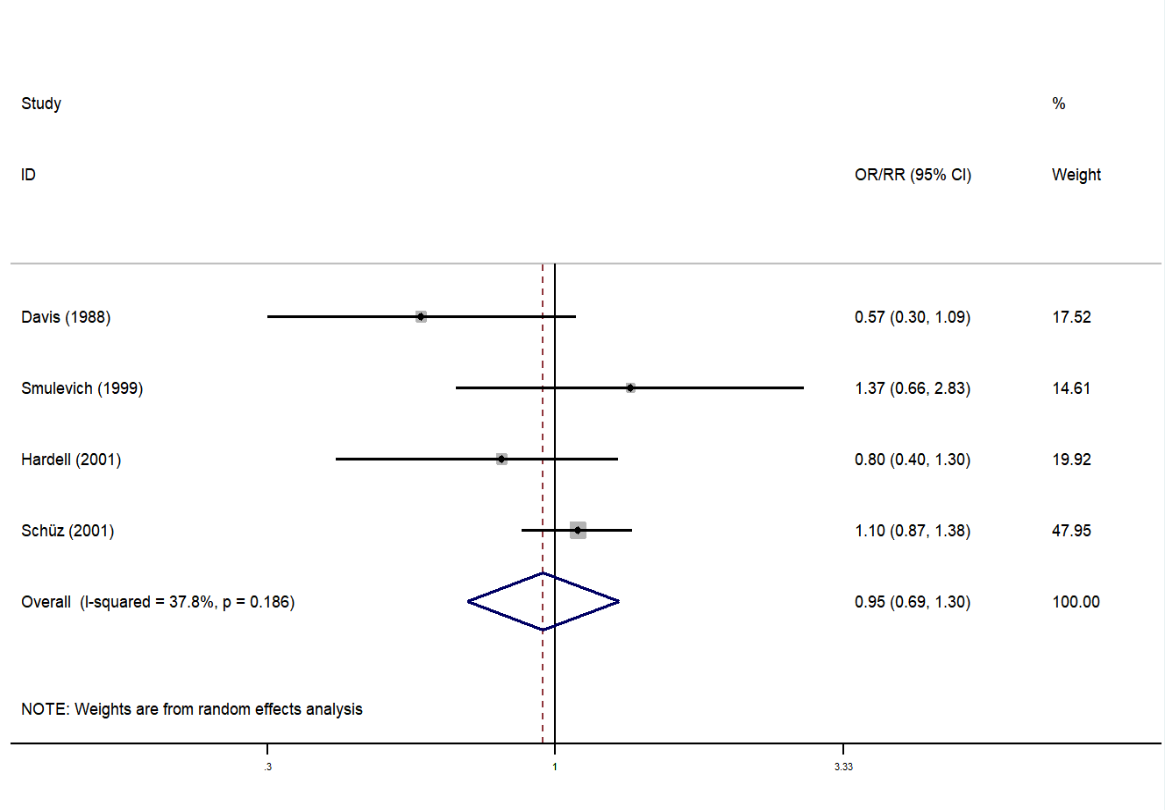
**

**(C.16)**

**
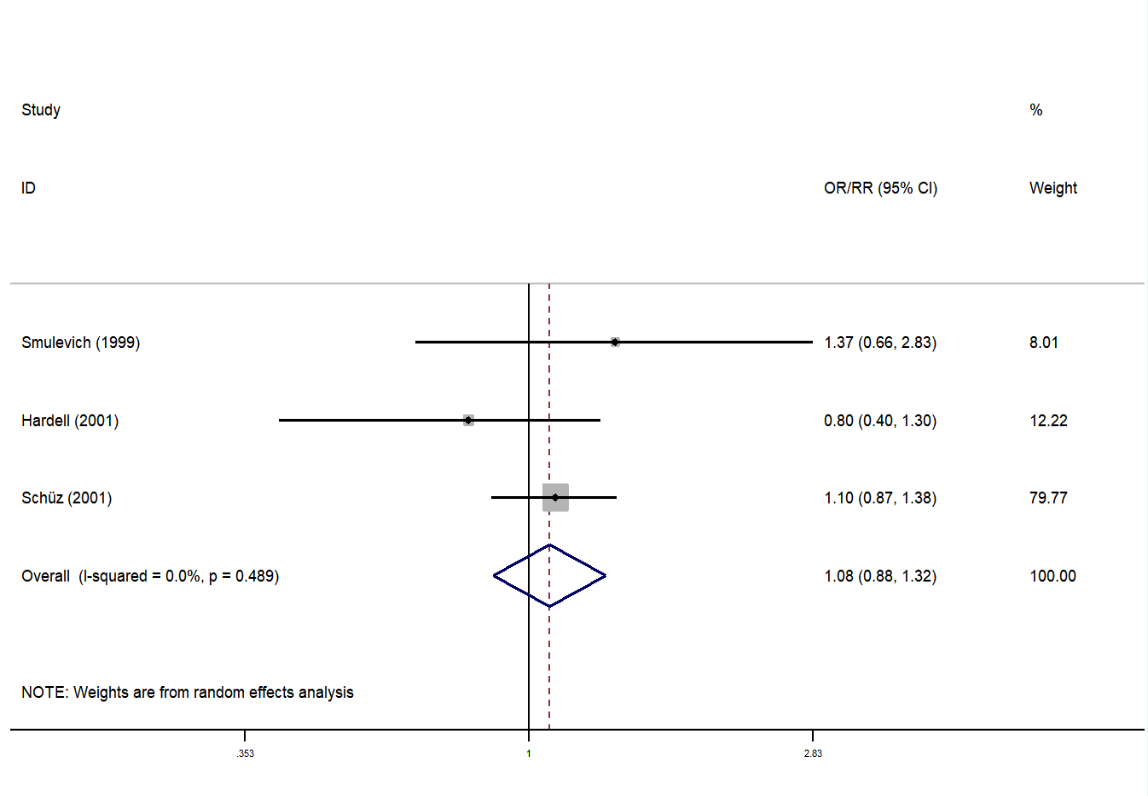
**

**(C.17)**

**
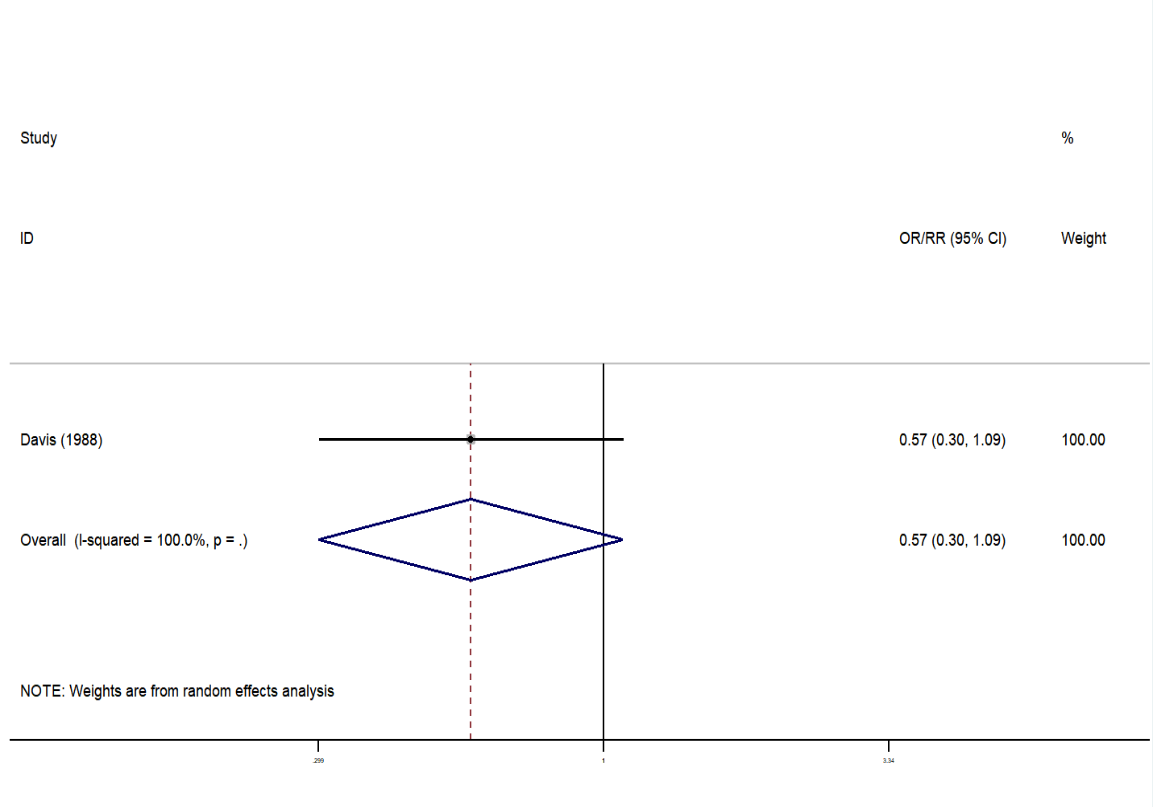
**

**Fig. S12** Forest plots of subgroup analysis of association between breastfeeding and risk of childhood brain tumors in the order listed in Table 4.


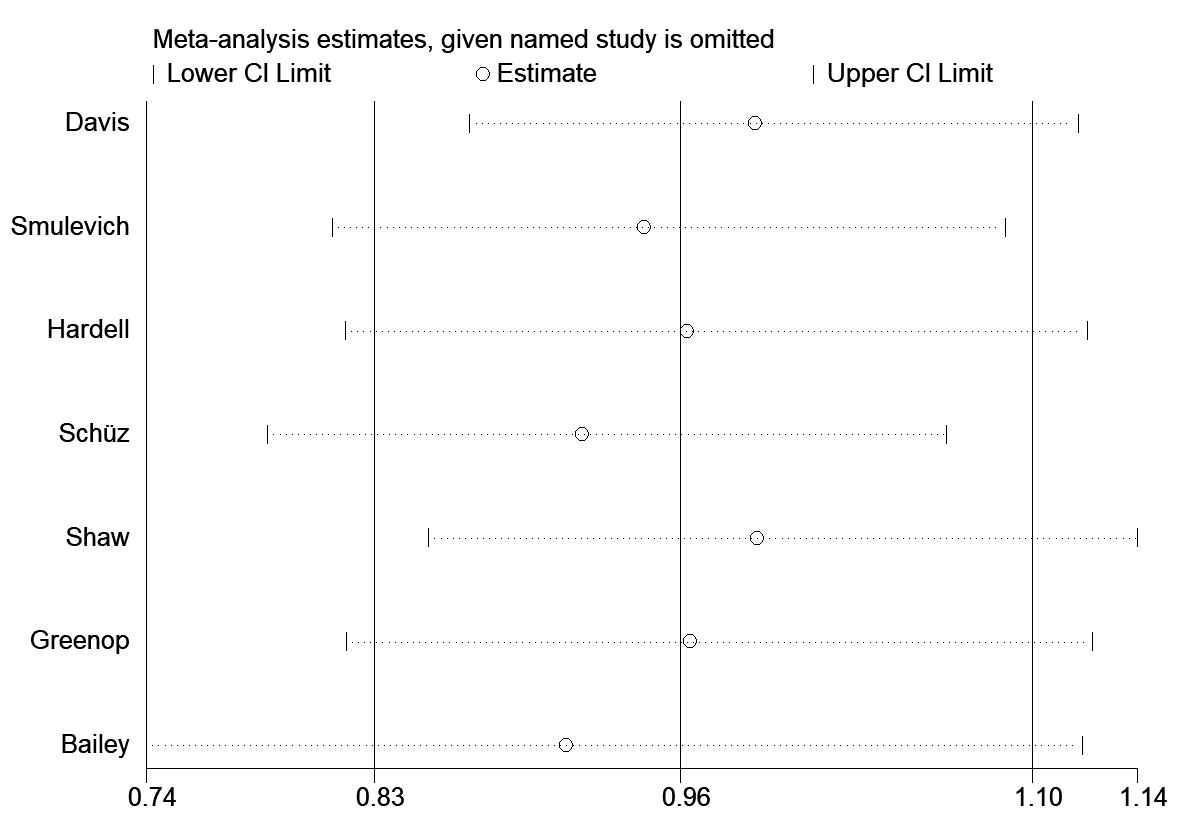


**Fig. S13** One-study-removed analysis on the association of breastfeeding with risk of childhood brain tumors.


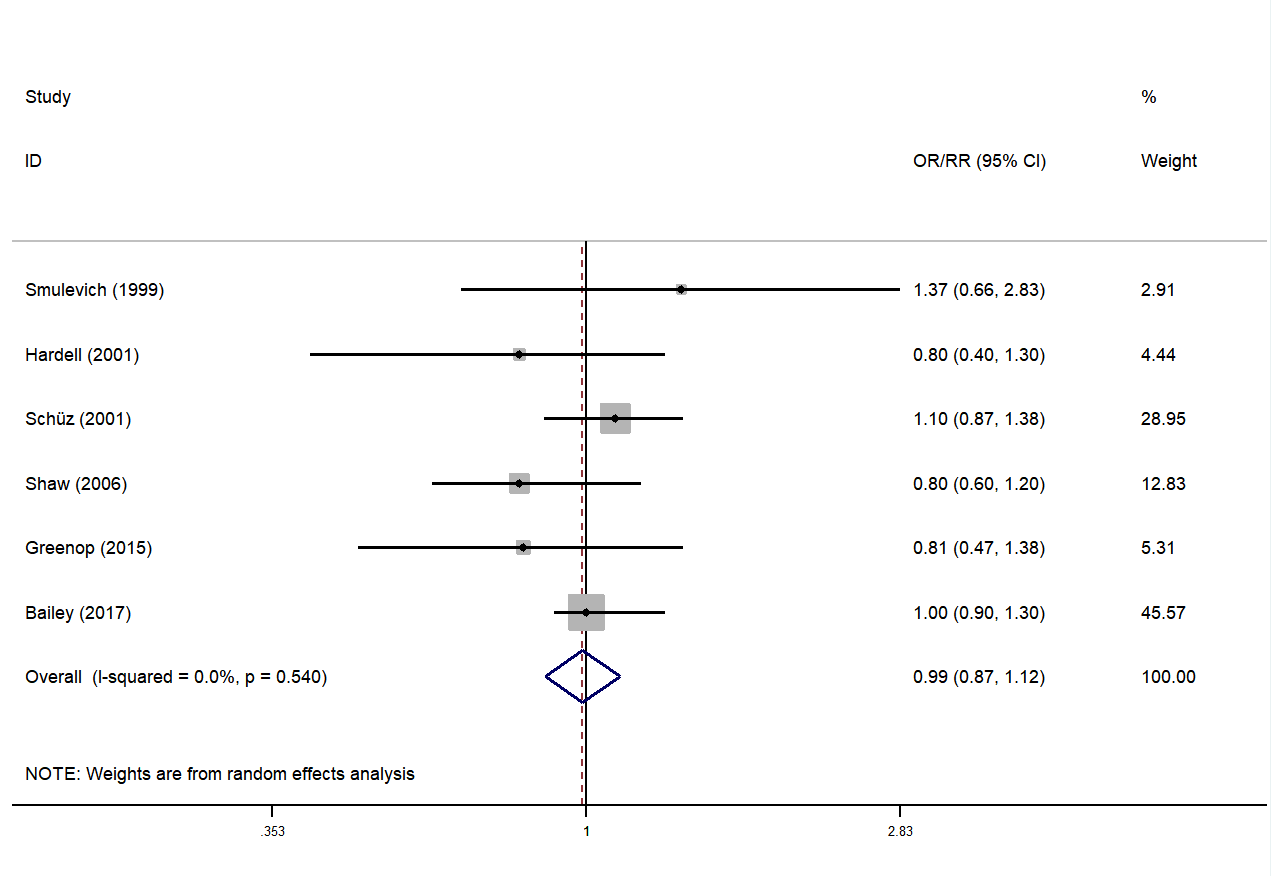


**Fig. S14** Pooled analysis of studies including only children aged 0-14 years old for the for the association of breastfeeding with risk of childhood brain tumors.
